# Counterfactual Mediation Analysis with a Latent Class Exposure

**DOI:** 10.1080/00273171.2024.2335394

**Published:** 2024-05-31

**Authors:** Gemma Hammerton, Jon Heron, Katie Lewis, Kate Tilling, Stijn Vansteelandt

**Affiliations:** a Centre for Academic Mental Health, Population Health Sciences, Bristol Medical School, University of Bristol; b Medical Research Council Integrative Epidemiology Unit, University of Bristol; c Population Health Sciences, Bristol Medical School, University of Bristol; d Health Protection Research Unit in Behavioural Science and Evaluation (HPRU BSE), University of Bristol; e Centre for Neuropsychiatric Genetics and Genomics, Division of Psychological Medicine and Clinical Neurosciences, School of Medicine, Cardiff University; f Department of Psychiatry, Brigham & Women’s Hospital, Harvard Medical School; g Department of Applied Mathematics, Computer Science and Statistics, Ghent University

**Keywords:** Counterfactual mediation, latent class, simulation, pseudo class draws, ALSPAC

## Abstract

Latent classes are a useful tool in developmental research, however there are challenges associated with embedding them within a counterfactual mediation model. We develop and test a new method “updated pseudo class draws (uPCD)” to examine the association between a latent class exposure and distal outcome that could easily be extended to allow the use of any counterfactual mediation method. UPCD extends an existing group of methods (based on pseudo class draws) that assume that the true values of the latent class variable are missing, and need to be multiply imputed using class membership probabilities. We simulate data based on the Avon Longitudinal Study of Parents and Children, examine performance for existing techniques to relate a latent class exposure to a distal outcome (“one-step,” “bias-adjusted three-step,” “modal class assignment,” “non-inclusive pseudo class draws,” and “inclusive pseudo class draws”) and compare bias in parameter estimates and their precision to uPCD when estimating counterfactual mediation effects. We found that uPCD shows minimal bias when estimating counterfactual mediation effects across all levels of entropy. UPCD performs similarly to recommended methods (one-step and bias-adjusted three-step), but provides greater flexibility and scope for incorporating the latent grouping within any commonly-used counterfactual mediation approach.

## Introduction

Mixture models are a useful and commonly used tool in developmental research, where they are used to identify unobserved groups (“latent classes”) that group individuals so that those within a latent class are more similar to each other than those in other classes. Mixture models can take many forms including cross-sectional models such as latent class and latent profile analysis and longitudinal models such as growth mixture models or longitudinal latent class analysis. They all consist of a measurement model (the relationship between the indicator variables and the underlying latent variable) and often also a structural model (the distribution of the latent variable and relationship with auxiliary variables), see (Berlin, Parra et al., 2014; Berlin, Williams et al., 2014) for accessible introductions to mixture modeling. Research questions often focus on the association between a latent class exposure and a distal outcome (for example, the association between developmental trajectories of childhood conduct problems and later alcohol problems (Bevilacqua et al., 2018)). A natural extension to this research question is to ask what mechanisms may be on the causal pathway between the latent class exposure and distal outcome, however there are challenges associated with embedding latent classes within a broader statistical model.

All mediation models consist of an exposure *X*, outcome *Y*, and one or more mediators *M* which lie on the causal pathway between *X* and *Y*. The structural equation modeling (SEM) framework would permit exposure *X* to take the form of a nominal latent grouping and indirect effects *via M* could be estimated using traditional mediation methods, e.g., the product of coefficients strategy (MacKinnon et al., 2002). This approach is often used to examine mediation models in developmental research, e.g., (Baskin-Sommers & Baskin, 2016; Murphy et al., 2014; Sacks et al., 2017). However, these studies used the class assignment probabilities to assign individuals to their most likely class, e.g., modal class assignment (Vermunt, 2010) before estimating the mediation model. This multi-step approach is computationally simpler, however it means that the uncertainty in latent class assignment is not taken into account which can attenuate parameter estimates and standard errors (SE), particularly when the class separation (entropy) is poor (Bakk et al., 2013; Heron et al., 2015; Vermunt, 2010).

Estimating the measurement model for the latent class exposure at the same time as estimating the mediation model (e.g., using a “one-step” model; (Bandeen-Roche et al., 1997) prevents the bias seen in parameter estimates and SE when treating the latent classes as an observed variable. However, a one-step model will treat the mediator and outcome as additional indicators of the latent class variable meaning that the number, composition, and meaning of the latent classes can shift across models with and without the distal outcomes if the additional assumptions being made (e.g., regarding the within-class distribution of the distal outcome) are violated (Bakk et al., 2013; Nylund-Gibson et al., 2019). Additionally, the complexity of a model that simultaneously estimates the latent classes alongside the mediation model can increase the risk of model non-convergence (Vermunt, 2010). These limitations of the one-step model, led to the development of several “bias-adjusted three-step” methods which aim to estimate the associations between latent classes and distal outcomes without bias by maintaining the latent nature of *X*, but also preserving the latent class distribution from the unconditional model (Vermunt, 2010). One commonly used bias-adjusted method is the modified Bolck, Croon and Hagenaars (BCH) three-step approach (Bolck et al., 2004). This method uses a weighted multiple group analysis, where the groups correspond to the latent classes and the weights reflect the measurement error of the latent class variable. Therefore, this method accounts for the uncertainty in latent class assignment but usually prevents the shift in the number, composition, or meaning of the classes that can happen when including distal outcomes using the one-step method (Bakk et al., 2013; Bakk & Vermunt, 2016; Vermunt, 2010).

Another group of methods exist to relate a latent class exposure to a distal outcome, which treat the latent classes as missing data, rather than a problem of misclassification (as with the bias-adjusted three-step approaches) (Bakk & Kuha, 2021). These methods (known as multiple pseudo class draws; PCD), assume that the true values of the latent class variable *X* are missing, and therefore need to be multiply imputed using the class membership probabilities (Bakk & Kuha, 2021; Bray et al., 2015; Wang et al., 2005). This approach was initially shown to result in biased parameter estimates and SE (similar or worse than modal class assignment) because the imputation of the latent classes was only conditional on the latent class indicators and not the external variables in the analysis model (Bray et al., 2015), resulting in “omitted outcome” bias which is well-known in the multiple imputation literature (Collins et al., 2001). This led to the development of “inclusive PCD” (referred to hereafter as incPCD) which includes all analysis variables as covariates when deriving the latent classes and imputes class membership using the probabilities exported from this conditional latent class model (Bray et al., 2015). Simulation studies have shown that this method prevents the bias seen with “non-inclusive PCD” (referred to hereafter as nPCD), assuming model assumptions are met (Bray et al., 2015; Dziak et al., 2016); however, it is subject to similar limitations as the one-step model (e.g., a complex class derivation model which can lead to estimation problems and risk of distorting the classes) (Bakk & Kuha, 2021; Dziak et al., 2016). For a recent, comprehensive review on existing methods to relate latent classes to a distal outcome and their strengths and limitations see (Nylund-Gibson et al., 2019).

In recent years, there has been a paradigm shift in the approach to mediation modeling with the advantages of using a counterfactual framework being highlighted. Specifically, the counterfactual framework provides a general definition of mediation effects using non-parametric causal effect definitions (such as the average difference between two potential outcomes) and explicitly outlines the formal assumptions required (see Supplement 1) to enable causal inference (VanderWeele, 2015, 2016; Vanderweele & Vansteelandt, 2009). This framework holds advantages over the traditional approach to mediation analysis, for example, assumptions regarding linear effects can be relaxed, meaning that exposure-mediator interactions and non-continuous mediators and outcomes can be incorporated. Additionally, more recent methodological developments incorporate SEM within a counterfactual framework giving maximum flexibility for mediation analyses (De Stavola et al., 2015; Muthén, 2011; Muthén & Asparouhov, 2015). For comprehensive descriptions on counterfactual mediation, including the definition of causal effects and assumptions required see (De Stavola et al., 2015; Muthén, 2011; Muthén & Asparouhov, 2015; VanderWeele, 2015, 2016; Vanderweele & Vansteelandt, 2009).

Currently the only established methods for incorporating latent classes (a nominal latent variable) into counterfactual mediation models include using the regression-based approach to counterfactual mediation (Hsiao et al., 2021; Valeri & VanderWeele, 2013), or direct application of the mediation formula (McLarnon & O’Neill, 2018; Muthén, 2011; Muthén et al., 2017; Muthén & Asparouhov, 2015; Pearl, 2012), both implemented within a SEM framework. These counterfactual mediation methods are limited by reliance on a continuous or rare binary outcome (regression-based approach), deliver conditional effects when adjusting for confounders, and difficulty incorporating multiple mediators or intermediate confounders. They can also be complex to implement manually, and the user-friendly packages that exist (e.g., *paramed* in Stata and *model indirect* in M*plus*) cannot be used with a latent class *X*. (Hsiao et al., 2021) provide a simulation study and empirical demonstration comparing one-step and bias-adjusted three-step methods to estimate counterfactual mediation effects using the regression-based approach with a latent class mediator and outcome. (McLarnon & O’Neill, 2018) provide an accessible and comprehensive tutorial on estimating counterfactual mediation effects with a latent class variable using bias-adjusted three-step methods and the mediation approach outlined in (Muthén, 2011; Muthén et al., 2017; Muthén & Asparouhov, 2015).

Alternative counterfactual mediation methods exist that can overcome the limitations of the regression-based approach and direct application of the mediation formula, for example methods using inverse probability weighting or Monte Carlo simulation (Daniel et al., 2011; De Stavola et al., 2015; VanderWeele et al., 2014; VanderWeele & Vansteelandt, 2014). Combining these mediation methods with either a one-step or bias-adjusted three-step approach to incorporate a latent class *X* would be difficult, either because the methods have not been developed, or because they would involve multiple steps (where the number, composition, or meaning of the latent classes could change across each step). With a one-step model, the latent class *X* is continually being recreated in every step of the analysis meaning that it cannot be meaningfully compared across analysis steps (Bakk & Kuha, 2021). Additionally, using a one-step or bias-adjusted three-step approach to incorporate a latent class *X* prevents the use of many ready-made counterfactual mediation packages, for example in Stata (*paramed; medeff; gformula*) and R (*mediation; medflex*).

Here we introduce and test a new method, which we refer to as “updated pseudo class draws” (uPCD), to examine the association between a latent class exposure and distal outcome that could easily be extended to allow the use of any counterfactual mediation method. This method extends an established approach to relate a latent class exposure to a distal outcome (nPCD) that is known to result in biased parameter estimates and SE (Bray et al., 2015). Similar to nPCD, our updated approach only requires an unconditional latent class model to be performed in an initial step; however, the class assignment probabilities are derived in a second step using not only the latent indicators and parameters from the unconditional model, but also information from all external variables in the analysis model. This should reduce bias in parameter estimates as the new class assignment probabilities are conditioned on individuals’ responses to the external variables as well as the latent class indicators (Bray et al., 2015). The idea behind uPCD is very closely based on the existing incPCD approach developed by Bray et al. (2015) where the bias in parameter estimates is eliminated through including all external variables from the analysis model as covariates in the initial model when the latent classes are derived. The difference between incPCD and uPCD is when and how the class membership probabilities are derived. In contrast to incPCD, our updated approach does not require all external variables from the analysis model to be included in the initial model where the latent classes are derived, preventing the risk of distorting the classes and any estimation problems from a complex class derivation model. Another advantage of uPCD is that the uncertainty in class assignment probabilities can be taken into account through perturbing the parameter estimates (from the unconditional latent class model and from the regression models for the mediator and outcome) before deriving the assignment probabilities (further detail is given in Supplement 3). This step is important to prevent underestimated SE which have been shown in simulation studies when the uncertainty in the class assignment probabilities is not accounted for in the final analysis model (Bakk et al., 2013; Vermunt, 2010).

The aim of the study is to perform a simulation study (using a four-class latent class exposure with binary indicators, binary mediator, and binary outcome) as a proof-of-concept, and compare mediation effects estimated using uPCD to effects estimated using existing methods that are either frequently used in practice (one-step, modal class assignment), currently recommended (bias-adjusted three-step), or no longer used in practice, but closely related to uPCD (nPCD, incPCD). We hypothesize that uPCD will estimate mediation effects (and their SE) without bias across scenarios with high, medium, and low class separation (entropy). We will also use an applied example to show the application and performance of uPCD (alongside existing methods) when using real data from the Avon Longitudinal Study of Parents and Children (ALSPAC) on developmental trajectories of childhood conduct problems (Barker & Maughan, 2009), illegal drug use, and high internalizing symptoms.

## Simulation study

### Methods

Based on recommendations detailed in Morris et al. (2019) for planning and reporting simulation studies, we have used a structured approach “ADEMP” which involves defining aims, data-generating mechanisms, estimands, methods, and performance measures.

#### Aims

We aim to offer a proof-of-concept, by evaluating the performance of uPCD in estimating counterfactual mediation effects in three simple settings with a four-class latent class exposure, binary mediator, and binary outcome, which differ according to levels of latent class separation. We will compare bias (in parameter estimates for mediation effects and their model SE), precision, and coverage of confidence intervals to five existing methods to relate a latent class exposure to a distal outcome.

#### Data generating mechanisms

Given that the purpose of this simulation study is to offer a proof-of-concept, we only consider three data generating mechanisms. For all three, data are simulated on *n_ob_*_s_ = 5,000 which represents the approximate sample size in commonly-used cohort studies. We also perform a sensitivity analysis, simulating data on *n_ob_*_s_ = 2,000. The three data generating mechanisms differ only based on entropy, a measure of separation between latent classes (ranging from 0 to 1) with higher values denoting better class separation (Ramaswamy et al., 1993). Latent class separation was varied to produce models with (a) good (∼0.90), (b) medium (∼0.80), and (c) poor (∼0.70) levels of entropy as has been used previously to define measurement quality (Dziak et al., 2016). The Monte Carlo routine in M*plus* version 8.4 (Muthén & Muthén, 2017) was used to simulate the data with an input seed of 3454367. Supplementary Table 1 shows the classification probabilities for the most likely latent class membership by latent class, highlighting that the poorest separation of classes is seen when comparing the “Adolescent Onset” and “Childhood Limited” classes to the “Low” class. It is important to consider the pairwise class separation in addition to overall entropy, given that not all class comparisons will have the same degree of accuracy (Heron et al., 2015). These classification matrices were taken from three large simulated datasets (*n_ob_*_s_ = 1,000,000) with poor, medium, and good entropy, given the difficulty of averaging these matrices across 500 simulated datasets from each entropy level.

**Figure 1.**
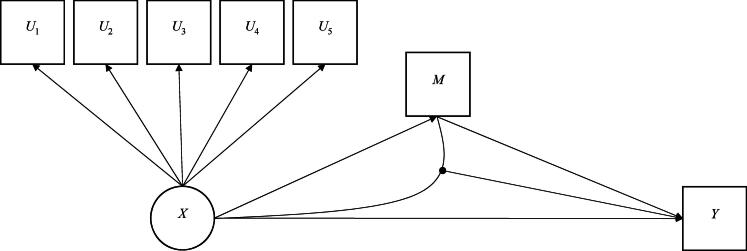
Simulated mediation model; *U*_p_ = binary latent class indicators; *X* = latent class exposure; *M* = binary manifest mediator; *Y* = binary manifest outcome.

Data were simulated under a mediation model with a four-class latent class exposure. The simulated model is shown in Figure 1. The interest is in how a child’s trajectory of conduct problems (exposure *X*) may lead to problematic alcohol use in late adolescence (outcome *Y*), and the extent to which this may be explained through associating with deviant peers in the intervening period (mediator *M*). Furthermore, the magnitude of association between childhood conduct problems (*X*) and later alcohol problems (*Y*) is moderated by peer deviance (*M*). Here, we chose to simulate a mediation model including an *XM* interaction, given that the importance of considering the potential for an *XM* interaction within a counterfactual mediation model has been highlighted to prevent any bias in the estimation of the indirect effect (VanderWeele, 2015).

Here we use the ALSPAC birth cohort and previous ALSPAC research to inform various model parameters. Variable distributions are in keeping with previous publications however the associations themselves are merely plausible and not based on any empirical data.

##### Nominal (latent) exposure X

Developmental trajectories of conduct problems (CP) have been described previously (Barker & Maughan, 2009). Repeated binary measurements spanning the ages 4–13 years were derived from the ‘Conduct Problem’ subscale of the Strengths and Difficulties Questionnaire (Goodman, 2001). Longitudinal mixture modeling of these data yielded the classic cat’s-cradle or soldier’s-bed (Sher et al., 2011) set of four trajectories which were termed “Low,” “Childhood Limited” (i.e., probability of conduct problems decreasing with age) “Adolescent Onset” (i.e., probability of conduct problems increasing with age) and “Early-Onset Persistent” (i.e., probability of conduct problems persistently high throughout). In the current study we simulate the essence of these findings using five binary class-indicators and Longitudinal Latent Class Analysis. Class distribution was defined as follows: Early-Onset Persistent (EOP; 8%), Adolescent Onset (AO; 10%), Childhood Limited (CL; 12%), and Low (70%).

##### Binary (manifest) outcome Y

Hazardous alcohol use was defined by a score on the Alcohol Use Disorders Identification Test (AUDIT; (Babor et al., 2001)) of 8 or greater. A prevalence of 38% reflects data from ALSPAC collected at age 16 years.

##### Binary (manifest) mediator M

Associating with deviant peers during mid-adolescence was defined by whether young person has a friend that has committed a serious crime in the last year. A prevalence of 20% reflects self-report ALSPAC data at age 15 years.

Supplementary Figure 1 shows the trajectory shapes across each data generating mechanism (good, medium and poor entropy levels). Table 1 shows the cross-tabulation which was used to define the model parameters (across all levels of entropy). Table 1 led to the following measures of association between the various variables:

**Table 1.** Data used for simulation (sample = 5,000).

Conduct trajectory (*X*)	Peer deviance (*M*)	Hazardous alcohol use (*Y*)			Total
		0: No	1: Yes	% *Y* = 1	
1: EOP	0: No	150	120	44.4%	270
2: AO		200	125	38.5%	325
3: CL		295	180	37.9%	475
4: Low		1950	980	33.4%	2930
1: EOP	1: Yes	50	80	61.5%	130
2: AO		75	100	57.1%	175
3: CL		65	60	48.0%	125
4: Low		325	245	43.0%	570

EOP: Early-Onset Persistent; AO: Adolescent Onset; CL: Childhood Limited.

- Effect of exposure X on mediator M. All three conduct problem classes (EOP, AO and CL versus Low) increase the odds of peer deviance: EOP (odds ratio = 2.48), AO (odds ratio = 2.77), CL (odds ratio = 1.35), Low (reference).
- Effect of mediator M on outcome Y stratified by X. Conduct problem trajectories moderate the effect of peer deviance on hazardous alcohol use: EOP (odds ratio =2.00), AO (odds ratio =2.13), CL (odds ratio =1.51), Low (odds ratio =1.50).

Table 2 shows the mediation effects of the conduct problem trajectories (EOP, AO and CL versus Low) on hazardous alcohol use implied by these values. We present associations between *X*, *M,* and *Y* as odds ratios (as these are estimated using logistic regression models). However, mediation effects are derived from potential outcome probabilities, which can be used to calculate the odds ratio, risk ratio, or risk difference. Here, we present risk ratios given the ease of interpretation compared to odds ratios.

**Table 2.** Values for direct and indirect effects based on simulated data in Table 1.

	Estimate (log RR)	RR
*EOP vs Low*		
Total Effect (TE)	0.3567	1.4286
Total Natural Indirect Effect (TNIE)	0.0570	1.0587
Pure Natural Direct effect (PNDE)	0.2996	1.3494
*AO vs Low*		
Total Effect (TE)	0.2513	1.2857
Total Natural Indirect Effect (TNIE)	0.0809	1.0842
Pure Natural Direct effect (PNDE)	0.1704	1.1858
*CL vs Low*		
Total Effect (TE)	0.1335	1.1429
Total Natural Indirect Effect (TNIE)	0.0116	1.0116
Pure Natural Direct effect (PNDE)	0.1220	1.1297

EOP: Early-Onset Persistent; AO: Adolescent Onset; CL: Childhood Limited; RR: Risk Ratio.

#### Estimands

Our estimands are mediation effects, including the total natural indirect effect (TNIE), the pure natural direct effect (PNDE), and the total effect (TE). Mediation effects were estimated in a SEM framework based on two logistic regression models. Equation (1) involves binary observed outcome *Y* (hazardous alcohol use), binary observed mediator *M* (associating with deviant peers), and nominal latent exposure *X* (four development trajectories of conduct problems), where *X_1_, X_2_* and *X_3_* are three dummy variables for the latent exposure *X*:

(1) P(Y=1 | X,M)=expit(β0+β1M+β2X1+β3X2+β4X3+β5X1M+β6X2M+β7X3M)

Equation (2) involves binary observed mediator *M*, and nominal latent exposure *X*:

(2) P(M=1 | X)=expit(α0+α1X1+α2X2+α3X3)

Here, we compare three “risk” classes (EOP, AO, CL) to a reference class (Low); however, there are other parameters which could be derived which compare pairs of non-reference classes (e.g., AO versus CL). Mediation effects were derived using direct application of the mediation formula (Muthén, 2011; Pearl, 2012). Further detail is given in Supplement 1. Throughout the manuscript, we refer to the estimate (e.g., log risk ratio for the TE of EOP versus Low conduct problems) as an approximation of the estimand (the quantity of interest).

#### Methods

Each simulated dataset was analyzed using six different techniques to relate a latent class exposure to a distal outcome including: (i) “one-step” estimation (latent nominal exposure with multiple binary indicators), (ii) “bias-adjusted three-step” (here we used the modified Bolck, Croon and Hagenaars (BCH) approach (Bolck et al., 2004), (iii) “modal class assignment” (manifest nominal exposure), (iv) “non-inclusive PCD” (nPCD; imputed manifest nominal exposure), (v) “inclusive PCD” (incPCD; imputed manifest nominal exposure), and (vi) “updated PCD” (uPCD; imputed manifest nominal exposure). In all scenarios, there would usually be an initial step of class enumeration; however, we will not consider this step here, and assume that the presence of a four-class solution is known. Supplementary Figure 2 shows path diagrams for each of the six methods. We use *X* to denote the underlying nominal latent class variable with categories *x*** **=** **1, …, *k*, and *W* for the observed nominal variable generated using an individual’s class assignment probabilities. Latent class indicators are denoted by ***U*** which is a vector of *p* observed binary manifest variables which we assume to be mutually independent, conditional on *X*.

Latent class analysis (LCA) consists of a structural model and a measurement model. In an unconditional LCA, the structural model relates to the unconditional probability of belonging to latent class *x*, P(X=x). The measurement model relates to the class-specific probability of a pattern of responses to the latent class indicators, $P(U_{j}\;|\;X=x),$ where $U_{j},j=1,\ldots ,p$ represents the responses for the latent class indicators. Class assignment probabilities, $P(X=x\;|U_{1},\ldots U_{p}),$ are a function of these two types of probabilities, which provide the probability of class membership for each individual in the sample (in the interest of clarity, we have not used *i* to represent the individual). All individuals with the same pattern of observed data (latent class indicators) have the same within-class probabilities and the same class-assignment probabilities. In the structural model of a conditional LCA, the latent class variable, *X,* can be related to covariates and/or distal outcomes, as in Equations (1) and (2) above.

##### One-step

In the one-step approach, we estimated the model used in the simulation. Here, a single model is used to simultaneously estimate the relationships between the latent class variable, *X*, and the observed latent class indicators, ***U***, as well as the class-specific distal outcome distributions for the mediator, *M*, and outcome, *Y* (Vermunt, 2010). In other words, the mediation effects of the latent class exposure on the outcome were estimated by incorporating the mediator and outcome in the original mixture model. This approach treats the distal outcomes as additional indicators of the latent class variable. This method would be expected to be unbiased across all three levels of class separation (good, medium, and poor entropy).

##### Bias-adjusted three-step

In bias-adjusted three-step methods, an unconditional LCA is performed (step 1) and participants are assigned to their most likely class to create the nominal observed variable *W* (step 2). When modal assignment is used, participants are assigned to the class for which they have the highest probability of belonging, according to the class assignment probabilities, $P(X=x\;|U_{1},\ldots U_{p}).$ In step 3, the structural model is estimated using the nominal variable, *W,* as the exposure in place of the latent class variable *X,* but allowing for the misclassification error introduced in step 2. This is given by the conditional probabilities for an assigned class membership *w,* given the true latent class membership *x*: P(W=w |X=x).

We used the modified BCH approach which uses a weighted multiple group analysis, where the groups correspond to the latent classes and the weights correspond to the inverse logits of the classification errors, P(W=w |X=x), for each individual, reflecting the measurement error of the latent class variable. For further detail on this method see (Bakk et al., 2013; Bakk & Vermunt, 2016; Vermunt, 2010). This method would be expected to be unbiased across all three levels of class separation examined here (scenario a, b and c), but may show bias when class separation is poorer than scenario c, particularly if the uncertainty in the weights for latent class assignment is not taken into account (Bakk et al., 2014; Bakk & Kuha, 2021).

##### Modal class assignment

Also referred to as classify-analyze (Bray et al., 2015; Nylund-Gibson et al., 2019) and standard three-step approach (Bakk et al., 2013; Vermunt, 2010). In modal class assignment, step 1 (unconditional LCA) and step 2 (modal assignment) are identical to the bias-adjusted three-step approach above, but in step 3, the misclassification error introduced in step 2 is not taken into account. After performing the unconditional LCA, we assigned each participant to their most likely class to create the nominal variable *W*, and used this as the exposure in the mediation model in place of the latent class variable *X*. This method would only be expected to be unbiased for good entropy and we would expect estimates to be biased and overly precise, particularly with poor entropy. This is due to individuals being forced into their most likely class, and then treating this as an observed variable, rather than taking into account the uncertainty of the classification, as is done in the bias-adjusted three-step approaches.

The three final methods (iv, v, and vi) are all based on multiple PCD, meaning that individuals are randomly classified into latent classes multiple times based on their class assignment probabilities. For incPCD and nPCD we generated 40 imputed datasets, whereas for uPCD, we generated 80 imputed datasets. To decide on the number of datasets to impute, we calculated the Monte Carlo error for each parameter in the regression model for *Y* and the regression model for *M* (between imputation variance divided by the number of imputed datasets, square rooted), and then calculated the percentage of the SE for the same regression parameter, e.g., (Monte Carlo error/SE) × 100 (see Supplement 2). The number of datasets to impute was chosen to achieve Monte Carlo errors that were no more than 10% of the SE for each parameter in the regression models (White et al., 2011). For all three methods, the mediation model was estimated within each imputed dataset and results were pooled using Rubin’s rules for multiple imputation (Rubin, 1987).

##### Non-inclusive pseudo class draws (nPCD)

In nPCD, an unconditional LCA is performed (as in modal class assignment) but instead of using the class assignment probabilities to assign participants to their most likely class, these are used instead to multiply impute class membership by taking random draws from the multinomial distribution defined by the class assignment probabilities (Bray et al., 2015; Nylund-Gibson et al., 2019; Wang et al., 2005). Here, we use the class assignment probabilities, $P(X=x\;|U_{1},\ldots U_{p}),$ from the unconditional LCA to randomly assign each participant to a class *W*** **=** **1, …, *k*, 40 times to generate 40 imputed values of *W* to use in the subsequent mediation model. This method would only be expected to be unbiased for good entropy (scenario a) and we would expect estimates to be biased and overly-precise, particularly with poor entropy (scenario c), due to using class assignment probabilities from an unconditional LCA (Bray et al., 2015; Collier & Leite, 2017).

##### Inclusive pseudo class draws (incPCD)

In incPCD, a conditional LCA is performed with all variables from the subsequent analysis model included as covariates predicting latent class membership. The calculation of the class assignment probabilities is then conditioned on an individual’s vector of responses to the covariates, in addition to the latent class indicators (Bray et al., 2015). Here, we estimated a conditional LCA with the binary mediator *M* and outcome *Y* as covariates predicting latent class membership, and exported class assignment probabilities from this conditional model, P(X=x | Y=y,M=m,U), along with additional (auxiliary) variables required for subsequent analyses. These class assignment probabilities were used to randomly assign each participant to a class *W*** **=** **1, …, *k*, 40 times to generate 40 imputed values of *W* to use in the subsequent mediation model. This method was developed to address the bias in the nPCD model that was due to excluding the analysis variables when deriving the latent classes. IncPCD has been shown to perform well provided model assumptions are met (Bray et al., 2015). Therefore, we would expect incPCD to be unbiased across all three levels of class separation examined here. However, some bias may be introduced given that it will not be possible to include the exposure-mediator interaction as a covariate in the latent class derivation model, meaning that it will be incompatible with the analysis model.

##### Updated pseudo class draws (uPCD)

In uPCD, rather than estimating a conditional LCA (with the distal outcomes, e.g., *M* and *Y*, as covariates) and exporting the class assignment probabilities from this model, an unconditional LCA was used and the class assignment probabilities, P(X=x | Y=y,M=m,U), were derived in a second step. This brings two advantages over incPCD. First, it prevents the need for a complex class measurement model which can lead to estimation problems and distorted latent classes. Second, it allows the uncertainty in the class assignment probabilities to be taken into account in the final analysis model. Assuming the class assignment probabilities are known (which is standard practice in methods which use the assignment probabilities) can result in underestimated standard errors, particularly with a small sample size and poorly separated latent classes (Bakk et al., 2013; Vermunt, 2010). The class assignment probabilities, P(X=x | Y=y,M=m,U), were derived using the latent class indicators, parameters from the unconditional LCA (latent class intercepts and within-class thresholds), and also the parameters (α,β) from Equations (1) and (2) above (representing the relationship between the latent classes with the mediator and outcome). Since α,β is unknown, the procedure needs to be iterated and in doing so, estimates of α,β and the latent class parameters can be perturbed based on their sampling distribution to prevent underestimated standard errors. The steps involved are outlined briefly below, with further detail provided in Supplement 3, and a schematic for the iterative procedure provided in Supplement 3 Figure 1.

- Step 0: an unconditional LCA was performed and the latent class intercepts, within-class thresholds, and their (co)variance matrix were saved to use in step 2 below.
- Step 1: a logistic regression model was performed for *Y* [Equation (1) above] but with all β except for $\beta_{0},\beta_{1}$ set to zero and a logistic regression model was performed for *M* [Equation (2) above] but with all α  except for $\alpha_{0}$ set to zero. Estimates of $\beta_{0}{,\beta }_{1}$ and $\alpha_{0}$ from the logistic regression models were perturbed with Gaussian noise of mean zero and values from the variance-covariance matrix of these parameter estimates.
- Step 2: the latent class intercepts and within-class thresholds from the unconditional LCA (step 0) were perturbed and combined with the previous values of α,β to calculate the class assignment probabilities, P(X=x | Y,M,U) for *x* = 1, …, *k* for each participant.
- Step 3: the class assignment probabilities were used to randomly assign each participant to a class *W* = 1, …, *k*, and the logistic regression models for P(Y=1 | W,M) and for P(M=1 | W) were performed, using *W* in the place of the latent class exposure *X*. Estimates of α,β from the logistic regression models were perturbed.
- Step 4: steps 2 and 3 were repeated until convergence of α,β. As a result of perturbation, we did not expect convergence to a single value, but to a stable distribution.
- Step 5: after 20 cycles of iterations (and an initial burn in of 100 iterations), the last imputed values of *W* were stored and the process started again from step 2. This process was repeated to generate 80 imputed values of *W* to use in the subsequent mediation model.

This method would be expected to be unbiased across all three levels of class separation examined here (scenario a, b and c), with precision similar to the one-step model given that the uncertainty in the class assignment probabilities is taken into account.

#### Performance measures

We assessed bias, percentage bias, coverage, bias-eliminated coverage, empirical and model-based SE for the estimators. Bias quantifies whether the estimator targets the true value θ on average and is calculated as the mean difference between the true value θ and the estimated value $\hat{\theta }.$ Percentage bias is calculated as the bias divided by the true value and multiplied by 100. Coverage is the probability that a 95% confidence interval contains the true value θ. Under coverage can be a result of bias, a model SE smaller than the empirical SE, a non-normal distribution for $\hat{\theta },$ or the estimated variance of ${\hat{\theta }}_{i}$ for the i^th^ replication being too variable. Bias-eliminated coverage accounts for the role of bias in the coverage by evaluating whether 95% confidence intervals include the average estimate $\hat{\theta }.$ The empirical SE of $\hat{\theta }$ is the standard deviation of $\hat{\theta }$ over the replications. The model based SE is the average of the estimated SE for each replication. Supplement 4 presents a comparison of estimates and estimated SE across all simulated datasets. The model based SE targets the empirical SE (Morris et al., 2019).

Potential non-convergence for uPCD was determined by examining the trace plots for model parameters and cell sizes from cross-tabulations of the exposure, mediator and outcome across all iterations within each simulated dataset. Non-convergence in the trace plots was found for simulated datasets which had a large SE for one or more within-class thresholds in the unconditional latent class model. Therefore, simulated datasets were excluded from the dataset of the estimates when the largest SE for a within-class threshold (representing the class-specific probability for a latent class indicator) from the unconditional latent class model was greater than twice the average of the largest within-class threshold SE across the simulated datasets. The same rule was applied to the within-class threshold SE in the one-step latent class model. Although all methods (apart from one-step) start with running the unconditional model, it is only uPCD that takes account of the uncertainty in the parameters from the unconditional model in subsequent analysis steps.

Bias is our key performance measure of interest, and based on an initial pilot simulation run, we assumed that SD($\hat{\theta }$) ≤ 0.1 for all $\hat{\theta },$ meaning that Var($\hat{\theta }$) ≤ 0.01. Therefore, simulating 400 datasets for each data generating mechanism will give a Monte Carlo SE (MCSE) for the bias of 0.005 which we consider to be acceptable (Morris et al., 2019). Given the potential risk of non-convergence, we simulated 500 datasets for each data-generating mechanism. After exclusions were made based on non-convergence (see results section for details), we confirmed that the MCSE for the bias was below 0.005 for all estimators. After data were simulated, all subsequent analyses were performed in R (version 4.1.1; (R Core Team, 2021). The R package *MplusAutomation* (Hallquist & Wiley, 2018) was used to analyze simulated datasets and the R package *rsimsum* (Gasparini, 2018) was used to analyze the resulting dataset of the estimates. For further detail on using the R package *rsimsum*, including creating plots, see the vignette by Gasparini, 2022 (https://cran.r-project.org/web/packages/rsimsum/vignettes/). For all other packages used (including version number) see the annotated analysis scripts using R and M*plus*, available here https://github.com/gemmahammerton/latentclass-mediation. Additionally, the Supplementary webpage (upcd-for-sim1-poor-entropy) provides code and output for uPCD using the first simulated dataset with poor entropy.

## Results

### Convergence

Whilst no estimates or SE were missing, we excluded some simulated datasets due to very large SE for some within-class thresholds (representing within-class probability for a latent class indicator) in either the unconditional latent class model or the one-step model. When this was the case, the simulated dataset was excluded across all six methods. Nine percent of simulated datasets with good entropy (*n* = 16 for unconditional latent class model, *n* = 23 for one-step model, *n* = 4 for both), 13% with medium entropy (*n* = 32 for unconditional latent class model, *n* = 26 for one-step model, *n* = 8 for both), and 15% with poor entropy (*n* = 35 for unconditional latent class model, *n* = 33 for one-step model, *n* = 8 for both) were excluded.

### Exploration of raw results

Scatter plots of estimates versus SE for each method (one-step, bias-adjusted three-step, modal class assignment, nPCD, incPCD, uPCD), data generating mechanism (good, medium, poor entropy), estimand (TE, TNIE, PNDE), and latent class comparison (EOP versus low, AO versus low, CL versus low) are shown in Supplement 4. Simulated datasets that were excluded based on convergence criteria are shown with a light blue dot, whereas all other datasets are shown with a dark blue dot. The plots show that many of the outliers correspond to datasets that are excluded based on the convergence criteria (light blue dots), particularly for uPCD. However, a few outliers remain when there is poor entropy (across almost all estimands and class comparisons), particularly for the one-step method. This is likely a consequence of using the one-step method to estimate a complex analysis model, which can result in non-convergence (Vermunt, 2010).

### Performance measures

Figure 2 shows the bias and 95% confidence intervals (based on MCSE) by method, data generating mechanism, and latent class comparison, after excluding datasets based on convergence criteria above. Figure 2a shows the bias in the TE, Figure 2b plots the bias in the TNIE, and Figure 2c plots the bias in the PNDE. As shown in the Figures, uPCD shows minimal bias which is comparable to one-step and bias-adjusted three-step models across all entropy levels, estimands, and class comparisons. Additionally, incPCD shows minimal bias with the exception of the TNIE of AO versus Low conduct problems where there is bias toward the null. The greatest bias is for nPCD, followed by modal class assignment, with bias toward the null, particularly when the entropy is poor. The only exception to this is when the effect size is close to zero (TNIE of CL versus Low conduct problems; true effect = 0.012), and all methods have minimal bias across all entropy levels.

Figure 2.(a) Bias (and 95% confidence intervals based on Monte Carlo standard errors) in the total effect (TE) by method, data generating mechanism, and latent class comparison; *N* = 457 simulated datasets with good entropy (0.9), *N* = 434 simulated datasets with medium entropy (0.8), *N* = 424 simulated datasets with poor entropy (0.7); Methods = one-step, bias-adjusted three-step (bch), modal class assignment (modal), non-inclusive PCD (npcd), inclusive PCD (incpcd), and updated PCD (upcd); data-generating mechanisms = good, medium and poor entropy levels; latent class comparisons = Early-Onset Persistent (1.eop) versus Low: true value = 0.357, Adolescent Onset (2.ao) versus Low: true value = 0.251; Childhood Limited (3.cl) versus Low: true value = 0.134). **(b)** Bias (and 95% confidence intervals based on Monte Carlo standard errors) in the total natural indirect effect (TNIE) by method, data generating mechanism, and latent class comparison; *N* = 457 simulated datasets with good entropy (0.9), *N* = 434 simulated datasets with medium entropy (0.8), *N* = 424 simulated datasets with poor entropy (0.7); Methods = one-step, bias-adjusted three-step (bch), modal class assignment (modal), non-inclusive PCD (npcd), inclusive PCD (incpcd), and updated PCD (upcd); data-generating mechanisms = good, medium and poor entropy levels; latent class comparisons = Early-Onset Persistent (1.eop) versus Low: true value = 0.057, Adolescent Onset (2.ao) versus Low: true value = 0.081; Childhood Limited (3.cl) versus Low: true value = 0.012). **(c)** Bias (and 95% confidence intervals based on Monte Carlo standard errors) in the pure natural direct effect (PNDE) by method, data generating mechanism, and latent class comparison; *N* = 457 simulated datasets with good entropy (0.9), *N* = 434 simulated datasets with medium entropy (0.8), *N* = 424 simulated datasets with poor entropy (0.7); Methods = one-step, bias-adjusted three-step (bch), modal class assignment (modal), non-inclusive PCD (npcd), inclusive PCD (incpcd), and updated PCD (upcd); data-generating mechanisms = good, medium and poor entropy levels; latent class comparisons = Early-Onset Persistent (1.eop) versus Low: true value = 0.300, Adolescent Onset (2.ao) versus Low: true value = 0.170; Childhood Limited (3.cl) versus Low: true value = 0.122).

**Figure:**
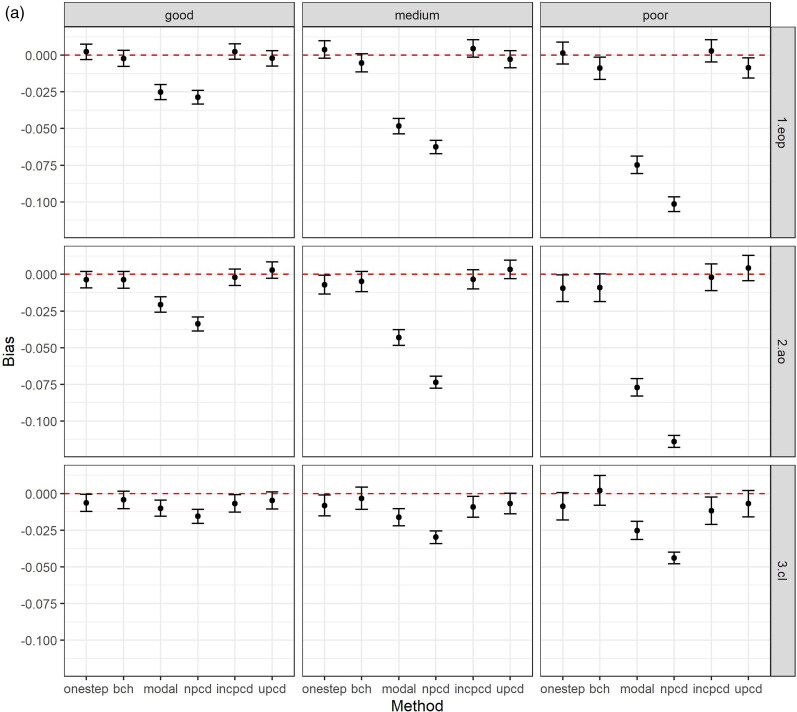

**Figure:**
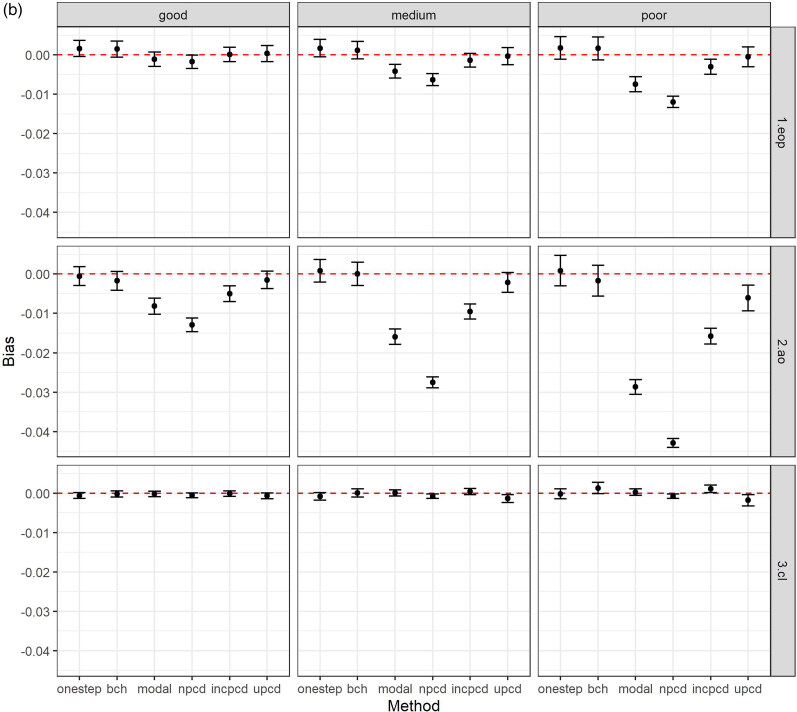

**Figure:**
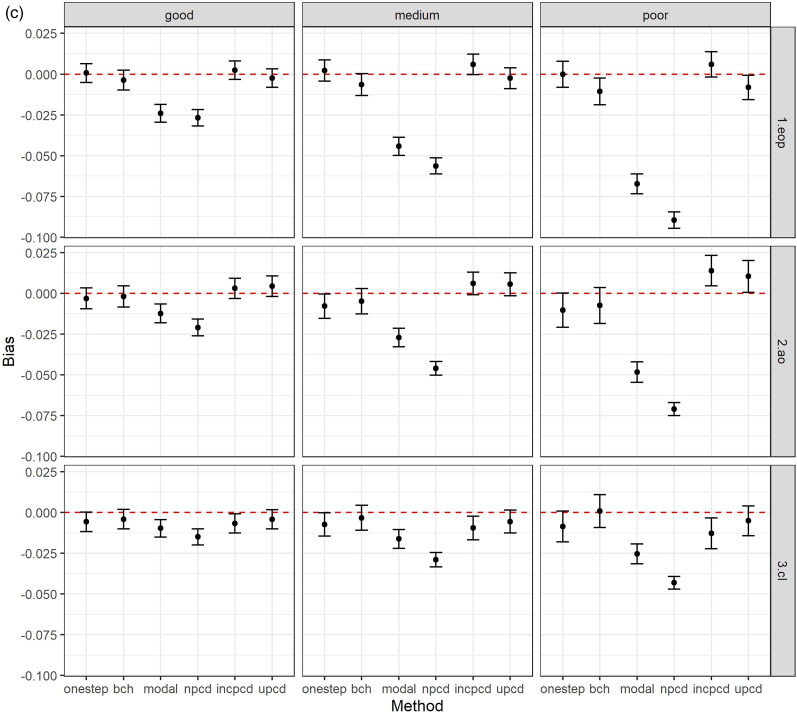

Supplement 5 shows the bias and 95% confidence intervals (based on MCSE) by method, data generating mechanism, and latent class comparison before exclusions (e.g., based on all 500 simulated datasets), and shows that the pattern of bias was very similar.

All performance measures (and MCSE) for each method, data generating mechanism, and estimand (after excluding datasets based on convergence criteria above) are shown in Table 3–5 below for the effects of EOP versus Low conduct problems. In Table 3–5 we only focus on one class comparison (EOP versus Low conduct problems) for clarity. However, performance measures (and MCSE) for each method and data generating mechanism for the TE of AO versus Low conduct problems are shown in Supplementary Table 2 and results for the TE of CL versus Low conduct problems are shown in Supplementary Table 3.

**Table 3.** Performance measures (Monte Carlo standard errors) for each method and data generating mechanism for the total effect (TE) of Early-Onset Persistent versus Low conduct problems (true value = 0.357); *N* = 457 simulated datasets with good entropy (0.9), *N* = 434 simulated datasets with medium entropy (0.8), *N* = 424 simulated datasets with poor entropy (0.7).

Performance measure (for θ)	Data-generating mechanism	One-step	Bias-adjusted three-step (BCH)	Modal class assignment	Non-inclusive pseudo class draws	Inclusive pseudo class draws	Updated pseudo class draws
Bias; % bias	Good entropy	0.002 (0.003); 0.6%	−0.002 (0.003); 0.6%	−0.025 (0.003); 7.1%	−0.029 (0.002); 8.0%	0.002 (0.003); 0.7%	−0.002 (0.003); 0.6%
	Medium entropy	0.004 (0.003); 1.1%	−0.005 (0.003); 1.5%	−0.048 (0.003); 13.6%	−0.063 (0.002); 17.5%	0.005 (0.003); 1.3%	−0.003 (0.003); 0.8%
	Poor entropy	0.002 (0.004); 0.4%	−0.009 (0.004); 2.5%	−0.075 (0.003); 21.0%	−0.102 (0.003); 28.5%	0.003 (0.004); 0.8%	−0.009 (0.004); 2.4%
Coverage	Good entropy	95.8% (0.009)	96.1% (0.009)	93.7% (0.011)	96.7% (0.008)	95.4% (0.010)	96.1% (0.009)
	Medium entropy	95.9% (0.010)	95.9% (0.010)	87.1% (0.016)	92.6% (0.013)	94.7% (0.011)	95.9% (0.010)
	Poor entropy	95.3% (0.010)	95.0% (0.011)	73.1% (0.022)	73.3% (0.021)	89.6% (0.015)	94.6% (0.011)
Bias-eliminated coverage	Good entropy	95.8% (0.009)	95.6% (0.010)	94.5% (0.011)	97.4% (0.007)	95.4% (0.010)	96.1% (0.009)
	Medium entropy	95.6% (0.010)	95.6% (0.010)	95.2% (0.010)	98.4% (0.006)	94.2% (0.011)	95.6% (0.010)
	Poor entropy	95.5% (0.010)	95.3% (0.010)	94.6% (0.011)	99.3% (0.004)	89.9% (0.015)	94.8% (0.011)
Empirical standard error	Good entropy	0.058 (0.002)	0.060 (0.002)	0.055 (0.002)	0.051 (0.002)	0.058 (0.002)	0.057 (0.002)
	Medium entropy	0.063 (0.002)	0.066 (0.002)	0.056 (0.002)	0.049 (0.002)	0.063 (0.002)	0.062 (0.002)
	Poor entropy	0.078 (0.003)	0.080 (0.003)	0.062 (0.002)	0.052 (0.002)	0.080 (0.003)	0.073 (0.003)
Average model standard error	Good entropy	0.060 (0.0002)	0.060 (0.0002)	0.055 (0.0002)	0.059 (0.0001)	0.058 (0.0002)	0.059 (0.0002)
	Medium entropy	0.067 (0.0003)	0.067 (0.0003)	0.057 (0.0002)	0.064 (0.0002)	0.062 (0.0002)	0.065 (0.0002)
	Poor entropy	0.085 (0.001)	0.077 (0.0005)	0.059 (0.0003)	0.070 (0.0004)	0.066 (0.0004)	0.081 (0.001)

**Table 4.** Performance measures (Monte Carlo standard errors) for each method and data generating mechanism for the total natural indirect effect (TNIE) of Early-Onset Persistent versus Low conduct problems (true value = 0.057); *N* = 457 simulated datasets with good entropy (0.9), *N* = 434 simulated datasets with medium entropy (0.8), *N* = 424 simulated datasets with poor entropy (0.7).

Performance measure (for θ)	Data-generating mechanism	One-step	Bias-adjusted three-step (BCH)	Modal class assignment	Non-inclusive pseudo class draws	Inclusive pseudo class draws	Updated pseudo class draws
Bias; % bias	Good entropy	0.002 (0.001); 2.8%	0.001 (0.001); 2.5%	−0.001 (0.001); 2.0%	−0.002 (0.001); 3.1%	0.0001 (0.001); 0.2%	0.0003 (0.001); 0.6%
	Medium entropy	0.002 (0.001); 3.0%	0.001 (0.001); 2.1%	−0.004 (0.001); 7.3%	−0.006 (0.001); 11.0%	−0.001 (0.001); 2.4%	−0.0003 (0.001); 0.5%
	Poor entropy	0.002 (0.001); 3.1%	0.002 (0.001); 2.9%	−0.007 (0.001); 13.1%	−0.012 (0.001); 21.0%	−0.003 (0.001); 5.3%	−0.001 (0.001); 0.9%
Coverage	Good entropy	95.2% (0.010)	95.0% (0.010)	92.8% (0.012)	95.6% (0.010)	96.1% (0.009)	94.7% (0.010)
	Medium entropy	95.4% (0.010)	95.6% (0.010)	92.9% (0.012)	95.9% (0.010)	96.3% (0.009)	96.3% (0.009)
	Poor entropy	95.5% (0.010)	92.9% (0.012)	88.0% (0.016)	92.0% (0.013)	95.5% (0.010)	95.5% (0.010)
Bias-eliminated coverage	Good entropy	95.2% (0.010)	94.5% (0.011)	94.3% (0.011)	96.4% (0.009)	96.1% (0.009)	94.7% (0.010)
	Medium entropy	95.2% (0.010)	95.2% (0.010)	95.4% (0.010)	97.7% (0.007)	96.5% (0.009)	96.3% (0.009)
	Poor entropy	94.3% (0.011)	92.2% (0.013)	90.8% (0.014)	98.1% (0.007)	97.4% (0.008)	95.8% (0.010)
Empirical standard error	Good entropy	0.022 (0.001)	0.023 (0.001)	0.020 (0.001)	0.019 (0.001)	0.020 (0.001)	0.022 (0.001)
	Medium entropy	0.023 (0.001)	0.024 (0.001)	0.018 (0.001)	0.016 (0.001)	0.018 (0.001)	0.023 (0.001)
	Poor entropy	0.030 (0.001)	0.031 (0.001)	0.020 (0.001)	0.015 (0.001)	0.020 (0.001)	0.027 (0.001)
Average model standard error	Good entropy	0.022 (0.0002)	0.022 (0.0002)	0.020 (0.0002)	0.021 (0.0001)	0.022 (0.0002)	0.022 (0.0002)
	Medium entropy	0.026 (0.0002)	0.026 (0.0002)	0.019 (0.0002)	0.021 (0.0002)	0.023 (0.0002)	0.025 (0.0002)
	Poor entropy	0.033 (0.001)	0.031 (0.0004)	0.020 (0.0003)	0.021 (0.0002)	0.025 (0.0003)	0.032 (0.001)

**Table 5.** Performance measures (Monte Carlo standard errors) for each method and data generating mechanism for pure natural direct effect (PNDE) of Early-Onset Persistent versus Low conduct problems (true value = 0.300); *N* = 457 simulated datasets with good entropy (0.9), *N* = 434 simulated datasets with medium entropy (0.8), *N* = 424 simulated datasets with poor entropy (0.7).

Performance measure (for θ)	Data-generating mechanism	One-step	Bias-adjusted three-step (BCH)	Modal class assignment	Non-inclusive pseudo class draws	Inclusive pseudo class draws	Updated pseudo class draws
Bias; % bias	Good entropy	0.001 (0.003); 0.2%	−0.004 (0.003); 1.2%	−0.024 (0.003); 8.0%	−0.027 (0.003); 8.9%	0.002 (0.003); 0.8%	−0.002 (0.003); 0.8%
	Medium entropy	0.002 (0.003); 0.7%	−0.006 (0.003); 2.2%	−0.044 (0.003); 14.8%	−0.056 (0.002); 18.8%	0.006 (0.003); 2.0%	−0.002 (0.003); 0.8%
	Poor entropy	−0.0002 (0.004); 0.1%	−0.011 (0.004); 3.5%	−0.067 (0.003); 22.5%	−0.090 (0.003); 29.9%	0.006 (0.004); 2.0%	−0.008 (0.004); 2.7%
Coverage	Good entropy	95.4% (0.010)	93.4% (0.012)	94.% (0.011)	96.3% (0.009)	94.3% (0.011)	96.3% (0.009)
	Medium entropy	96.1% (0.009)	95.9% (0.010)	91.2% (0.014)	94.5% (0.011)	94.9% (0.011)	96.3% (0.009)
	Poor entropy	95.8% (0.010)	95.3% (0.010)	80.9% (0.019)	84.9% (0.017)	90.8% (0.014)	95.9% (0.010)
Bias-eliminated coverage	Good entropy	95.4% (0.010)	93.4% (0.012)	93.7% (0.011)	97.4% (0.007)	94.7% (0.010)	96.1% (0.009)
	Medium entropy	96.3% (0.009)	95.9% (0.010)	96.1% (0.009)	98.6% (0.006)	95.6% (0.010)	96.8% (0.008)
	Poor entropy	95.8% (0.010)	95.3% (0.010)	94.3% (0.011)	99.1% (0.005)	91.5% (0.014)	95.9% (0.010)
Empirical standard error	Good entropy	0.063 (0.002)	0.066 (0.002)	0.059 (0.002)	0.054 (0.002)	0.062 (0.002)	0.062 (0.002)
	Medium entropy	0.069 (0.002)	0.071 (0.002)	0.059 (0.002)	0.051 (0.002)	0.067 (0.003)	0.067 (0.002)
	Poor entropy	0.084 (0.003)	0.087 (0.003)	0.064 (0.002)	0.053 (0.002)	0.082 (0.003)	0.078 (0.003)
Average model standard error	Good entropy	0.066 (0.0002)	0.066 (0.0002)	0.060 (0.0002)	0.064 (0.0002)	0.064 (0.0002)	0.065 (0.0002)
	Medium entropy	0.074 (0.0003)	0.074 (0.0003)	0.061 (0.0003)	0.069 (0.0002)	0.068 (0.0003)	0.072 (0.0003)
	Poor entropy	0.093 (0.001)	0.086 (0.001)	0.063 (0.0004)	0.074 (0.0004)	0.073 (0.0005)	0.089 (0.001)

Table 3 shows performance measures for the TE of EOP versus Low conduct problems. As shown in Table 3, modal class assignment and nPCD have the largest percentage bias across all entropy levels (21% and 29% respectively, for the poor entropy model). Bias-adjusted three-step (<3%), incPCD (<2%) and uPCD (<3%) have small levels of percentage bias across all entropy levels, which are similar to the levels of percentage bias for the one-step model (<2%). Decreasing entropy increases uncertainty, and this is reflected in the empirical SE which show decreasing precision with decreasing entropy for all methods, except for modal class assignment and nPCD which are overly precise at all entropy levels and also fail to capture the increasing uncertainty. The empirical SE also show that bias-adjusted three-step is slightly less efficient compared with other methods. Model-based SE are close to empirical SE with exception of nPCD (where model-based SE are overestimated by 35% for the poor entropy model) and incPCD (where model-based SE are underestimated by 18% for the poor entropy model). The coverage of nominal 95% confidence intervals is close to 95% for one-step, bias-adjusted three-step, and uPCD across all entropy levels, but there is under-coverage for modal class assignment, nPCD, and incPCD, particularly with poor entropy (modal class assignment = 73%, nPCD = 73%, incPCD = 90%). This is driven by bias for modal class assignment and nPCD, with the bias-eliminated coverage showing over-coverage for nPCD (99% for poor entropy model) which is a result of a model SE greater than the empirical SE. For incPCD, there is still under coverage after accounting for bias in the poor entropy model (90%), which is a result of a model SE smaller than the empirical SE.

Table 4 shows performance measures for the TNIE of EOP versus Low conduct problems. The pattern of results is similar to the TE, with modal class assignment and nPCD having the largest bias particularly for medium and poor entropy models. Bias-adjusted three-step (<3%), incPCD (<6%) and uPCD (<1%) have small levels of percentage bias across all entropy levels, which is similar to the levels of percentage bias for the one-step model (<4%). The empirical SE show that modal class assignment, nPCD, and incPCD are overly precise. Again, model-based SE are close to empirical SE with exception of nPCD (where model-based SE are overestimated by 41% for the poor entropy model) and incPCD (where model-based SE are overestimated by 26% for the poor entropy model). This is in contrast to results for the TE (where model-based SE were overly precise for incPCD). Additionally, model-based SE in the poor entropy model are slightly overestimated for one-step (10%) and uPCD (20%). The coverage of nominal 95% confidence intervals is acceptable for all methods, across all entropy levels with the exception of modal class assignment and nPCD where there is under-coverage, particularly with poor entropy (modal class assignment = 88%, nPCD = 92%). This is partly driven by bias, with the bias-eliminated coverage showing over-coverage for nPCD (98% for poor entropy model). There is also slight under-coverage for bias-adjusted three-step in the poor entropy model (coverage = 93%, bias-eliminated coverage = 92%).

Table 5 shows performance measures for the PNDE of EOP versus Low conduct problems. Again, the pattern of results is similar, with modal class assignment and nPCD having the largest bias, and bias-adjusted three-step (<4%), incPCD (≤2%) and uPCD (<3%) showing small levels of percentage bias, which are similar to the one-step model (<1%). The empirical SE show that bias-adjusted three-step is slightly less efficient compared with other methods, whereas modal class assignment and nPCD are overly precise. Again, model-based SE are close to empirical SE with exception of nPCD (where model-based SE are overestimated by 41% for the poor entropy model). Additionally, model-based SE in the poor entropy model are slightly overestimated for one-step (11%) and uPCD (13%), and slightly underestimated for incPCD (11%). The coverage of nominal 95% confidence intervals is close to 95% for one-step, bias-adjusted three-step, and uPCD across all entropy levels, but there is under-coverage for modal class assignment, nPCD, and incPCD, particularly with poor entropy (modal class assignment = 81%, nPCD = 85%, incPCD = 91%). This is driven by bias for modal class assignment and nPCD, with the bias-eliminated coverage showing over-coverage for nPCD (99% for poor entropy model).

Supplement 6 shows that performance measures using the reduced sample size of *n_ob_*_s_ = 2,000 were similar to the results using *n_ob_*_s_ = 5,000 for each method, data generating mechanism, and estimand. There were similar levels of non-convergence due to a very large SE for some within-class thresholds across *n_ob_*_s_ = 2,000 and *n_ob_*_s_ = 5,000; however, when using *n_ob_*_s_ = 2,000, an additional 3% of datasets with poor entropy were excluded because there was an imputed latent class with a prevalence of zero in at least one of the iterations when running uPCD. With *n_ob_*_s_ = 2,000, there was slightly greater bias across all entropy levels and methods and slightly more under-coverage for bias-adjusted three step, modal class assignment, and incPCD with the smaller sample size. Additionally, the model-based standard errors showed more over-estimation with the smaller sample size, particularly for the one-step model with poor entropy.

## Applied example

### Methods

#### Sample

ALSPAC is an ongoing birth cohort which was set up to examine genetic and environmental determinants of health and development (Boyd et al., 2013). ALSPAC recruited pregnant women resident in Avon, UK with expected dates of delivery between 1st April 1991 and 31st December 1992. Of the 14,541 initial pregnancies, there was a total of 14,676 fetuses, resulting in 14,062 live births and 13,988 children who were alive at 1 year of age (of which 179 were twins). Parents and children have been followed up regularly since recruitment *via* questionnaire and clinic assessments. In the current study, data were used from follow-ups with mothers and young people up to age 18 years. We included those with complete data on the outcome, mediator, confounders and at least one latent class indicator for the exposure (*N*** **=** **3,039). Further details on the sample characteristics and methodology have been described previously (Boyd et al., 2013; Fraser et al., 2013), and detailed information about ALSPAC can be found on the study website (http://www.bristol.ac.uk/alspac). For information on all available ALSPAC data see the fully searchable data dictionary (http://www.bristol.ac.uk/alspac/researchers/our-data/). Written, informed consent was obtained from all mothers who entered the ALSPAC study, and ethical approval for the study was obtained from the ALSPAC Ethics and Law committee (IRB00003312) and the Local Research Ethics Committees. The ethics committee specifically approved the questionnaires and the clinic testing protocols including the methods of gaining consent.

#### Measures

Here the exposure is developmental trajectories of childhood conduct problems from age 4 to 13 years, the mediator is any illicit drug use up to age 18 years, and the outcome is the presence (versus absence) of current internalizing symptoms (depression or generalized anxiety) at age 18 years. Sex and a sociodemographic cumulative risk score are confounders. An exposure-mediator interaction is hypothesized. Mediation analyses make assumptions about the causal ordering of the exposure, mediator and outcome which are easier to justify when there is also a temporal ordering of these variables. In our applied example, the mediator and outcome are both assessed at age 18 years, but we make the assumption that lifetime illicit drug use up to age 18 years is likely to precede current internalizing symptoms at age 18.

##### Nominal (latent) exposure X

Here we use the developmental trajectories of conduct problems (CP) from age 4 to 13 years derived previously (Barker & Maughan, 2009). Entropy for this model was 0.71 and class distribution was as follows: Early-Onset Persistent (EOP; 9%), Adolescent Onset (AO; 5%), Childhood Limited (CL; 21%), and Low (65%).

##### Binary (manifest) outcome Y

Symptoms of depression or generalized anxiety disorder (GAD) were assessed at a focus clinic with the young people at approximately age 18 years using a computerized version of the Clinical Interview Schedule Revised (CIS-R; (Lewis et al., 1992)). The CIS-R is a computerized interview that derives a diagnosis of depression and GAD according to ICD-10 criteria (World Health Organisation, 1993). A binary variable indicating the presence of an internalizing disorder (depression or GAD) or subthreshold symptoms was taken as the outcome measure (prevalence = 13%).

##### Binary (manifest) mediator M

Illicit drug use was assessed during the same focus clinic at age 18 years. The young people were asked about their lifetime use of cannabis, cocaine, amphetamine-type stimulants, inhalants, hallucinogens, opioids and other injected illegal drugs. A binary variable was created representing the lifetime use of any illegal drug (prevalence = 41%).

##### Confounders

Data on sociodemographic factors were collected during pregnancy and perinatal assessments with mothers and included maternal age (< 20 years/≥ 20 years), low maternal education (yes/no; referring to qualified up to certificate of secondary qualification level, vs. qualified to at least vocational level, O-level or A-level), marital status (single mother/with partner), three or more siblings (yes/no) and family income (lowest quintile/second-fifth quintiles). The cumulative number of sociodemographic risk factors was summed, up to five, for each child as has been done previously (Murray et al., 2015).

### Statistical analysis

For the applied example, we contrasted the performance of the same techniques to relate a latent class exposure to a distal outcome including: (i) “one-step” estimation, (ii) “bias-adjusted three-step,” (iii) “modal class assignment,” (iv) “nPCD,” (v) “incPCD,” and (vi) “uPCD.” Again, mediation models were estimated with all six approaches using a SEM framework based on two logistic regression models involving outcome *Y*, exposure *X* (latent classes), and mediator *M* [Equations (1) and (2) above]. Mediation effects (including TE, TNIE, and PNDE) were then derived using direct application of the mediation formula (Muthén, 2011; Pearl, 2012) as described in Supplement 1. Annotated analysis scripts for the applied example using R and M*plus* are available here https://github.com/gemmahammerton/latentclass-mediation.

For incPCD and nPCD we generated 40 imputed datasets, and for uPCD, we generated 60 imputed datasets. The number of imputed datasets for uPCD differed from the simulated data, given that it was chosen to ensure that Monte Carlo errors were no more than 10% of the SE for the parameters in the regression model for *Y* and the regression model for *M* (see Supplement 2). This could reflect the relationship between the latent class indicators and the underlying latent class variable (with stronger auxiliary data meaning that fewer imputed datasets are required (Madley-Dowd et al., 2019). Supplement 3 provides further details on uPCD, including the method used to incorporate baseline confounders.

### Results

**Figure 3.**
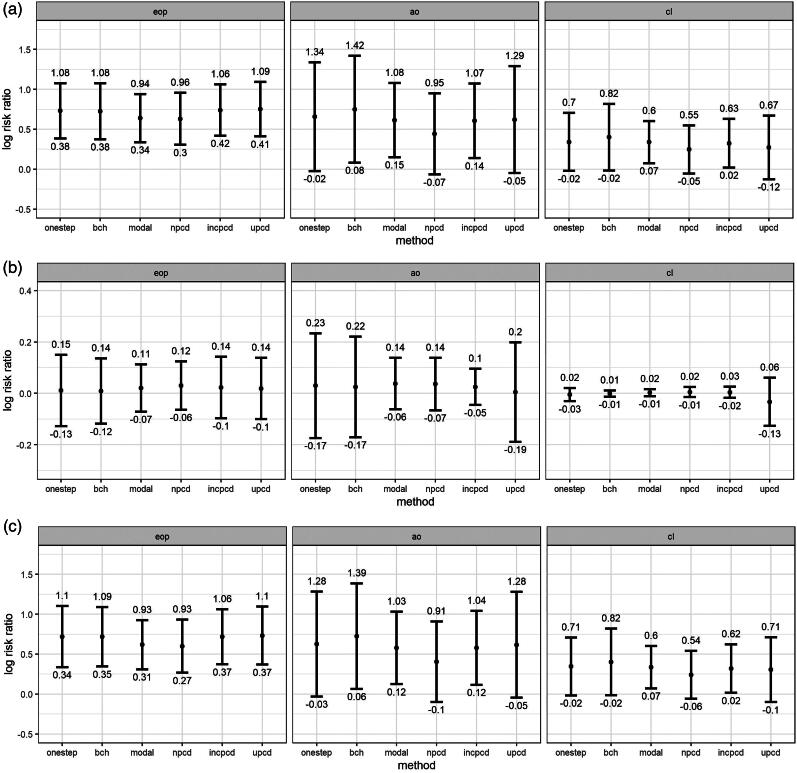
(a) Total effects (TE) for the applied example by method and class comparison; (b) Total natural indirect effects (TNIE) for the applied example by method and class comparison; (c) Pure natural direct effects (PNDE) for the applied example by method and class comparison; Effect estimates shown are log-risk ratios and 95% confidence intervals for each latent class (Early-Onset Persistent, Adolescent Onset, and Childhood Limited) versus the Low class, N = 3,039; Methods = one-step, bias-adjusted threestep (bch), modal class assignment (modal), non-inclusive PCDs (npcd), inclusive PCDs (incpcd), and updated PCDs (upcd); latent class comparisons = Early-Onset Persistent (eop) versus Low, Adolescent Onset (ao) versus Low; Childhood Limited (cl) versus Low.

Figure 3 shows log-risk ratios (and 95% confidence intervals) for the mediation effects by method (one-step, bias-adjusted three-step, modal class assignment, nPCD, incPCD, and uPCD) and latent class comparison (EOP, AO, and CL versus Low conduct problems). Figure 3a shows the TE, Figure 3b shows the TNIE, and Figure 3c shows the PNDE. As shown in the Figures, effect estimates and SE are broadly similar for one-step and uPCD. For the TE and PNDE, effect estimates, and SE are underestimated (compared to one-step) for modal class assignment and nPCD, and SE are underestimated for incPCD. The TNIE is close to zero for all class comparisons, and effect sizes and SE are similar across methods.

## Discussion

Using a limited set of simulations as a proof-of-concept and an applied example utilizing data from a large UK population-based birth cohort (ALSPAC) we have compared mediation effects (and their SE) estimated using a new method (uPCD) to mediation effects estimated using existing methods that are either frequently used in practice (one-step, modal class assignment), currently recommended (bias-adjusted three-step), or no longer used in practice, but closely related to uPCD (nPCD, incPCD). We simulated a latent class exposure, binary mediator, and binary outcome across three levels of latent class separation (high, medium, and low entropy levels). We found that uPCD showed minimal levels of bias across all entropy levels, estimands, and class comparisons, which was comparable to recommended methods (one-step and bias-adjusted three-step). The precision was also similar for uPCD and the one-step method; however, both methods overestimated the model-based SE when estimating the indirect and direct effects in the poor entropy model. This is likely to be a consequence of the complexity of the model, given the sample size and class separation. Additionally, it is only the one-step and uPCD methods that account for the uncertainty in the parameters in the measurement (class derivation) model, when estimating the structural (mediation) model, meaning that uncertainty in the latent class parameters is carried through to the mediation effects. These results support a previous simulation study that found that the one-step method can overestimate parameter uncertainty in conditions with low entropy (Bakk et al., 2013).

In the applied example, we compared mediation effects (and their SE) across all six methods using developmental trajectories of childhood conduct problems as the latent class exposure, illegal drug use as the mediator, and high internalizing symptoms as the outcome. We found that uPCD showed similar results to existing methods that are known to estimate associations between a latent class exposure and binary distal outcome without bias (one-step and bias-adjusted three-step).

### Comparison with existing literature

Our findings for the methods that are no longer recommended (modal class assignment and nPCD) were consistent with previous simulation studies (Bakk et al., 2013; Bray et al., 2015; Vermunt, 2010). The greatest bias was found for nPCD, followed by modal class assignment, with bias toward the null, particularly when the entropy was poor. These methods were also overly precise, with smaller empirical SE compared to other methods, particularly with poor entropy. Even at medium (0.8) and high (0.9) entropy levels and with a reasonably large sample size (*n_ob_*_s_ = 5,000), mediation effects were attenuated with 15% and 19% bias for modal class assignment and nPCD, respectively, when estimating direct effects with medium levels of entropy. The only exception to this was when the effect size was close to zero (which was the case for the TNIE of CL versus Low conduct problems), and all methods showed minimal bias across all entropy levels (as has been shown previously, Bray et al., 2015). This is an important finding, given that many researchers still use a cut point of 0.8 on entropy to justify exporting latent classes and treating them as an observed variable in subsequent analyses. As discussed previously (Bray et al., 2015; Dziak et al., 2016), attenuation in parameter estimates for these methods is due to a mismatch between the class derivation and analysis model, resulting in “omitted outcome” bias which is well-known in the multiple imputation literature (Collins et al., 2001). This bias is addressed through the use of either incPCD or uPCD.

For incPCD, we found minimal bias across nearly all entropy levels, estimands, and class comparisons, which supports previous simulation studies examining the performance of incPCD with a binary distal outcome (Bray et al., 2015; Dziak et al., 2016). However, this method did show bias toward the null for one specific estimand—the TNIE for AO versus Low conduct problems. The bias specifically for the TNIE could be because it was not possible to include the exposure-mediator interaction as a covariate in the class derivation model, meaning that the measurement model was not compatible with the analysis model. This bias may have been present only for the AO class given that this class had the strongest effect size for the TNIE, and also has poorer class separation from the Low class compared to the EOP class. We also found under-coverage for incPCD in the poor entropy model for the TE and PNDE which could be due to underestimated model-based SE. This supports a previous simulation study (Dziak et al., 2016) and may be due to the distal outcomes forming part of the measurement model and this additional model flexibility causing overfitting. The opposite pattern was found for the TNIE, with slight over-coverage in the poor entropy model reflecting the over-estimated model-based SE. Again, this could be due to omitting the exposure-mediator interaction as a covariate in the class derivation model. Although incPCD performs well in certain situations, previous studies have shown that it performs poorly when model assumptions are not met. For example, incPCD assumes homoscedastic normality of the distal outcome (Dziak et al., 2016) and will result in bias when the variances of the distal outcome are not equal across latent classes, unless a quadratic term is included as a covariate in the class derivation model alongside a linear term for the numeric distal outcome (Dziak et al., 2016). There are also limitations associated with a complex class derivation model, such as estimation problems, risk of latent classes being distorted, and lack of transportability of the latent class model across studies (Vermunt, 2010). Additionally, incPCD requires distal outcomes to be treated as covariates in the class derivation model, and the default (at least in M*plus*) is to drop all individuals with missing data on covariates from the analysis model (rather than employing full information maximum likelihood (FIML) to address missing data, as is the default strategy with distal outcomes).

Some of these limitations can be addressed by uPCD, given that it avoids the need for a complex class derivation model that is required for one-step and incPCD. UPCD uses an unconditional latent class model, thereby avoiding the problems of the one-step model such as distorted classes and lack of convergence with more complex models, particularly when sample size decreases (Bakk & Vermunt, 2016). Using an unconditional latent class model also means that the distal outcomes cannot contribute to the definition of the classes which can create a circularity problem with the one-step model (Bakk & Kuha, 2021; Bakk & Vermunt, 2016).

In a simple analysis model (e.g., with a latent class exposure, confounders, and a distal outcome), existing bias-adjusted three-step methods have been shown to perform well and the advantages of using uPCD would not outweigh the disadvantages of the added complexity of the method. However, uPCD has a much greater advantage when the goal is to estimate a more complex model. Bias-adjusted three-step methods can become unwieldy in more complex models (Bakk & Kuha, 2021) and there is the possibility that the distribution of the latent classes can change across analysis models including different external variables which is problematic for any subsequent analysis which involves multiple steps. Additionally, using bias-adjusted three step methods prevents the use of many of the ready-made packages for performing complex methods such as counterfactual mediation (e.g., paramed or gformula in Stata) meaning that any code needs to be manually written and implemented which can be a barrier for applied researchers.

Our new method (uPCD) can address these limitations, as the latent classes can be treated as a manifest nominal variable in any subsequent analyses meaning that a researcher has complete flexibility in which analysis model to use, and does not need to be constrained to methods or packages which allow latent classes to be incorporated (which are currently limited for counterfactual mediation). This also opens up additional software options, which is useful as bias-adjusted three step methods are only currently implemented in programmes such as Mplus and Latent Gold (which both involve subscription costs). Treating the latent classes as a manifest variable also means that researchers do not need to be concerned about the distribution of the latent classes changing across each step of the subsequent analysis. UPCD shares the advantages of methods that use an unconditional class derivation model (e.g., bias-adjusted three-step methods, modal class assignment, nPCD), and also shares the advantages of methods that can treat the latent classes as an observed variable in the analysis model (e.g., modal class assignment, methods using PCD), but without the bias associated with the methods that use both (e.g., modal class assignment, nPCD). Finally, uPCD involves perturbing the parameters from the latent class derivation model which is important to correct SE in the mediation model for the uncertainty in the parameters from the unconditional latent class model. This becomes particularly important as the sample size decreases (Bakk et al., 2013; Vermunt, 2010).

### Limitations of the study

The findings need to be interpreted in the context of several limitations of the study. First, in both the simulation study and applied example, we have focused on a specific scenario with a four-class latent class exposure, binary mediator, and binary outcome and further simulation studies will be needed to test whether uPCD can be used in a broader range of situations. Here we focused on a binary outcome because many disease-related outcomes are binary, and one-step methods usually work better with binary rather than continuous distal outcomes allowing us to compare results across methods. However, an important extension will be to examine how uPCD performs with a numeric mediator or outcome. Additionally, we have not examined how uPCD performs in comparison to other methods when the assumptions of the latent class model are violated (e.g., independence of the indicators conditional on latent class, or independence of the indicators and any external variables conditional on latent class). This is a topic for future research. It is also possible that our conclusions depend on the class sizes for the simulated latent classes, with one large class (70%) and three much smaller classes (12%, 10% and 8%). Researchers often use a criterion of smallest class greater or equal to 5% to aid decisions in the class enumeration step, therefore, it is useful to show how uPCD performs with small class sizes; however, future research could investigate the impact of varying class sizes.

Second, in our simulation study, we used a reasonably large sample size for this type of analysis (*n_ob_*_s_ = 5,000). When we re-ran the simulation study using *n_ob_*_s_ = 2,000 as a sensitivity analysis, results were similar to *n_ob_*_s_ = 5,000, although all methods performed worse with a smaller sample size, as has been shown previously (Bray et al., 2015). A sample size smaller than n_obs_ =2,000 would be feasible (across all methods) with a simpler model (e.g., a regression model with a latent class exposure or outcome); however, it is likely to be problematic for estimating a complex mediation model (particularly using the one-step method on which the simulation is based). The data-generating mechanism used in the simulation included a four-category exposure where two of the categories were rare (10% or less), and a mediation model with an interaction between the four-category exposure and a binary mediator. Model complexity would need to be reduced if only a smaller sample was available (regardless of the method used to relate the latent classes to distal outcomes). Additionally, with a larger sample but very low entropy, only the one-step method is recommended (Bakk et al., 2013; Vermunt, 2010).

Third, there is evidence that PCD results in more bias than modal class assignment when using both the non-inclusive and inclusive approaches (Asparouhov & Muthén, 2014; Bray et al., 2015); however, using modal class assignment rather than PCD was not feasible with our updated method so these approaches could not be compared. There is another alternative to modal class assignment known as proportional assignment, where an individual’s class assignment probabilities are used as regression weights (Bakk & Vermunt, 2016) rather than using them to impute class membership (as in methods based on PCD). Previous simulation studies have shown that when bias-adjusted three step-methods are used with proportional rather than model assignment, parameter estimates are closer to the true value (Bakk et al., 2014; Heron et al., 2015). Future research could investigate whether there is any advantage to combining uPCD with proportional assignment. Fourth, given the time taken to run the analyses, we have not used bootstrapping to estimate SE and confidence intervals for mediation effects in the simulation study or applied example. Various options exist to combine bootstrapping with multiple imputation (Schomaker & Heumann, 2018), or alternatively, the R package *RMediation* (Tofighi & MacKinnon, 2011) could be used to compute the 95% Monte Carlo confidence interval for the mediation effects.

Finally, in our applied example we used FIML in the class derivation model to permit the inclusion of partial respondents on the latent class indicators based on the missing-at-random assumption. Although this allows a larger, more representative starting sample, it can lead to a lower entropy due to additional uncertainty around the incomplete observations for the latent class indicators (Heron et al., 2015). We restricted the analysis sample to those with complete data on the confounders, mediator and outcome, which resulted in a sample size of just over 3,000 (approximately 20% of the original ALSPAC sample) meaning our estimates may not generalize to the original sample enrolled and may also be biased due to selection.

### Challenges of uPCD and recommendations for use

Despite the advantages of uPCD, there are some additional challenges which will need to be investigated in future research. We found that even with a sample size of 5,000, there were occasionally zero cells in the cross-tabulations for the exposure, mediator and outcome within some iterations for simulated datasets with poor entropy, and this became more common when the sample size was reduced to 2,000. Zero cells can result in perfect prediction in the regression model for the outcome or mediator. If perfect prediction is detected, we recommend using firth logistic regression (Heinze & Schemper, 2002) or Bayesian logistic regression instead of standard logistic regression. If there is more than one zero cell, it is important to consider whether the model is too complex given the sample size. One option here would be to reduce complexity, for example, through removing an exposure-mediator interaction. However, if there is an exposure-mediator interaction, and this is not included in the mediation model, indirect effects can be biased (VanderWeele, 2015).

Additionally, for some simulated datasets (particularly those with poor entropy) there was a lot of uncertainty around the within-class probability for certain latent class indicators in the class derivation model. Again, this issue became greater with a smaller sample size for the simulated dataset. Outside of the one-step model, these large SE would not normally affect the analysis model given that the uncertainty in the parameters from the class derivation model is usually not taken into account. However, we found that these large SE could lead to non-convergence for uPCD due to the parameters from the class derivation model being perturbed based on their (co)variance matrix. This non-convergence can be detected by examining a trace plot of parameters and cell sizes across iterations. We chose to exclude simulated datasets when the largest within-class threshold SE from the unconditional latent class model was greater than twice the average of the largest within-class threshold SE across the simulated datasets. If this occurred in an applied example, one option would be to abandon the latent class model and conclude that there is too much uncertainty in the model parameters to conduct subsequent analyses using classes. Alternatively, uPCD could be used but the perturbation for problematic parameters could be somehow controlled, or an alternative method could be considered that doesn’t take into account the uncertainty from the class derivation model. However, taking this uncertainty into account is particularly important when the entropy is low or when the sample size is small (Bakk & Kuha, 2021).

### Future directions and extensions to uPCD approach

In this simulation study offering a proof-of-concept, uPCD performed similarly to recommended methods (one-step and bias-adjusted three-step) to relate a latent class exposure to a binary outcome, and showed minimal bias for mediation effects across various levels of latent class separation. Before uPCD can be adopted by applied researchers to address questions requiring counterfactual mediation with a latent class exposure, it will need to be tested in a broader range of scenarios. Further simulation studies are needed to understand how uPCD performs with a numeric mediator and/or outcome, with multiple mediators or intermediate confounders, with a latent class mediator or outcome, and how it can be combined with multiple imputation for missing data. Further work is also needed to better understand the issues with non-convergence and the specific situations when uPCD should and should not be used.

## Supplementary Material

Supplemental Material

## Data Availability

The M*plus* input files to create the simulated data that support the findings of this study are openly available in Github at https://github.com/gemmahammerton/latentclass-mediation. Access to ALSPAC data (used in the applied example) is through a system of managed open access (http://www.bristol.ac.uk/alspac/researchers/access/).

## Article Information

**Conflict of Interest Disclosures:** Each author signed a form for disclosure of potential conflicts of interest. No authors reported any financial or other conflicts of interest in relation to the work described.

**Ethical Principles:** The authors affirm having followed professional ethical guidelines in preparing this work. These guidelines include obtaining informed consent from human participants, maintaining ethical treatment and respect for the rights of human or animal participants, and ensuring the privacy of participants and their data, such as ensuring that individual participants cannot be identified in reported results or from publicly available original or archival data.

**Funding:** GH is supported by the National Institute for Health Research (NIHR) Health Protection Research Unit in Behavioural Science and Evaluation (HPRU BSE) at the University of Bristol, in partnership with the UK Health Security Agency (UK HSA). This research was also funded by a Sir Henry Wellcome Postdoctoral Fellowship (209138/Z/17/Z) awarded to GH. GH, JH, and KT are members of the MRC Integrative Epidemiology Unit at the University of Bristol (MC_UU_00011/7). This research was funded in whole, or in part, by the Wellcome Trust (209138/Z/17/Z;220488/Z/20/Z).

**Role of the Funders/Sponsors:** None of the funders or sponsors of this research had any role in the design and conduct of the study; collection, management, analysis, and interpretation of data; preparation, review, or approval of the manuscript; or decision to submit the manuscript for publication.

**Acknowledgments:** We are extremely grateful to all the families who took part in ALSPAC, the midwives for their help in recruiting them, and the whole ALSPAC team, which includes interviewers, computer and laboratory technicians, clerical workers, research scientists, volunteers, managers, receptionists and nurses. This publication is the work of the authors and GH will serve as guarantor for the contents of this paper. The UK Medical Research Council (MRC) and Wellcome (Grant Ref: 217065/Z/19/Z) and the University of Bristol provide core support for ALSPAC. A comprehensive list of grants funding is available on the ALSPAC website (http://www.bristol.ac.uk/alspac/external/documents/grant-acknowledgements.pdf). This research was specifically funded by Wellcome Trust (08426812/Z/07/Z) and MRC (G0800612/86812). GH would like to acknowledge support from the National Institute for Health Research (NIHR) Health Protection Research Unit in Behavioural Science and Evaluation (HPRU BSE) at the University of Bristol. The views expressed are those of the authors and not necessarily those of the NIHR, the Department of Health and Social Care or UK Health Security Agency (UKHSA).

## Open Scholarship

This article has earned the Center for Open Science badges for Open Materials. The materials are openly accessible at https://github.com/gemmahammerton/latentclass-mediation.

## References

[CIT0001] Asparouhov, T., & Muthén, B. (2014). Auxiliary variables in mixture modeling: Three-step approaches using Mplus. *Structural Equation Modeling: A Multidisciplinary Journal*, *21*(3), 329–341. 10.1080/10705511.2014.915181

[CIT0002] Babor, T. F., Higgins-Biddle, J. C., Saunders, J. B., & Monteiro, M. G. (2001). *The alcohol use disorders identification test*. World Health Organisation.

[CIT0003] Bakk, Z., & Kuha, J. (2021). Relating latent class membership to external variables: An overview. *The British Journal of Mathematical and Statistical Psychology*, *74*(2), 340–362. 10.1111/bmsp.12227 33200411 PMC8247311

[CIT0004] Bakk, Z., Oberski, D. L., & Vermunt, J. K. (2014). Relating latent class assignments to external variables: Standard errors for correct inference. *Political Analysis*, *22*(4), 520–540. 10.1093/pan/mpu003

[CIT0005] Bakk, Z., Tekle, F. B., & Vermunt, J. K. (2013). Estimating the association between latent class membership and external variables using bias-adjusted three-step approaches. *Sociological Methodology*, *43*(1), 272–311. 10.1177/0081175012470644

[CIT0006] Bakk, Z., & Vermunt, J. K. (2016). Robustness of stepwise latent class modeling with continuous distal outcomes. *Structural Equation Modeling: A Multidisciplinary Journal*, *23*(1), 20–31. 10.1080/10705511.2014.955104

[CIT0007] Bandeen-Roche, K., Miglioretti, D. L., Zeger, S. L., & Rathouz, P. J. (1997). Latent variable regression for multiple discrete outcomes. *Journal of the American Statistical Association*, *92*(440), 1375–1386. 10.1080/01621459.1997.10473658

[CIT0008] Barker, E. D., & Maughan, B. (2009). Differentiating early-onset persistent versus childhood-limited conduct problem youth. *The American Journal of Psychiatry*, *166*(8), 900–908. 10.1176/appi.ajp.2009.08121770 19570930

[CIT0009] Baskin-Sommers, A. R., & Baskin, D. (2016). Psychopathic traits mediate the relationship between exposure to violence and violent juvenile offending. *Journal of Psychopathology and Behavioral Assessment*, *38*(3), 341–349. 10.1007/s10862-016-9535-0

[CIT0010] Berlin, K. S., Parra, G. R., & Williams, N. A. (2014). An introduction to latent variable mixture modeling (part 2): Longitudinal latent class growth analysis and growth mixture models. *Journal of Pediatric Psychology*, *39*(2), 188–203. 10.1093/jpepsy/jst085 24277770

[CIT0011] Berlin, K. S., Williams, N. A., & Parra, G. R. (2014). An introduction to latent variable mixture modeling (part 1): Overview and cross-sectional latent class and latent profile analyses. *Journal of Pediatric Psychology*, *39*(2), 174–187. 10.1093/jpepsy/jst084 24277769

[CIT0012] Bevilacqua, L., Hale, D., Barker, E. D., & Viner, R. (2018). Conduct problems trajectories and psychosocial outcomes: A systematic review and meta-analysis. *European Child & Adolescent Psychiatry*, *27*(10), 1239–1260. 10.1007/s00787-017-1053-4 28983792

[CIT0013] Bolck, A., Croon, M., & Hagenaars, J. (2004). Estimating latent structure models with categorical variables: One-step versus three-step estimators. *Political Analysis*, *12*(1), 3–27. 10.1093/pan/mph001

[CIT0014] Boyd, A., Golding, J., Macleod, J., Lawlor, D. A., Fraser, A., Henderson, J., Molloy, L., Ness, A., Ring, S., & Davey Smith, G. (2013). Cohort Profile: The ‘Children of the 90s’—the index offspring of the Avon Longitudinal Study of Parents and Children. *International Journal of Epidemiology*, *42*(1), 111–127. 10.1093/ije/dys064 22507743 PMC3600618

[CIT0015] Bray, B. C., Lanza, S. T., & Tan, X. (2015). Eliminating bias in classify-analyze approaches for latent class analysis. *Structural Equation Modeling: A Multidisciplinary Journal*, *22*(1), 1–11. 10.1080/10705511.2014.935265 25614730 PMC4299667

[CIT0016] Collier, Z. K., & Leite, W. L. (2017). A comparison of three-step approaches for auxiliary variables in latent class and latent profile analysis. *Structural Equation Modeling: A Multidisciplinary Journal*, *24*(6), 819–830. 10.1080/10705511.2017.1365304

[CIT0017] Collins, L. M., Schafer, J. L., & Kam, C.-K. (2001). A comparison of inclusive and restrictive stategies in modern missing data procedures. *Psychological Methods*, *6*(4), 330–351. 10.1037/1082-989X.6.4.330 11778676

[CIT0018] Daniel, R. M., Stavola, B. L. D., & Cousens, S. N. (2011). gformula: Estimating causal effects in the presence of time-varying confounding or mediation using the g-computation formula. *The Stata Journal: Promoting Communications on Statistics and Stata*, *11*(4), 479–517. 10.1177/1536867X1201100401

[CIT0019] De Stavola, B. L., Daniel, R. M., Ploubidis, G. B., & Micali, N. (2015). Mediation analysis with intermediate confounding: Structural equation modeling viewed through the causal inference lens. *American Journal of Epidemiology*, *181*(1), 64–80. 10.1093/aje/kwu239 25504026 PMC4383385

[CIT0020] Dziak, J. J., Bray, B. C., Zhang, J., Zhang, M., & Lanza, S. T. (2016). Comparing the performance of improved classify-analyze approaches for distal outcomes in latent profile analysis. *Methodology: European Journal of Research Methods for the Behavioral & Social Sciences*, *12*(4), 107–116. 10.1027/1614-2241/a000114 28630602 PMC5473653

[CIT0021] Fraser, A., Macdonald-Wallis, C., Tilling, K., Boyd, A., Golding, J., Davey Smith, G., Henderson, J., Macleod, J., Molloy, L., Ness, A., Ring, S., Nelson, S. M., & Lawlor, D. A. (2013). Cohort profile: The Avon longitudinal study of parents and children: ALSPAC mothers cohort. *International Journal of Epidemiology*, *42*(1), 97–110. 10.1093/ije/dys066 22507742 PMC3600619

[CIT0022] Gasparini, A. (2018). rsimsum: Summarise results from Monte Carlo simulation studies. *Journal of Open Source Software*, *3*(26), 739. 10.21105/joss.00739

[CIT0023] Goodman, R. (2001). Psychometric properties of the strengths and difficulties questionnaire. *Journal of the American Academy of Child and Adolescent Psychiatry*, *40*(11), 1337–1345. 10.1097/00004583-200111000-00015 11699809

[CIT0024] Hallquist, M. N., & Wiley, J. F. (2018). MplusAutomation: An R package for facilitating large-scale latent variable analyses in Mplus. *Structural Equation Modeling: A Multidisciplinary Journal*, *25*(4), 621–638. 10.1080/10705511.2017.1402334 30083048 PMC6075832

[CIT0025] Heinze, G., & Schemper, M. (2002). A solution to the problem of separation in logistic regression. *Statistics in Medicine*, *21*(16), 2409–2419. 10.1002/sim.1047 12210625

[CIT0026] Heron, J., Croudace, T., Barker, E., & Tilling, K, University of Bristol. (2015). A comparison of approaches for assessing covariate effects in latent class analysis. *Longitudinal and Life Course Studies*, *6*(4), 420–434. 10.14301/llcs.v6i4.322

[CIT0027] Hsiao, Y. Y., Kruger, E. S., Lee Van Horn, M., Tofighi, D., MacKinnon, D. P., & Witkiewitz, K. (2021). Latent class mediation: A comparison of six approaches. *Multivariate Behavioral Research*, *56*(4), 543–557. 10.1080/00273171.2020.1771674 32525404 PMC7808339

[CIT0028] Lewis, G., Pelosi, A. J., Araya, R., & Dunn, G. (1992). Measuring psychiatric disorder in the community: A standardized assessment for use by lay interviewers. *Psychological Medicine*, *22*(2), 465–486. 10.1017/s0033291700030415 1615114

[CIT0029] MacKinnon, D. P., Lockwood, C. M., Hoffman, J. M., West, S. G., & Sheets, V. (2002). A comparison of methods to test mediation and other intervening variable effects. *Psychological Methods*, *7*(1), 83–104. 10.1037/1082-989x.7.1.83 11928892 PMC2819363

[CIT0030] Madley-Dowd, P., Hughes, R., Tilling, K., & Heron, J. (2019). The proportion of missing data should not be used to guide decisions on multiple imputation. *Journal of Clinical Epidemiology*, *110*, 63–73. 10.1016/j.jclinepi.2019.02.016 30878639 PMC6547017

[CIT0031] McLarnon, M. J. W., & O’Neill, T. A. (2018). Extensions of auxiliary variable approaches for the investigation of mediation, moderation, and conditional effects in mixture models. *Organizational Research Methods*, *21*(4), 955–982. 10.1177/1094428118770731

[CIT0032] Morris, T. P., White, I. R., & Crowther, M. J. (2019). Using simulation studies to evaluate statistical methods. *Statistics in Medicine*, *38*(11), 2074–2102. 10.1002/sim.8086 30652356 PMC6492164

[CIT0033] Murphy, S., Shevlin, M., Armour, C., Elklit, A., & Christoffersen, M. N. (2014). Childhood adversity and PTSD experiences: Testing a multiple mediator model. *Traumatology*, *20*(3), 225–231. 10.1037/h0099838

[CIT0034] Murray, J., Maughan, B., Menezes, A. M. B., Hickman, M., MacLeod, J., Matijasevich, A., Gonçalves, H., Anselmi, L., Gallo, E. A. G., & Barros, F. C. (2015). Perinatal and sociodemographic factors at birth predicting conduct problems and violence to age 18 years: Comparison of Brazilian and British birth cohorts. *Journal of Child Psychology and Psychiatry, and Allied Disciplines*, *56*(8), 914–922. 10.1111/jcpp.12369 25471542 PMC4508966

[CIT0035] Muthén, B. (2011). Applications of causally defined direct and indirect effects in mediation analysis using SEM in Mplus.

[CIT0036] Muthén, B., & Asparouhov, T. (2015). Causal effects in mediation modeling: An introduction with applications to latent variables. *Structural Equation Modeling: A Multidisciplinary Journal*, *22*(1), 12–23. 10.1080/10705511.2014.935843

[CIT0037] Muthén, M., & Muthén, B. O. (2017). Mplus Software (Version 8.4).

[CIT0038] Muthén, B. O., Muthén, L. K., & Asparouhov, T. (2017). *Regression and mediation analysis using Mplus*. Muthén & Muthén.

[CIT0039] Nylund-Gibson, K., Grimm, R. P., & Masyn, K. E. (2019). Prediction from latent classes: A demonstration of different approaches to include distal outcomes in mixture models. *Structural Equation Modeling: A Multidisciplinary Journal*, *26*(6), 967–985. 10.1080/10705511.2019.1590146

[CIT0040] Pearl, J. (2012). The causal mediation formula—a guide to the assessment of pathways and mechanisms. *Prevention Science: The Official Journal of the Society for Prevention Research*, *13*(4), 426–436. 10.1007/s11121-011-0270-1 22419385

[CIT0041] R Core Team. (2021). R: A language and environment for statistical computing.

[CIT0042] Ramaswamy, V., Desarbo, W. S., Reibstein, D. J., & Robinson, W. T. (1993). An empirical pooling approach for estimating marketing mix elasticities with PIMS data. *Marketing Science*, *12*(1), 103–124. 10.1287/mksc.12.1.103

[CIT0043] Rubin, D. B. (1987). *Multiple imputation for nonresponse in surveys*. John Wiley & Sons, Inc.

[CIT0044] Sacks, R. M., Takemoto, E., Andrea, S., Dieckmann, N. F., Bauer, K. W., & Boone-Heinonen, J. (2017). Childhood maltreatment and BMI trajectory: The mediating role of depression. *American Journal of Preventive Medicine*, *53*(5), 625–633. 10.1016/j.amepre.2017.07.007 28928037 PMC5679065

[CIT0045] Schomaker, M., & Heumann, C. (2018). Bootstrap inference when using multiple imputation. *Statistics in Medicine*, *37*(14), 2252–2266. 10.1002/sim.7654 29682776 PMC5986623

[CIT0046] Sher, K. J., Jackson, K. M., & Steinley, D. (2011). Alcohol use trajectories and the ubiquitous cat’s cradle: Cause for concern? *Journal of Abnormal Psychology*, *120*(2), 322–335. 10.1037/a0021813 21319874 PMC3091989

[CIT0047] Tofighi, D., & MacKinnon, D. P. (2011). RMediation: An R package for mediation analysis confidence intervals. *Behavior Research Methods*, *43*(3), 692–700. 10.3758/s13428-011-0076-x 21487904 PMC3233842

[CIT0048] Valeri, L., & VanderWeele, T. J. (2013). Mediation analysis allowing for exposure–mediator interactions and causal interpretation: Theoretical assumptions and implementation with SAS and SPSS macros. *Psychological Methods*, *18*(2), 137–150. 10.1037/a0031034 23379553 PMC3659198

[CIT0049] VanderWeele, T. J. (2015). *Explanation in causal inference: Methods for mediation and interaction*. Oxford University Press.

[CIT0050] VanderWeele, T. J. (2016). Mediation analysis: A practitioner’s guide. *Annual Review of Public Health*, *37*(1), 17–32. 10.1146/annurev-publhealth-032315-021402 26653405

[CIT0051] VanderWeele, T., & Vansteelandt, S. (2014). Mediation analysis with multiple mediators. *Epidemiologic Methods*, *2*(1), 95–115. 10.1515/em-2012-0010 25580377 PMC4287269

[CIT0052] VanderWeele, T. J., Vansteelandt, S., & Robins, J. M. (2014). Effect decomposition in the presence of an exposure-induced mediator-outcome confounder. *Epidemiology (Cambridge, Mass.)*, *25*(2), 300–306. 10.1097/EDE.0000000000000034 24487213 PMC4214081

[CIT0053] Vanderweele, T. J., & Vansteelandt, S. (2009). Conceptual issues concerning mediation, interventions and composition. *Statistics and Its Interface*, *2*(4), 457–468. 10.4310/SII.2009.v2.n4.a7

[CIT0054] Vermunt, J. K. (2010). Latent class modeling with covariates: Two improved three-step Approaches. *Political Analysis*, *18*(4), 450–469. 10.1093/pan/mpq025

[CIT0055] Wang, C. P., Hendricks Brown, C., & Bandeen-Roche, K. (2005). Residual diagnostics for growth mixture models: Examining the impact of a preventive intervention on multiple trajectories of aggressive behavior. *Journal of the American Statistical Association*, *100*(471), 1054–1076. 10.1198/016214505000000501

[CIT0056] White, I. R., Royston, P., & Wood, A. M. (2011). Multiple imputation using chained equations: Issues and guidance for practice. *Statistics in Medicine*, *30*(4), 377–399. 10.1002/sim.4067 21225900

[CIT0057] World Health Organisation. (1993). The ICD-10 classification of mental and behavioural disorders.

