# Supplementary material for: Counterfactual Mediation Analysis with a Latent Class Exposure
Source: Multivariate Behav Res. 2024 May 31;59(4):818–40. doi: 10.1080/00273171.2024.2335394 (PMC11286213; doi:10.1080/00273171.2024.2335394)
Supplement: Supplemental Material [file HMBR_A_2335394_SM3655.zip › Latent_classes_and_counterfactual_mediation_supplementary_revised.docx]

Supplementary files

[Supplementary Table 1 - Classification probabilities for the most likely latent class membership, *W* (column) by latent class, *X* (row), taken from a single, large, simulated dataset (*n_ob_*_s_ = 1,000,000) with poor (0.7), medium (0.8), and good entropy (0.9) 2](#_Toc152167798)

[Supplementary Figure 1 - Trajectory shapes and entropy; Early-Onset Persistent (8%), Adolescent Onset (10%), Childhood Limited (12%), Low (70%) 3](#_Toc152167799)

[Supplementary Figure 2 – Path diagrams representing i) one-step estimation, ii) bias-adjusted three-step, iii) modal class assignment, iv) non-inclusive PCD, v) inclusive PCD, and vi) updated PCD 4](#_Toc152167800)

[Supplement 1 - Estimating counterfactual mediation effects using the mediation formula 7](#_Toc152167801)

[Supplement 2 - Exploring the number of imputed datasets needed for uPCD, nPCD and incPCD in the simulated data and applied example 11](#_Toc152167802)

[Supplement 3 - Details on the uPCD approach 14](#_Toc152167803)

[Supplement 4 - Comparison of estimates and standard errors for simulated datasets that were and weren’t excluded based on convergence criteria 25](#_Toc152167804)

[Supplement 5 - Bias in mediation effects by method, data generating mechanism, and latent class comparison before exclusions 35](#_Toc152167805)

[Supplementary Table 2 - Performance measures (Monte Carlo standard errors) for each method and data generating mechanism for the total effect (TE) of Adolescent Onset versus Low conduct problems (true value = 0.251) 38](#_Toc152167806)

[Supplementary Table 3 - Performance measures (Monte Carlo standard errors) for each method and data generating mechanism for the total effect (TE) of Childhood Limited versus Low conduct problems (true value = 0.134) 39](#_Toc152167807)

[Supplement 6 - Performance measures (Monte Carlo standard errors) for each method and data generating mechanism using a reduced sample size for the simulation of *n_ob_*_s_ = 2,000 41](#_Toc152167808)

[Supplementary References 52](#_Toc152167809)

# **Supplementary Table 1** - Classification probabilities for the most likely latent class membership, *W* (column) by latent class, *X* (row), taken from a single, large, simulated dataset (*n_ob_*_s_ = 1,000,000) with poor (0.7), medium (0.8), and good entropy (0.9)

|  | EOP | AO | CL | Low |
| --- | --- | --- | --- | --- |
| Good entropy |  |  |  |  |
| EOP | 0.929 | 0.012 | 0.058 | 0.002 |
| AO | 0.066 | 0.825 | 0.004 | 0.105 |
| CL | 0.037 | 0.000 | 0.895 | 0.067 |
| Low | 0.001 | 0.006 | 0.013 | 0.980 |
| Medium entropy |  |  |  |  |
| EOP | 0.776 | 0.073 | 0.128 | 0.024 |
| AO | 0.082 | 0.690 | 0.013 | 0.215 |
| CL | 0.044 | 0.007 | 0.737 | 0.213 |
| Low | 0.001 | 0.017 | 0.020 | 0.962 |
| Poor entropy |  |  |  |  |
| EOP | 0.682 | 0.121 | 0.131 | 0.066 |
| AO | 0.087 | 0.556 | 0.020 | 0.337 |
| CL | 0.080 | 0.016 | 0.572 | 0.333 |
| Low | 0.002 | 0.029 | 0.031 | 0.938 |

EOP: Early Onset Persistent (8%); AO: Adolescent Onset (10%); CL: Childhood Limited (12%); Low (70%); referred to as D matrix (Vermunt, 2010)

# **Supplementary Figure 1** - Trajectory shapes and entropy; Early-Onset Persistent (8%), Adolescent Onset (10%), Childhood Limited (12%), Low (70%)

(i) Good entropy (0.90)

(ii) Medium entropy (0.80)

(iii) Poor entropy (0.70)

# **[Supplementary Figure 2](#_Toc130985768)** [– Path diagrams representing i) one-step estimation, ii) bias-adjusted three-step, iii) modal class assignment, iv) non-inclusive PCD, v) inclusive PCD, and vi) updated PCD](#_Toc130985768)

i) one-step estimation; the relationships between the latent class variable, *X*, and the observed latent class indicators, ***U*** , as well as the class-specific distal outcome distributions for the mediator, *M*, and outcome, *Y* are estimated simultaneously


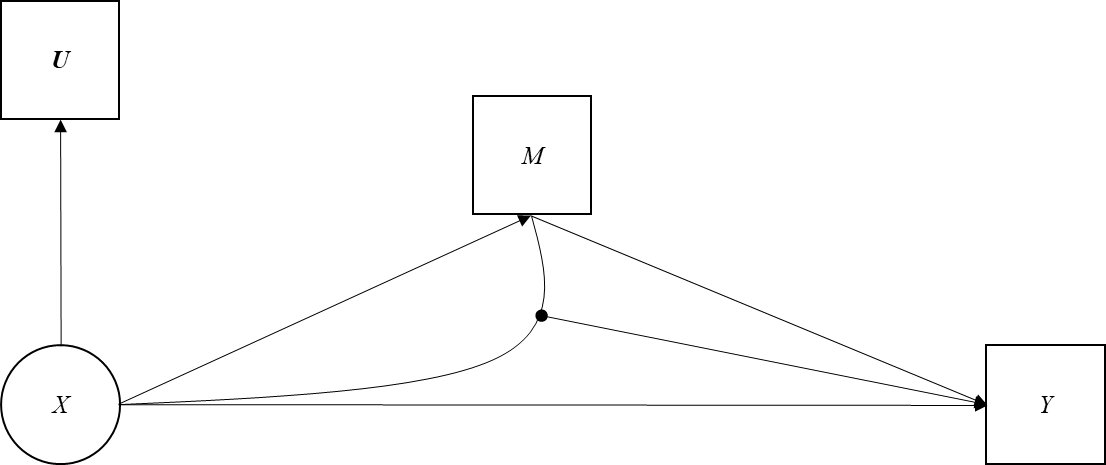


ii) bias-adjusted three-step; in step 1 an unconditional LCA is performed, in step 2 participants are assigned to their most likely class using the class assignment probabilities, $P\left( X=x \right| U)$, to create the nominal observed variable *W,* in step 3 the mediation model is estimated using the nominal variable, *W,* but allowing for the misclassification error introduced in step 2


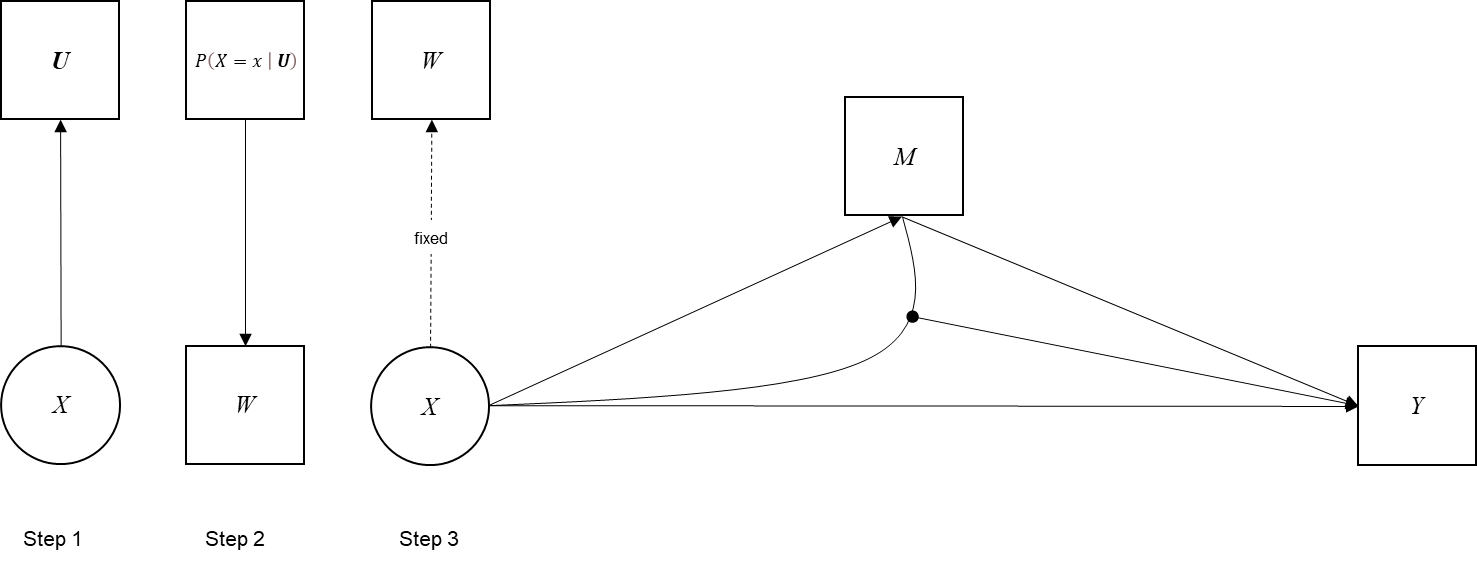


iii) modal class assignment; in step 1 an unconditional LCA is performed, in step 2 participants are assigned to their most likely class using the class assignment probabilities, $P\left( X=x \right| U)$, to create the nominal observed variable *W,* in step 3 the mediation model is estimated using the nominal variable, *W,* not allowing for the misclassification error introduced in step 2


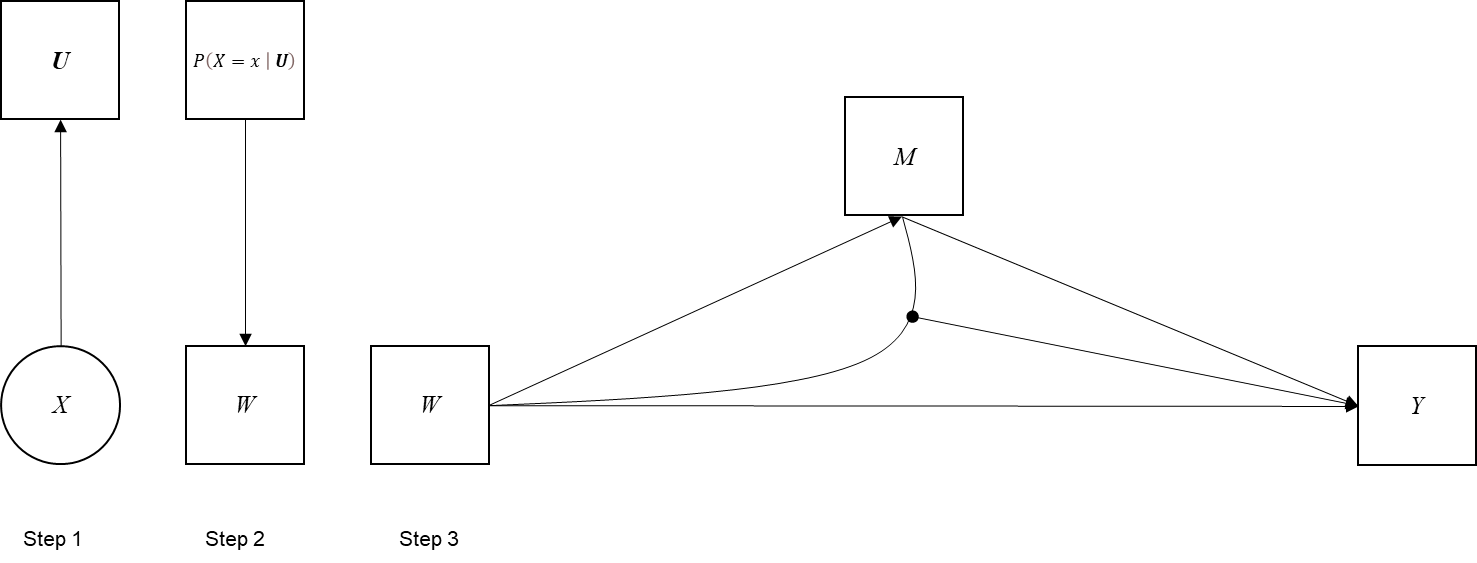


iv) non-inclusive PCD; in step 1 an unconditional LCA is performed, in step 2 the class assignment probabilities, $P\left( X=x \right| U)$, are used to randomly assign each participant to a class *W* = 1, …, *k*, 40 times, in step 3 the mediation model is estimated within each imputed dataset and results are pooled using Rubin’s rules for multiple imputation


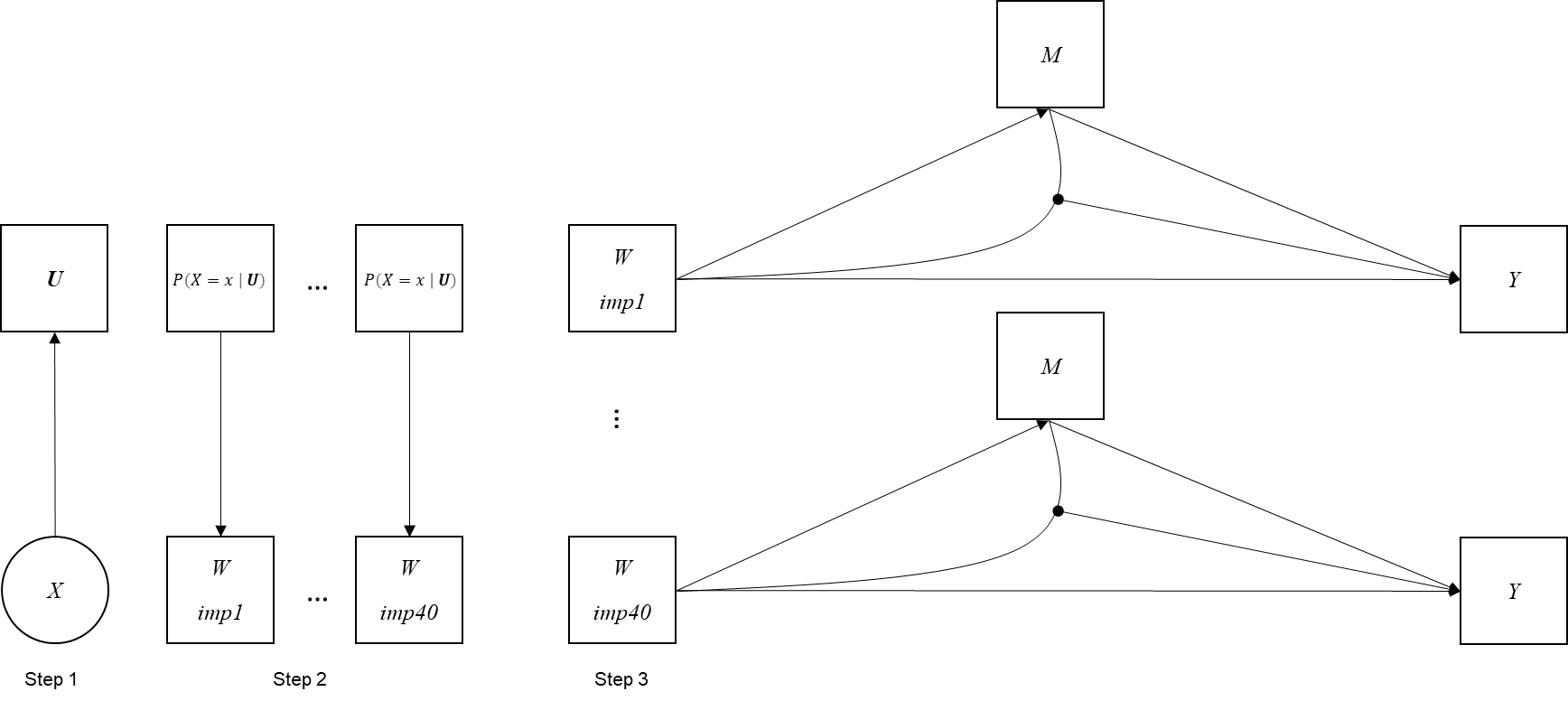


v) inclusive PCD; in step 1 a conditional LCA is performed with the binary mediator *M* and outcome *Y* as covariates predicting latent class membership, in step 2 the class assignment probabilities, $P\left( X=x | Y=y, M=m, U \right)$, are used to randomly assign each participant to a class *W* = 1, …, *k*, 40 times, in step 3 the mediation model is estimated within each imputed dataset and results are pooled using Rubin’s rules for multiple imputation


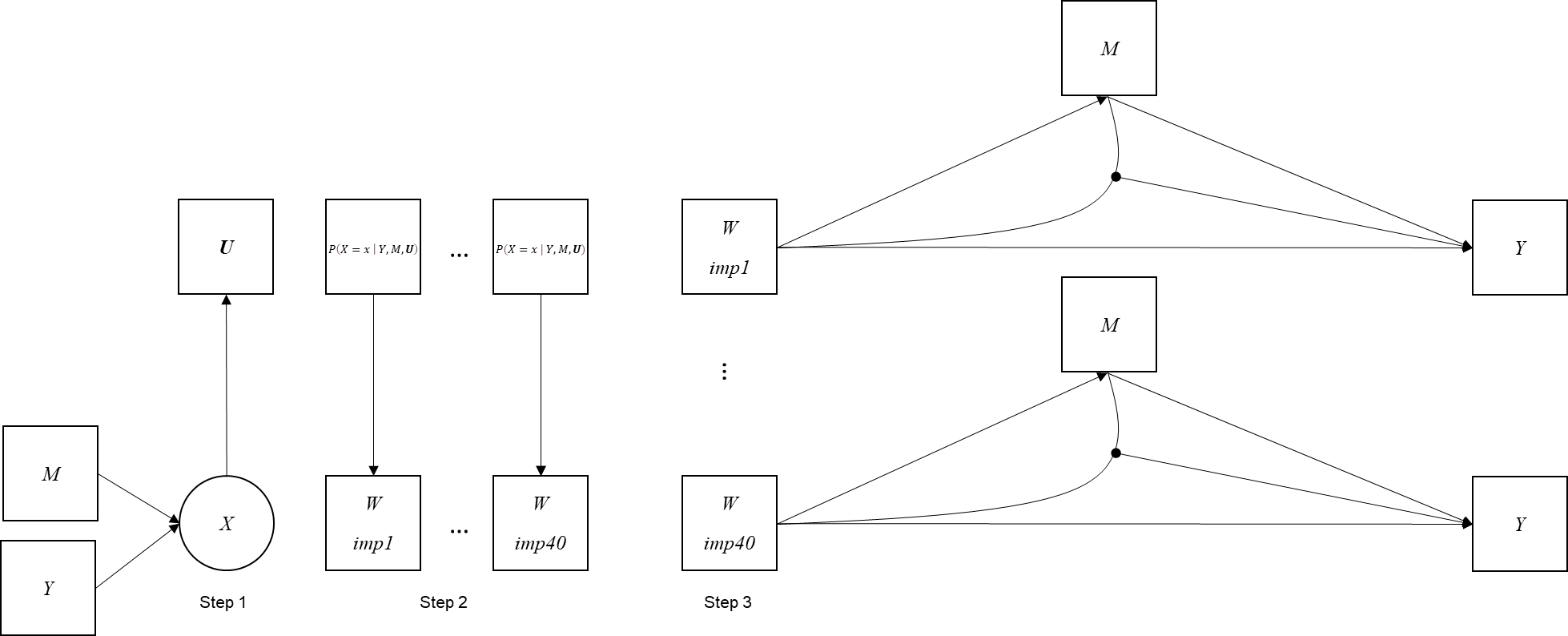


vi) updated PCD; in step 1 an unconditional LCA is performed, in step 2 the class assignment probabilities, $P\left( X=x | Y=y, M=m, U \right)$, are derived manually and used to randomly assign each participant to a class *W* = 1, …, *k*, 80 times, in step 3 the mediation model is estimated within each imputed dataset and results are pooled using Rubin’s rules for multiple imputation


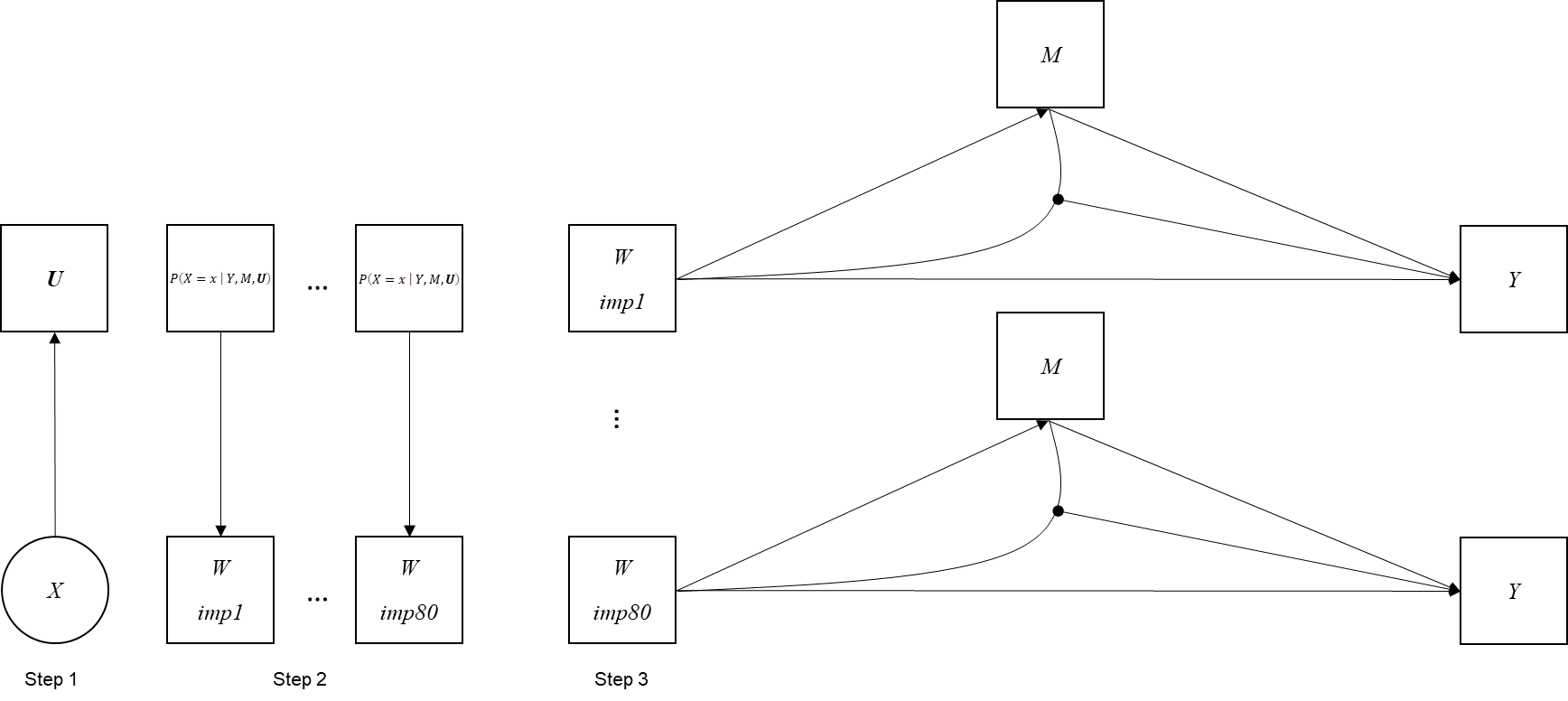


# **Supplement 1** - Estimating counterfactual mediation effects using the mediation formula

In mediation analyses, four assumptions are made with respect to confounding. These include no unmeasured confounders for any of the paths (exposure-outcome, exposure-mediator and mediator-outcome) and no measured or unmeasured confounders for the association between mediator and outcome which lie on the causal pathway from the exposure. For simplicity in the simulation study, we make the unrealistic assumption that there are no confounders, measured or unmeasured, for any path in the model. In the applied example, we assume that the same set of factors (sex and sociodemographic risk factors) confound all paths, and these were assessed before the assessment of the exposure.

In both the simulated and applied example, we assume the presence of an exposure-mediator interaction. This can be thought of as the situation where the effect of *M* on *Y* varies across strata of *X*. The ability to include an *XM* interaction, is one advantage of using the counterfactual mediation framework. There are differing views on whether this interaction should only be included if there is a strong theoretical rationale, or included as standard, given that, if there is an exposure-mediator interaction, and this is not included in the mediation model, indirect effects can be biased (VanderWeele, 2015). Here, we simulate an *XM* interaction because it is common place within the counterfactual mediation literature and therefore useful to show how uPCD performs with this added complexity in the mediation model.

The counterfactual approach is based on conceptualising ‘potential outcomes’ for each individual [Y(*x*)] that would have been observed if particular conditions were met (i.e. had the exposure *X* been set to the value *x* through some intervention) – regardless of the conditions that were in fact met for each individual (VanderWeele, 2015). Mediation effects were estimated in a structural equation modelling (SEM) framework based on two logistic regression models. Using an SEM framework allows the inclusion of a latent class exposure, which is necessary for the one-step approach. Equation 1 involves binary observed outcome *Y* (e.g., hazardous alcohol use), binary observed mediator *M* (e.g., associating with deviant peers), and nominal latent exposure *X* (development trajectories of conduct problems), where *X_1_, X_1_* and *X_3_* are three dummy variables for the latent exposure *X*:

$$\begin{aligned} P\left( Y=1 | X,M \right)=expit\left( \beta_{0}+\beta_{1}M+\beta_{2}X_{1}+\beta_{3}X_{2}+\beta_{4}X_{3}+\beta_{5}X_{1}M+\beta_{6}X_{2}M+\beta_{7}X_{3}M \right)\#\left( 1 \right) \end{aligned}$$

Equation 2 involves binary observed mediator *M*, and nominal latent exposure *X*:

$$\begin{aligned} P\left( M=1 | X \right)=expit\left( \alpha_{0}+\alpha_{1}X_{1}+\alpha_{2}X_{2}+\alpha_{3}X_{3} \right)\#\left( 2 \right) \end{aligned}$$

Here, we estimated the pure natural direct effect (PNDE) and total natural indirect effect (TNIE). The PNDE-TNIE decomposition is commonly used in the causal-inference literature when assessing direct and indirect effects and it is the decomposition on which most of the software packages for causal mediation analysis have focused. As recommended by Nguyen and colleagues (Nguyen et al., 2021), this decomposition is relevant when we assume that there is a direct effect, and want to investigate whether there is also an indirect effect. For a description of alternative decompositions, and a discussion when each would be relevant see (Nguyen et al., 2021).

The method used to derive these effects has been described in detail elsewhere when using a nominal (or latent class) mediator (Muthén, 2011; Muthén et al., 2017), and when using a latent class mediator or exposure and continuous outcome (McLarnon & O’Neill, 2018). Briefly, the estimated parameters from the logistic regression model for Y and the logistic regression model for M were used to derive the probability of the outcome being present given the exposure and mediator, i.e. P(*Y* = 1 | *X* = *x*, *M* = *m*), and the probability of the mediator being present given the exposure, i.e. P(*M* = 1 | *X* = *x*). Potential outcome probabilities, E[*Y*(*x*, *M*(*x'*))] were then derived using direct application of the mediation formula (B. O. Muthén et al., 2017; Pearl, 2012):

$$\begin{aligned} E\left[ Y\left( 0,M\left( 0 \right) \right) \right]=P\left( Y=1 | X=0,M=0 \right)\times P\left( M=0 | X=0 \right)+ \\ P\left( Y=1 | X=0,M=1 \right)\times P\left( M=1 | X=0 \right)\#\left( 3 \right) \end{aligned}$$

$$\begin{aligned} E\left[ Y\left( 1,M\left( 0 \right) \right) \right]=P\left( Y=1 | X=1,M=0 \right)\times P\left( M=0 | X=0 \right)+ \\ P\left( Y=1 | X=1,M=1 \right)\times P\left( M=1 | X=0 \right)\#\left( 4 \right) \end{aligned}$$

$$\begin{aligned} E\left[ Y\left( 1,M\left( 1 \right) \right) \right]=P\left( Y=1 | X=1,M=0 \right)\times P\left( M=0 | X=1 \right)+ \\ P\left( Y=1 | X=1,M=1 \right)\times P\left( M=1 | X=1 \right)\#\left( 5 \right) \end{aligned}$$

Potential outcome probabilities (shown in equations 3 to 5) can be used to calculate the odds ratio, risk ratio, or risk difference for the PNDE, TNIE and total effect (TE), comparing each latent class to the reference class (e.g., ‘Low’ conduct problems). Here, we present risk ratios. The PNDE (equation 6) is the direct (unmediated) effect of the exposure on the outcome when the mediator takes the value it would take in the absence of the exposure. It is thus modelled as the direct effect of exposure *X* = 1 (e.g. ‘Early-Onset Persistent’ conduct problems) versus the absence of exposure *X* = 0 (e.g. ‘Low’ conduct problems) on outcome *Y* (e.g. hazardous alcohol use) if mediator *m* (e.g. associating with deviant peers) were set to whatever it would be for *X* = 0.

$$\begin{aligned} PNDE={E\left[ Y\left( 1,M\left( 0 \right) \right) \right]}/{E\left[ Y\left( 0,M\left( 0 \right) \right) \right]}\#\left( 6 \right) \end{aligned}$$

The TNIE (equation 7) captures the effect of the exposure on the outcome that operates by changing the mediator. It is thus modelled as the effect on outcome *Y* if the exposure were fixed at *X* = 1 and mediator *m* were changed from the level it would take if *X* = 0 to the level it would take if *X* = 1.

$$\begin{aligned} TNIE={E\left[ Y\left( 1,M\left( 1 \right) \right) \right]}/{E\left[ Y\left( 1,M\left( 0 \right) \right) \right]}\#\left( 7 \right) \end{aligned}$$

On the risk ratio scale, the TE = TNIE × PNDE or:

$$\begin{aligned} TE={E\left[ Y\left( 1,M\left( 1 \right) \right) \right]}/{E\left[ Y\left( 0,M\left( 0 \right) \right) \right]}\#\left( 8 \right) \end{aligned}$$

To enable a model with a latent class exposure, binary mediator and binary outcome to be estimated in M*plus,* it was necessary to specify the observed binary mediator as a latent class variable with no uncertainty with regards to who belonged to each category (e.g., each participant has a 0% probability of belonging to one category of the mediator, and a 100% probability of belonging to the other category). Alternatively, this could have been done using the “knownclass” option in M*plus.*

The counterfactual mediation method described above can be extended to incorporate baseline confounders which would result in the estimation of conditional mediation effects (e.g., for a value of zero on each of the confounders) (Muthén et al., 2017). For the applied example, the conditional mediation effects are for males with no sociodemographic risk factors.

# **Supplement 2** - Exploring the number of imputed datasets needed for uPCD, nPCD and incPCD in the simulated data and applied example

For each method using PCD, we imputed up to 500 datasets and estimated the regression model for *Y* (equation 1) and the regression model for *M* (equation 2) using the *mice* package. To limit the impact of uncertainty due to the stochastic nature of the analysis, we calculated the Monte Carlo error for each parameter in the regression models (defined as their standard deviation across repeated runs of the same imputation procedure with the same data; (White et al., 2011)) by taking the between imputation variance *B* divided by the number of imputed datasets *m*, square rooted e.g., $\sqrt{B}/m$. We then calculated the percentage of the standard error (SE) for the same regression parameter, e.g., (Monte Carlo error / SE) × 100. In the tables below, we highlight the number of imputed datasets required to ensure that the Monte Carlo error was no more than 10% of the SE for any parameter in the regression model for *Y* or *M* (White et al., 2011). We also report the total effects of each latent class (Early-Onset Persistent, Adolescent Onset, and Childhood Limited) versus the Low class on the outcome with increasing numbers of imputed datasets. The results show that the total effects stabilise as the number of imputed datasets increase. Although we have 100% missing data for the latent classes, we also have very strong auxiliary data (the latent class indicators) which also impacts on the number of imputed datasets required (Madley-Dowd et al., 2019).

Supplement 2 Table 1 shows the results for the first simulated dataset with poor entropy. The Monte Carlo error was no more than 10% of the SE for any of the parameters in the regression models when we generated 80 imputed datasets for uPCD, and when we generated 40 imputed datasets for nPCD and incPCD. It is likely that fewer imputed datasets would be needed for models with medium and good entropy (Bray et al., 2015); however, we chose to base our decisions on the model with poor entropy to provide a ‘worse-case scenario’ and to allow us to use a consistent number of imputed datasets across all three data-generating mechanisms. Supplement 2 Table 2 shows the results for the applied example. The Monte Carlo error was no more than 10% of the SE for any of the parameters in the regression models when we generated 60 imputed datasets for uPCD, and when we generated 40 imputed datasets for nPCD and incPCD.

**Supplement 2 Table 1** - Total effects for the **first simulated dataset** with poor entropy using different pseudo class draws (PCD) methods with differing numbers of imputed datasets; Effect estimates shown are log-risk ratios (standard errors) for the total effect of each latent class (Early-Onset Persistent, Adolescent Onset, and Childhood Limited) versus the Low class on hazardous alcohol use; *N* = 5,000

|  | imputed datasets | Low | Childhood Limited | Adolescent Onset | Early-Onset Persistent | (Monte Carlo error/SE)  × 100 |
| --- | --- | --- | --- | --- | --- | --- |
| Updated PCD | 5 | Reference | 0.16 (0.12) | 0.41 (0.08) | 0.42 (0.06) | ≤37% |
|  | 10 | Reference | 0.13 (0.10) | 0.38 (0.10) | 0.43 (0.08) | ≤29% |
|  | 20 | Reference | 0.10 (0.11) | 0.37 (0.10) | 0.43 (0.07) | ≤21% |
|  | 40 | Reference | 0.10 (0.10) | 0.38 (0.10) | 0.43 (0.08) | ≤15% |
|  | 60 | Reference | 0.11 (0.10) | 0.39 (0.11) | 0.43 (0.07) | ≤12% |
|  | 80 | Reference | 0.11 (0.11) | 0.40 (0.10) | 0.42 (0.08) | ≤10% |
|  | 100 | Reference | 0.11 (0.10) | 0.39 (0.10) | 0.42 (0.08) | ≤9% |
|  | 250 | Reference | 0.12 (0.10) | 0.39 (0.10) | 0.42 (0.08) | ≤6% |
|  | 500 | Reference | 0.12 (0.10) | 0.39 (0.10) | 0.42 (0.08) | ≤4% |
| Non-inclusive PCD | 5 | Reference | 0.06 (0.07) | 0.16 (0.09) | 0.31 (0.05) | ≤33% |
|  | 10 | Reference | 0.08 (0.07) | 0.16 (0.08) | 0.31 (0.06) | ≤21% |
|  | 20 | Reference | 0.09 (0.07) | 0.17 (0.08) | 0.30 (0.06) | ≤14% |
|  | 40 | Reference | 0.08 (0.07) | 0.18 (0.07) | 0.30 (0.06) | ≤10% |
|  | 60 | Reference | 0.08 (0.07) | 0.17 (0.08) | 0.30 (0.06) | ≤9% |
|  | 80 | Reference | 0.08 (0.07) | 0.17 (0.08) | 0.30 (0.06) | ≤8% |
|  | 100 | Reference | 0.08 (0.07) | 0.17 (0.08) | 0.30 (0.06) | ≤7% |
|  | 250 | Reference | 0.08 (0.07) | 0.17 (0.08) | 0.30 (0.06) | ≤4% |
|  | 500 | Reference | 0.08 (0.07) | 0.17 (0.08) | 0.30 (0.06) | ≤3% |
| Inclusive PCD | 5 | Reference | 0.13 (0.08) | 0.35 (0.06) | 0.46 (0.05) | ≤30% |
|  | 10 | Reference | 0.14 (0.07) | 0.36 (0.07) | 0.46 (0.05) | ≤20% |
|  | 20 | Reference | 0.14 (0.07) | 0.34 (0.07) | 0.45 (0.06) | ≤14% |
|  | 40 | Reference | 0.14 (0.07) | 0.33 (0.07) | 0.44 (0.06) | ≤10% |
|  | 60 | Reference | 0.13 (0.07) | 0.33 (0.06) | 0.45 (0.06) | ≤8% |
|  | 80 | Reference | 0.14 (0.07) | 0.33 (0.06) | 0.44 (0.06) | ≤7% |
|  | 100 | Reference | 0.14 (0.07) | 0.33 (0.06) | 0.44 (0.06) | ≤6% |
|  | 250 | Reference | 0.13 (0.07) | 0.33 (0.06) | 0.45 (0.06) | ≤4% |
|  | 500 | Reference | 0.13 (0.07) | 0.33 (0.06) | 0.44 (0.06) | ≤3% |

One-step results: Early-Onset Persistent versus Low class: 0.44 (0.07); Adolescent-Onset versus Low class: 0.34 (0.09); Childhood Limited versus Low class: 0.15 (0.09); SE = standard error

**Supplement 2 Table 2** - Total effects for the **applied example** using different pseudo class draws (PCD) methods with differing numbers of imputed datasets; Effect estimates shown are log-risk ratios (standard errors) for the total effects of each latent class (Early-Onset Persistent, Adolescent Onset, and Childhood Limited) versus the Low class on high internalising symptoms; *N* = 3,039

|  | imputed datasets | Low | Childhood Limited | Adolescent Onset | Early-Onset Persistent | (Monte Carlo error/SE)  × 100 |
| --- | --- | --- | --- | --- | --- | --- |
| Updated PCD | 5 | Reference | 0.32 (0.15) | 0.66 (0.31) | 0.80 (0.18) | ≤29% |
|  | 10 | Reference | 0.32 (0.18) | 0.65 (0.27) | 0.75 (0.19) | ≤22% |
|  | 20 | Reference | 0.31 (0.17) | 0.64 (0.28) | 0.76 (0.18) | ≤16% |
|  | 40 | Reference | 0.28 (0.19) | 0.67 (0.28) | 0.75 (0.18) | ≤12% |
|  | 60 | Reference | 0.27 (0.20) | 0.62 (0.34) | 0.75 (0.17) | ≤10% |
|  | 100 | Reference | 0.25 (0.20) | 0.67 (0.33) | 0.75 (0.17) | ≤8% |
|  | 250 | Reference | 0.25 (0.20) | 0.65 (0.33) | 0.74 (0.17) | ** |
|  | 500 | Reference | 0.25 (0.20) | 0.66 (0.32) | 0.73 (0.17) | ** |
| Non-inclusive PCD | 5 | Reference | 0.29 (0.15) | 0.44 (0.25) | 0.67 (0.16) | ≤23% |
|  | 10 | Reference | 0.28 (0.15) | 0.44 (0.26) | 0.66 (0.16) | ≤18% |
|  | 20 | Reference | 0.27 (0.16) | 0.44 (0.25) | 0.63 (0.17) | ≤14% |
|  | 40 | Reference | 0.25 (0.15) | 0.44 (0.26) | 0.63 (0.17) | ≤10% |
|  | 60 | Reference | 0.25 (0.16) | 0.45 (0.26) | 0.63 (0.16) | ≤8% |
|  | 100 | Reference | 0.24 (0.16) | 0.44 (0.27) | 0.63 (0.17) | ≤6% |
|  | 250 | Reference | 0.22 (0.16) | 0.46 (0.27) | 0.62 (0.17) | ≤4% |
|  | 500 | Reference | 0.22 (0.16) | 0.44 (0.27) | 0.62 (0.17) | ≤3% |
| Inclusive PCD | 5 | Reference | 0.36 (0.17) | 0.65 (0.22) | 0.72 (0.18) | ≤32% |
|  | 10 | Reference | 0.30 (0.16) | 0.60 (0.24) | 0.74 (0.17) | ≤20% |
|  | 20 | Reference | 0.29 (0.15) | 0.60 (0.25) | 0.74 (0.17) | ≤15% |
|  | 40 | Reference | 0.32 (0.16) | 0.60 (0.24) | 0.74 (0.16) | ≤10% |
|  | 60 | Reference | 0.33 (0.15) | 0.63 (0.24) | 0.73 (0.17) | ≤8% |
|  | 100 | Reference | 0.35 (0.15) | 0.62 (0.24) | 0.74 (0.17) | ≤6% |
|  | 250 | Reference | 0.34 (0.16) | 0.61 (0.24) | 0.73 (0.17) | ≤4% |
|  | 500* | Reference | 0.35 (0.16) | 0.62 (0.24) | 0.73 (0.17) | ≤3% |

One-step results: Early-Onset Persistent versus Low class: 0.73 (0.18); Adolescent Onset versus Low class: 0.66 (0.35); Childhood Limited versus Low class: 0.34 (0.19)

*only 499 completed

**regression model for *Y* does not converge due to zero cells in imputed dataset

# **Supplement 3** - Details on the uPCD approach

Let ***U*** be a vector of *p* observed binary manifest variables (“class indicators”), *X* be a nominal latent class variable with categories *x* = 1, …, *k*, *W* be an observed nominal variable generated using an individual’s updated class assignment probabilities, *M* be a dichotomous mediator (coded 0 or 1) and *Y* be a dichotomous outcome (also coded 0 or 1). Interest lies in a model for *Y*: $P\left( Y=1 | X,M \right),$ and a model for *M*: $P\left( M=1 | X \right),$ both indexed by unknown parameters; for example, in the simulated and applied examples (where we used four latent classes) we would express these as per equations 1 and 2 below (also found in Supplement 1), where *X_i_* are *k*-1 dummy variables representing latent *X*, the exposure in the mediation model.

$$\begin{aligned} P\left( Y=1 | X,M \right)=expit\left( \beta_{0}+\beta_{1}M+\beta_{2}X_{1}+\beta_{3}X_{2}+\beta_{4}X_{3}+\beta_{5}X_{1}M+\beta_{6}X_{2}M+\beta_{7}X_{3}M \right)\#\left( 1 \right) \end{aligned}$$

$$\begin{aligned} P\left( M=1 | X \right)=expit\left( \alpha_{0}+\alpha_{1}X_{1}+\alpha_{2}X_{2}+\alpha_{3}X_{3} \right)\#\left( 2 \right) \end{aligned}$$

UPCD involves multiply imputing *X* by drawing it randomly from a model for: $P\left( X | Y,M\boldsymbol{,U} \right),$ then fitting the model for $P\left( Y=1 | W,M \right)$and for $P\left( M=1 | W \right)$and using Rubin's rules to calculate the variance of the resulting estimators of **β** and **α**. Below we provide a detailed breakdown of the steps involved in uPCD.

***Unconditional latent class analysis (LCA)***

First, a measurement model for latent X is estimated based on only ***U*** i.e. an unconditional LCA. Suppose that ***U*** consists of components *U*_1_, …, *U*_p_, which we assume to be mutually independent, conditional on *X*. LCA consists of a structural model and a measurement model. In an unconditional LCA, the structural model relates to the unconditional probability of belonging to latent class *x*, $P\left( X=x \right)$, which is denoted by $\pi_{x}$. The measurement model relates to the class-specific probability of a pattern of responses to the latent class indicators, $P\left( U_{j} | X=x \right)$, where $U_{j}, j=1,\ldots,p$ represents the responses for the latent class indicators.

In most software packages, the class proportions, $\pi_{x}$ , are parameterised as (*k*-1) intercepts on the inverse multinomial scale with $\gamma_{0x}$ = 0 for identification (Nylund-Gibson & Choi, 2018):

$$\begin{aligned} \pi_{x}=P\left( X=x \right)= \frac{\exp(\gamma_{0x})}{\sum_{x*=1}^{k} \exp(\gamma_{0x*})}\#\left( 9 \right) \end{aligned}$$

The class-specific probabilities of the latent class indicators are parameterised as (*p***k*) within-class thresholds, $\tau_{jx}$ , on the inverse logit scale (Nylund-Gibson & Choi, 2018):

$$\begin{aligned} P\left( U_{j}=1 \right|X=x)= \frac{1}{1+exp(\tau_{jx})}\#\left( 10 \right) \end{aligned}$$

Class-assignment probabilities are a function of these two types of probabilities:

$$\begin{aligned} P\left( X=x \right| U_{1}, \ldots U_{p})= \frac{\prod_{j=1}^{p} P\left( U_{j} | X=x \right)P\left( X=x \right)}{\sum_{x*=1}^{k} \prod_{j=1}^{p} P\left( U_{j} | X=x^{*} \right)P\left( X=x^{*} \right)}\#\left( 11 \right) \end{aligned}$$

In the interest of clarity, we have not used *i* to represent the individual in the equations above. All individuals with the same pattern of observed data (observed latent class indicators) have the same within-class probabilities and the same class-assignment probabilities.

The model parameters from a LCA are estimated by maximum likelihood using the expectation-maximization procedure available in most software packages (Nylund-Gibson & Choi, 2018). The procedure is iterative where both parameters (latent class intercepts and within-class thresholds) are estimated to convergence. Once convergence is reached, class assignment probabilities can be derived using the class intercepts and within-class thresholds for different patterns of latent class indicators.

***Deriving updated class assignment probabilities***

Following the estimation of the unconditional LCA, the updated class assignment probabilities, $P\left( X=x | Y, M,U \right)$, need to be calculated for *x* = 1, …,*k.* We make the assumption that ($Y, M$) is independent of $U$ conditional on $X$ (implicit in Figure 1). Using Bayes’ rule, we have that for each *x* = 1, …, *k*,

$$\begin{aligned} P\left( X=x | Y=y, M=m,\boldsymbol{U} \right)= \frac{P\left( X=x | \boldsymbol{U} \right)P\left( Y=y | X=x,M \right)P\left( M=m | X=x \right)}{\sum_{x*} P\left( X=x^{*} | \boldsymbol{U} \right)P\left( Y=y | X=x^{*},M \right)P\left( M=m | X=x^{*} \right)}\#\left( 12 \right) \end{aligned}$$

For given $\boldsymbol{\alpha, \beta},\boldsymbol{\gamma}_{\boldsymbol{0}},\boldsymbol{\tau}$, this can be calculated. Since $\boldsymbol{\alpha, \beta}$ is unknown, the procedure will need to be iterated as we explain next. In doing so, we recommend perturbing the latent class parameters $\boldsymbol{\gamma}_{\boldsymbol{0}},\boldsymbol{\tau}$by jointly perturbing their estimates with Gaussian noise of mean zero and values from the variance-covariance matrix of these parameter estimates. Perturbing the parameters is important to prevent underestimated standard errors which have been shown in simulation studies when the uncertainty in the class assignment probabilities is not taken into account in the final analysis model (Bakk et al., 2013; Vermunt, 2010). Perturbation is also done in the (posterior step of the) data augmentation procedure that underlies multiple imputation software.

***The iterative procedure***

The iterative procedure thus works as follows:

1. Initialise $\boldsymbol{\beta}$by fitting the logistic regression model for *Y* (e.g., equation 1 above) with all $\boldsymbol{\beta}$ except for $\beta_{0}, \beta_{1}$ set to zero and initialise $\boldsymbol{\alpha}$by fitting the logistic regression model for *M* (e.g., equation 2 above) with all $\boldsymbol{\alpha}$ except for $\alpha_{0}$ set to zero. Perturb the estimates of $\boldsymbol{\alpha, \beta}$ with Gaussian noise of mean zero and values from the variance-covariance matrix of these parameter estimates.
2. Perturb the estimates $\boldsymbol{\gamma}_{\boldsymbol{0}},\boldsymbol{\tau}$(latent class intercepts and within-class thresholds from the unconditional LCA) as previously explained. Combine these along with the previous values of $\boldsymbol{\alpha, \beta}$ to calculate probabilities $P\left( X=x | Y,M,\boldsymbol{U} \right)$for *x* = 1, …, *k* for each participant.
3. Use these probabilities to randomly assign each participant to a class *W* = 1, …, *k*. Fit the logistic regression model for $P\left( Y=1 | W,M \right)$and for $P\left( M=1 | W \right)$to obtain updated parameter estimates of$\boldsymbol{\alpha, \beta}$ and perturb with Gaussian noise of mean zero and values from the variance-covariance matrix of these parameter estimates.
4. Repeat steps 2 and 3 until convergence of $\boldsymbol{\alpha, \beta}$ (see details later).
5. Upon convergence, store the last imputed values of *W*. This process can be repeated a number of times and the results combined using Rubin’s rules.

Here we use M*plus* for the unconditional LCA (step 0) because it provides the within-class thresholds, $\tau$, latent class intercepts, $\gamma_{0}$, and the variance-covariance matrix of these parameter estimates; however, any latent class programme can be used as long as it can provide this (co)variance matrix. All subsequent steps were performed using R (version 4.1.1). $\tau$ $\gamma_{0}$Supplement 3 Figure 1 provides a schematic for the procedure.

**Supplement 3 Figure 1** – Schematic for the iterative procedure

0.

Perform unconditional LCA

1.

Perform logistic regression and perturb β_0,_ β_1,_ α_0_

2. Perturb **γ_0_, τ** and combine with **α, β** to calculate class assignment probabilities

3. Perform class assignment and logistic regression, then perturb **α, β**

4.

Repeat 2 and 3 until convergence

5.

Store imputed *W* and repeat the process from step 2

***Additional considerations***

Before imputing class membership using uPCD and performing the subsequent analysis, there are a few decisions that need to be made. First, checks need to be performed to decide on the number of datasets to impute and the number of cycles of iterations needed between saving imputed datasets. These two quantities are just the same as those in multivariate imputation by chained equations.

***Deciding on the number of imputed datasets***

We examined the Monte Carlo error for the parameters in the regression model for *Y* and the regression model for *M* for the first simulated dataset with poor entropy and for the applied example. We identified the number of imputed datasets required to ensure that the Monte Carlo error was no more than 10% of the standard error for any of the parameters in the regression models. For the simulation study we generated 80 imputed datasets (Supplement 2 Table 1), and for the applied example, we generated 60 imputed datasets (Supplement 2 Table 2).

***Deciding on the cycles of iterations between saving imputed values of W***

We used autocorrelation function (ACF) plots to decide on the appropriate number of cycles of iterations. First, we used a traceplot of parameter estimates across iterations to decide on an appropriate burn in (here we use 100). These iterations are then removed before producing the ACF plot. To produce an ACF plot, the function *acf* was used to plot each parameter from the regression model for *Y* and the regression model for *M* across the remaining iterations, with a maximum lag of 50. Supplement 3 Figure 2 shows an example of an ACF plot for the beta coefficient for Early-Onset Persistent versus Low conduct problems in the regression model for *Y* using the first simulated dataset with poor entropy. We use a burn in of 100 iterations (omitted from ACF plots), and 20 cycles of iterations (between saving each of the 80 imputed values of *W*), resulting in 1,700 iterations in total.

**Supplement 3 Figure 2** – Autocorrelation function (ACF) plot for the beta coefficient for Early-Onset Persistent versus Low conduct problems in the regression model for *Y* estimated across 1,700 iterations (with a maximum lag of 50) in the first simulated dataset with poor entropy; x-axis shows the lag for each autocorrelation estimate; y-axis shows the autocorrelation estimates (indicated by the height of the vertical bars); blue horizontal dashed lines show 95% confidence intervals around the autocorrelation estimate at each lag, with a null value of zero representing no autocorrelation at that lag


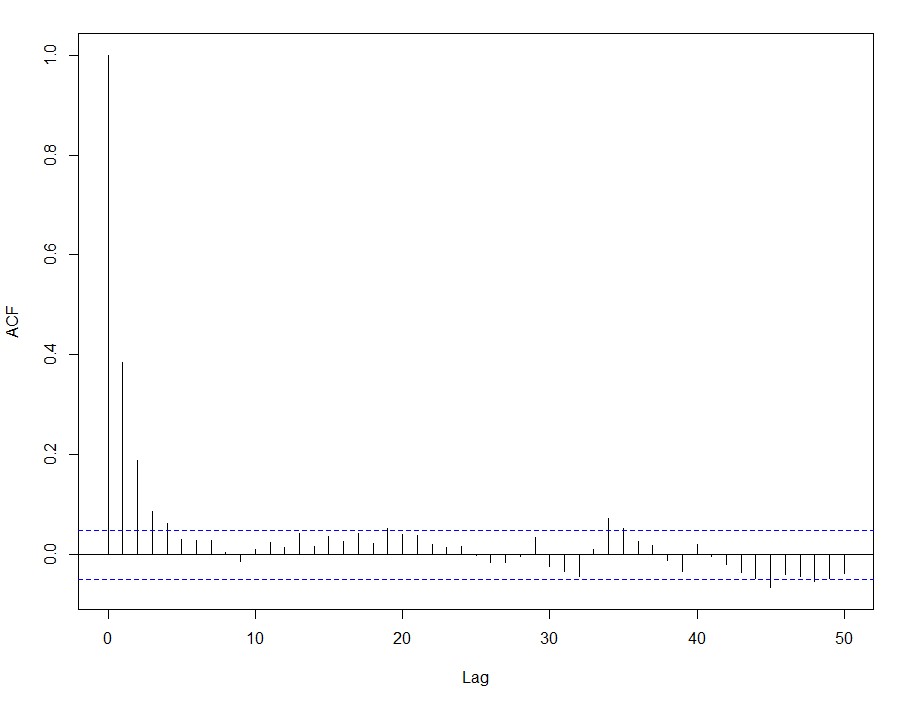


***Zero cells in the cross-tabulation between the exposure, mediator and outcome***

Perfect prediction (or (quasi)-complete separation) is a common problem in regression models with a categorical outcome. This can occur when all subjects in one category of the exposure, are in the same category of a binary outcome, in other words, there is a zero cell in the cross-tabulation between the exposure and the outcome. This is more likely to occur in small datasets, datasets with rare exposure or outcome, or when there is a large underlying effect. When there is an interaction between two exposures on an outcome, a zero cell in the cross-tabulation between both exposures and the outcome can lead to problems with perfect prediction. When using uPCD, the entropy will also be a factor – with low entropy being associated with larger parameter variances meaning that it is more likely that a zero cell will arise during the cycle of the estimation procedure.

For the poor entropy model, 12% of simulated datasets had one zero cell in the cross-tabulation between the exposure, mediator and outcome. To address this we used firth logistic regression to estimate the regression model for *Y* (in both the simulated and applied example) and the regression model for *M* (in the simulated data only) (Heinze & Schemper, 2002). This prevents non-convergence resulting from perfect prediction in the regression models when there is one zero cell. When there are two or more zero cells, non-convergence of the regression model for *Y* can occur, even when using firth logistic regression. For the poor entropy model, 1% (six out of 500 simulated datasets) had two or more zero cells in the cross-tabulation between the exposure, mediator and outcome. For these datasets, we prevented non-convergence in the regression model for *Y* by removing the *XM* interaction within any iteration where multiple zero cells were detected.

***Fixed within-class threshold in the unconditional class derivation model***

In the unconditional latent class model, the class-specific probabilities of the latent class indicators, $P\left( U_{j}=1 \right|X=x)$, can sometimes be 0 or 1 (e.g., reflecting a 0% or 100% probability of indicator *j* in latent class *x*). In this situation, the within-class threshold, $\tau_{jx}$ , is fixed at ±15. Using equation 10, it can be seen that a within-class threshold of -15, is equal to a within-class probability of 1, whereas a within-class threshold of 15, is equal to a within-class probability of 0. When a parameter is fixed at a certain value, it is assumed to have no uncertainty, and a standard error is no longer provided. This results in missing values in the covariance matrix of the parameters from the latent class model (e.g., each threshold that is fixed will have a missing value for its variance and covariance with other parameters). This covariance matrix is exported from M*plus* and used to perturb the parameters. Therefore, when a within-class threshold is fixed, and the (co)variance for this parameter is missing, it cannot be perturbed. This problem was addressed by changing the variance of a fixed within-class threshold from missing to 0.000000001 and changing the covariance with other parameters to 0. This means that the fixed thresholds will still get perturbed but only a very small amount so probabilities will still stay at approximately 100% or 0%. Fixed within-class thresholds in the unconditional latent class model occurred in 5% of simulated datasets with good entropy, 10% of simulated datasets with medium entropy, and 38% of simulated datasets with poor entropy.

***Assessing convergence***

To assess convergence, we examined trace plots of the parameters from the regression model for *Y*, the regression model for *M*, and the cell sizes in the cross-tabulation between the exposure, mediator and outcome. These traceplots plot the same parameters as the ACF plots described earlier, across the 1,700 iterations, although non-convergence is not related to the decisions regarding the number of cycles or imputed datasets detailed above. Non-convergence was seen in some simulated datasets when there was a large standard error for a within-class threshold in the unconditional latent class model. This resulted in instability in the threshold parameters across iterations when performing uPCD due to perturbing the thresholds based on their variance-covariance matrix. To address this, we restricted all within-class thresholds to fall between ±15 after being perturbed (which corresponds to a probability between 0% and 100%). In extreme cases with a very large standard error for a within-class threshold, this still resulted in non-convergence of the parameters. Supplement 3 Figure 3 shows a trace plot of the cell size for *W*=1, *M*=1 and *Y=*1 across iterations using a simulated dataset showing convergence (3A) and another using a simulated dataset that does not show convergence (3B). Simulated datasets were excluded from the dataset of the estimates when the largest within-class threshold standard error from the unconditional latent class model was greater than twice the average of the largest within-class threshold standard errors across all the simulated datasets. For these simulated datasets, we concluded that there was too much uncertainty in model parameters in the class derivation model to use the latent classes in subsequent analyses. This occurred in 4% of simulated datasets with good entropy, 8% of simulated datasets with medium entropy, and 9% of simulated datasets with poor entropy.


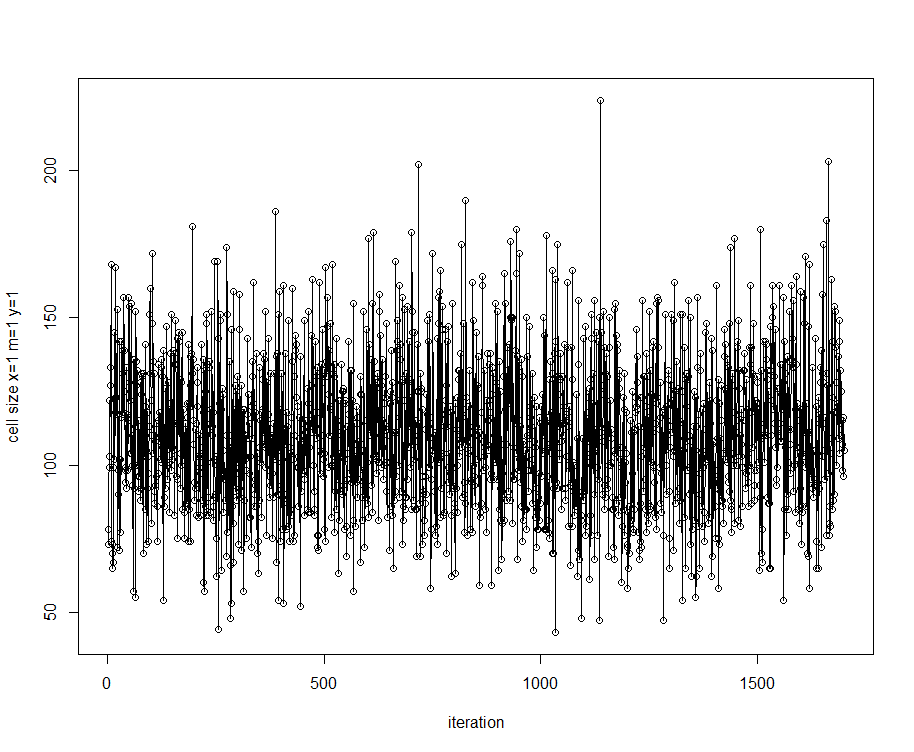

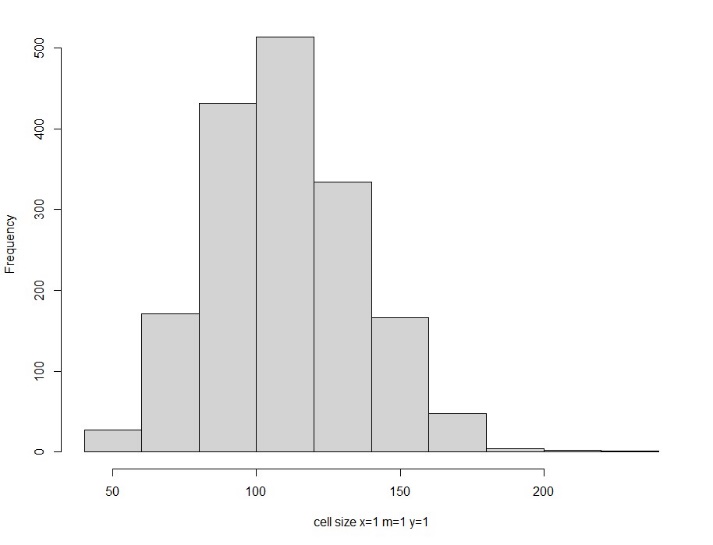
**Supplement 3 Figure 3a** – Trace plot (and corresponding histogram) showing convergence of the cell size for *W*=1, *M*=1 and *Y*=1 across 1,700 iterations using the first simulated dataset with poor entropy


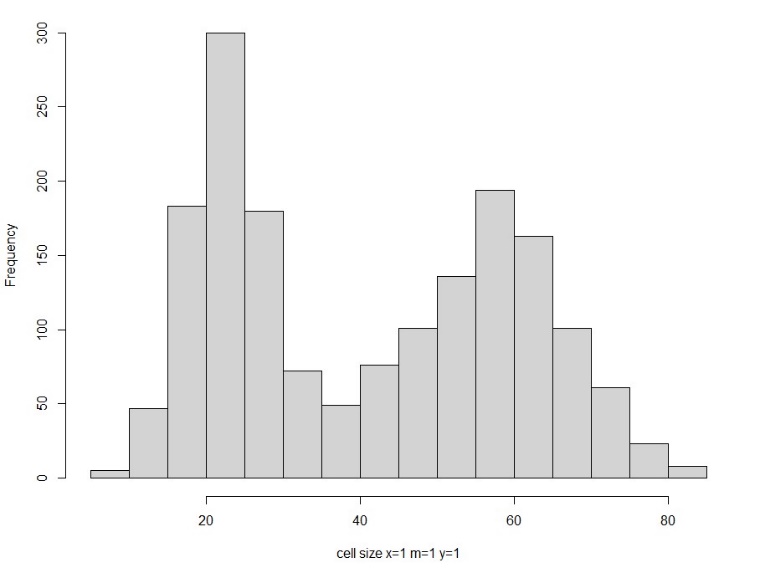

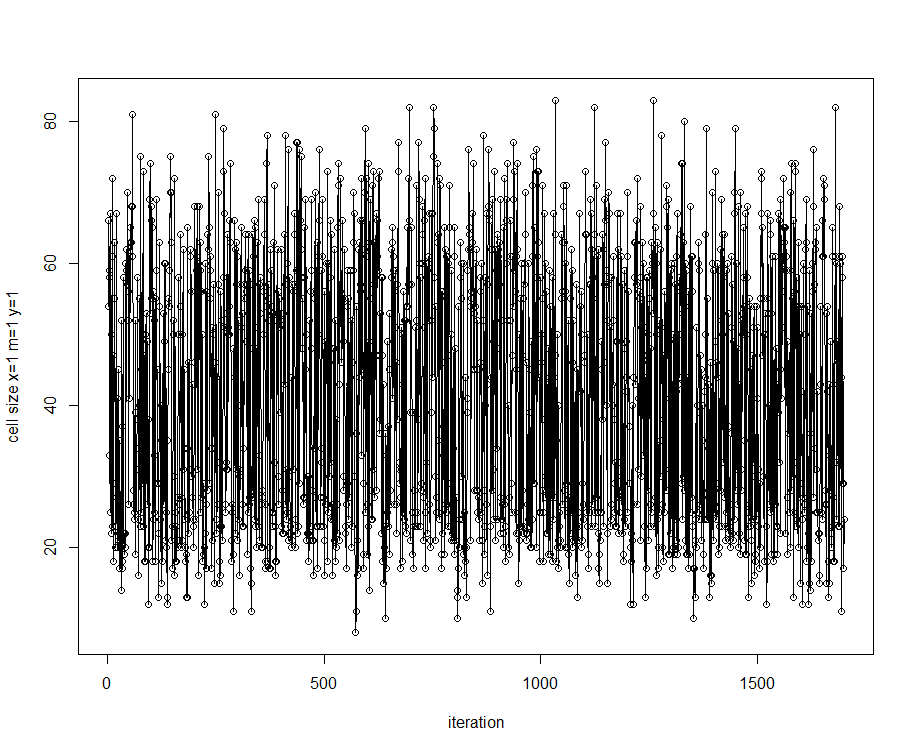
**Supplement 3 Figure 3b** – Trace plot (and corresponding histogram) showing non-convergence of the cell size for *W*=1, *M*=1 and *Y*=1 across 1,700 iterations using a simulated dataset with poor entropy that was excluded from the dataset of the estimates

***Including baseline confounders using a propensity score in the applied example***

In the applied example, sex and a sociodemographic risk score were included as baseline confounders. When confounders are included, the *k* latent class probabilities used in step 2 above need to be replaced by *k* propensity scores (representing the probability of each latent class, given the baseline confounders). These are created by regressing each generated latent class variable, *W*, on the confounders. First, a series of binary logistic regressions were performed (by excluding all but two classes and regressing the remaining classes on the confounders) including the function *detect_separation* to test for the occurrence of complete or quasi-complete separation. This function will detect if any of the parameters have infinite maximum likelihood estimates which is quite common with multinomial logistic regression (required to regress the latent classes on the confounders). If there are no infinite maximum likelihood estimates, a standard multinomial logistic regression is used to regress the latent classes on the confounders e.g., using *multinom*. If there is an infinite parameter in any of the logistic regression models, the latent classes are regressed on the confounders using the *brmultinom* function. The *brmultinom* function (with type = "AS_mean") can be used as an alternative to maximum likelihood estimation in situations where complete or quasi-complete separation is present (<https://www.rdocumentation.org/packages/brglm2/versions/0.8.2/topics/brmultinom>).

The parameters from the multinomial logistic regression model are then perturbed using their (co)variance matrix (as in step 2 above). Finally, one propensity score is calculated for each latent class (representing the probability of each latent class, given the baseline confounders) using the perturbed parameters from the multinomial logistic regression. Each individual’s propensity scores are then used to calculate their probability of latent class membership (in step 2) rather than using the latent class probabilities.

# **Supplement 4** **-** Comparison of estimates and standard errors for simulated datasets that were and weren’t excluded based on convergence criteria

Each figure below plots the estimate (e.g., log risk ratio for the total effect of early-onset persistent versus Low conduct problems) on the x-axis and the corresponding standard error on the y-axis for each simulated dataset across all three data-generating mechanisms (e.g., good, medium, and poor entropy levels). In all plots, simulated datasets that were excluded from the dataset of the estimates (based on convergence criteria) are shown with a light blue dot, whereas all other datasets are shown with a dark blue dot.

As can be seen in the plots below, many of the outliers correspond to datasets that are excluded based on the convergence criteria, particularly for uPCD. However, a few outliers remain when there is poor entropy (across almost all estimands and class comparisons), particularly for the one-step method. This is likely a consequence of using the one-step method to estimate a complex analysis model, which can often result in non-convergence (Vermunt, 2010).

**Supplement 4 Figure 1** - Scatter plot of log risk-ratios versus standard errors for the total effect (TE) of Early-Onset Persistent (EOP) versus Low conduct problems


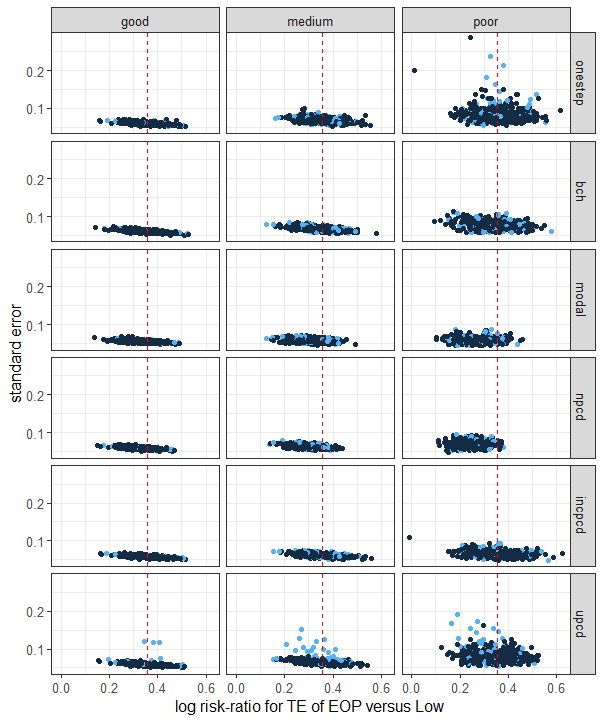


Methods = one-step, bias-adjusted three-step (bch), modal class assignment (modal), non-inclusive PCD (npcd), inclusive PCD (incpcd), and updated PCD (upcd); data-generating mechanisms = good (0.9), medium (0.8), and poor (0.7) entropy levels; each light blue dot represents an excluded simulated dataset, and each dark blue dot represents a simulated datsset used in the main analyses; red dashed line represents log risk-ratio for true value (0.357)

**Supplement 4 Figure 2 -** Scatter plot of log risk-ratios versus standard errors for the total natural indirect effect (TNIE) of Early-Onset Persistent (EOP) versus Low conduct problems


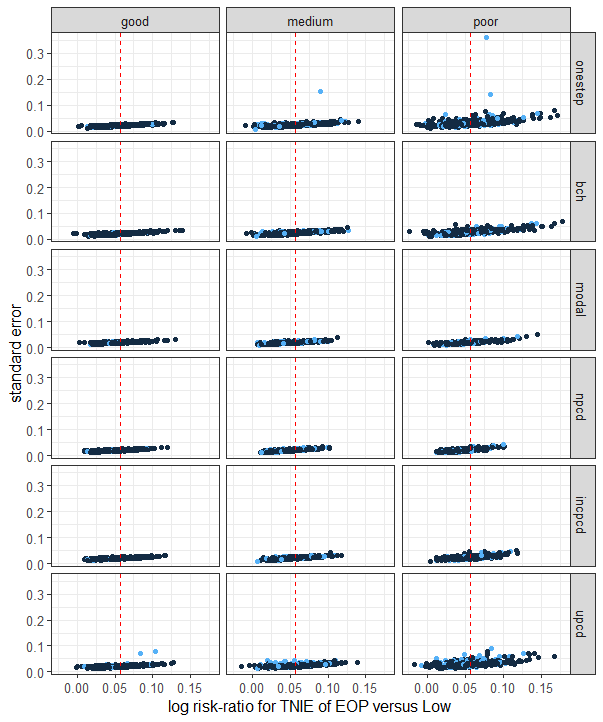


Methods = one-step, bias-adjusted three-step (bch), modal class assignment (modal), non-inclusive PCD (npcd), inclusive PCD (incpcd), and updated PCD (upcd); data-generating mechanisms = good (0.9), medium (0.8), and poor (0.7) entropy levels; each light blue dot represents an excluded simulated dataset, and each dark blue dot represents a simulated datsset used in the main analyses; red dashed line represents log risk-ratio for true value (0.057)

**Supplement 4 Figure 3 -** Scatter plot of log risk-ratios versus standard errors for the pure natural direct effect (PNDE) of Early-Onset Persistent (EOP) versus Low conduct problems


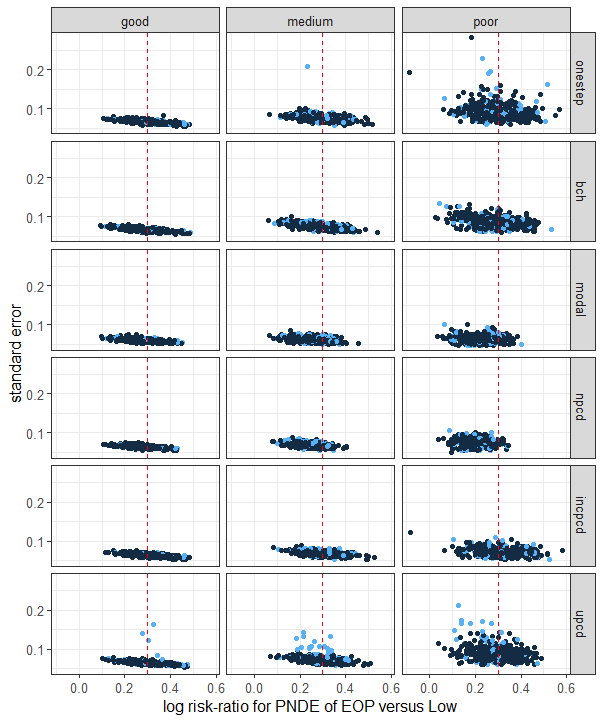


Methods = one-step, bias-adjusted three-step (bch), modal class assignment (modal), non-inclusive PCD (npcd), inclusive PCD (incpcd), and updated PCD (upcd); data-generating mechanisms = good (0.9), medium (0.8), and poor (0.7) entropy levels; each light blue dot represents an excluded simulated dataset, and each dark blue dot represents a simulated datsset used in the main analyses; red dashed line represents log risk-ratio for true value (0.300)

**Supplement 4 Figure 4 -** Scatter plot of log risk-ratios versus standard errors for the total effect (TE) of Adolescent Onset (AO) versus Low conduct problems


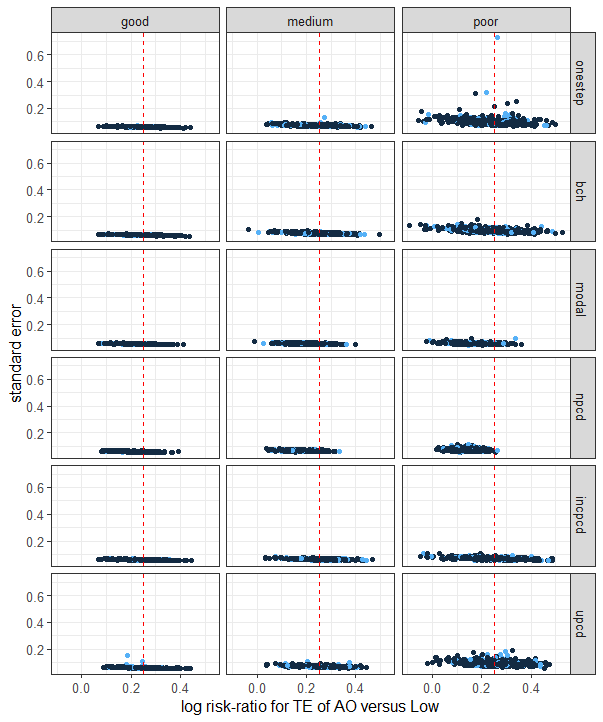


Methods = one-step, bias-adjusted three-step (bch), modal class assignment (modal), non-inclusive PCD (npcd), inclusive PCD (incpcd), and updated PCD (upcd); data-generating mechanisms = good (0.9), medium (0.8), and poor (0.7) entropy levels; each light blue dot represents an excluded simulated dataset, and each dark blue dot represents a simulated datsset used in the main analyses; red dashed line represents log risk-ratio for true value (0.251)

**Supplement 4 Figure 5 -** Scatter plot of log risk-ratios versus standard errors for the total natural indirect effect (TNIE) of Adolescent Onset (AO) versus Low conduct problems


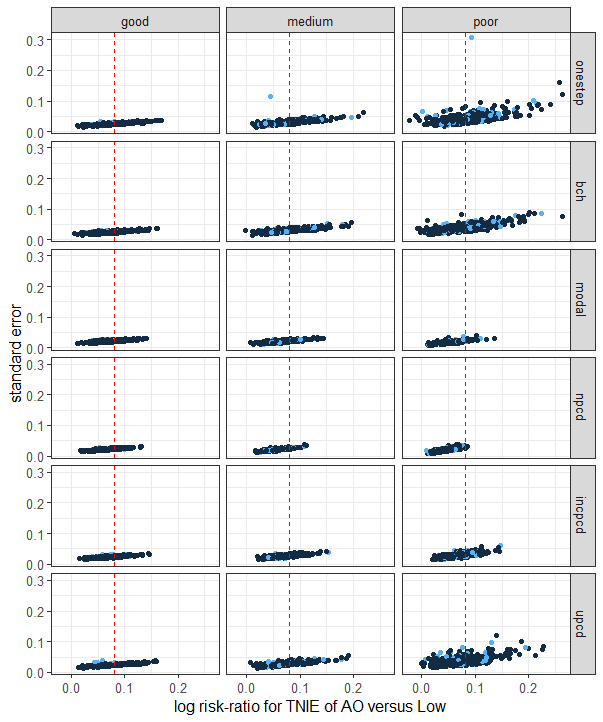


Methods = one-step, bias-adjusted three-step (bch), modal class assignment (modal), non-inclusive PCD (npcd), inclusive PCD (incpcd), and updated PCD (upcd); data-generating mechanisms = good (0.9), medium (0.8), and poor (0.7) entropy levels; each light blue dot represents an excluded simulated dataset, and each dark blue dot represents a simulated datsset used in the main analyses; red dashed line represents log risk-ratio for true value (0.081)

**Supplement 4 Figure 6 -** Scatter plot of log risk-ratios versus standard errors for the pure natural direct effect (PNDE) of Adolescent Onset (AO) versus Low conduct problems


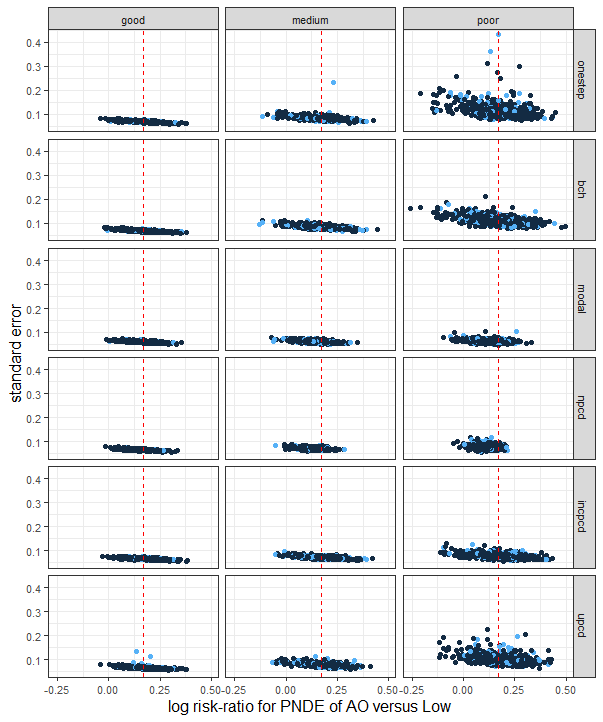


Methods = one-step, bias-adjusted three-step (bch), modal class assignment (modal), non-inclusive PCD (npcd), inclusive PCD (incpcd), and updated PCD (upcd); data-generating mechanisms = good (0.9), medium (0.8), and poor (0.7) entropy levels; each light blue dot represents an excluded simulated dataset, and each dark blue dot represents a simulated datsset used in the main analyses; red dashed line represents log risk-ratio for true value (0.170)

**Supplement 4 Figure 7 -** Scatter plot of log risk-ratios versus standard errors for the total effect (TE) of Childhood Limited (CL) versus Low conduct problems


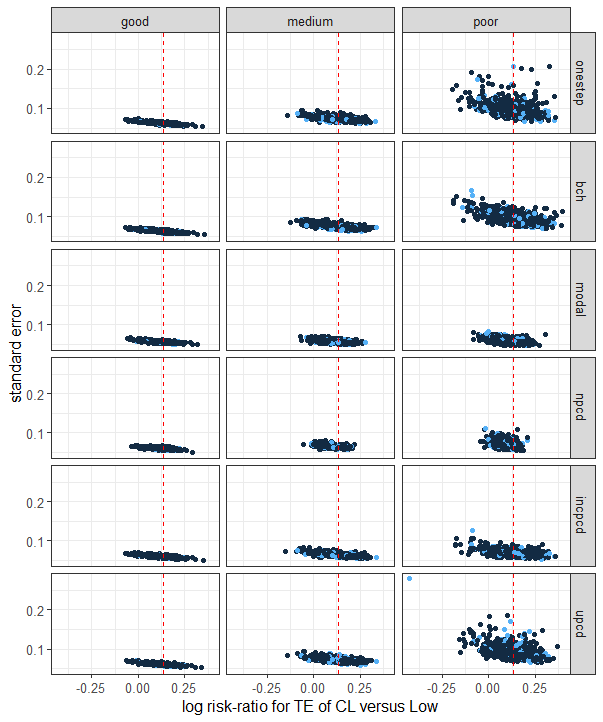


Methods = one-step, bias-adjusted three-step (bch), modal class assignment (modal), non-inclusive PCD (npcd), inclusive PCD (incpcd), and updated PCD (upcd); data-generating mechanisms = good (0.9), medium (0.8), and poor (0.7) entropy levels; each light blue dot represents an excluded simulated dataset, and each dark blue dot represents a simulated datsset used in the main analyses; red dashed line represents log risk-ratio for true value (0.134)

**Supplement 4 Figure 8 -** Scatter plot of log risk-ratios versus standard errors for the total natural indirect effect (TNIE) of Childhood Limited (CL) versus Low conduct problems


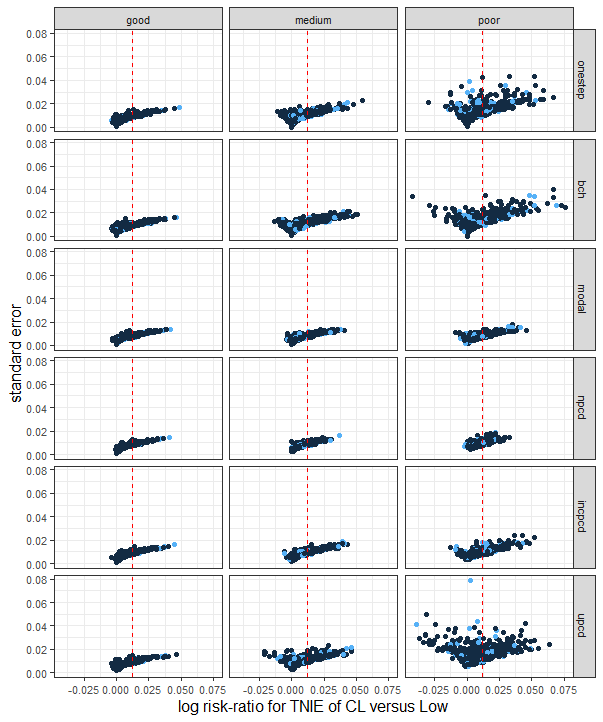


Methods = one-step, bias-adjusted three-step (bch), modal class assignment (modal), non-inclusive PCD (npcd), inclusive PCD (incpcd), and updated PCD (upcd); data-generating mechanisms = good (0.9), medium (0.8), and poor (0.7) entropy levels; each light blue dot represents an excluded simulated dataset, and each dark blue dot represents a simulated datsset used in the main analyses; red dashed line represents log risk-ratio for true value (0.012)

**Supplement 4 Figure 9 -** Scatter plot of log risk-ratios versus standard errors for the pure natural direct effect (PNDE) of Childhood Limited (CL) versus Low conduct problems


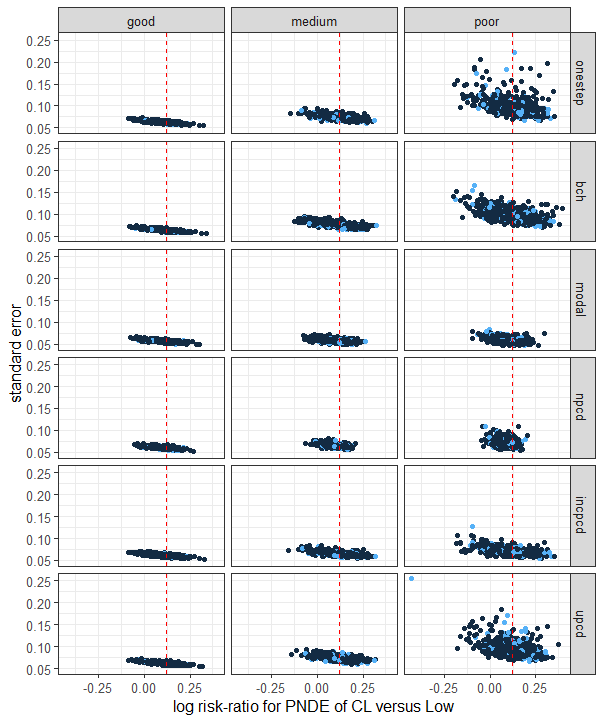


Methods = one-step, bias-adjusted three-step (bch), modal class assignment (modal), non-inclusive PCD (npcd), inclusive PCD (incpcd), and updated PCD (upcd); data-generating mechanisms = good (0.9), medium (0.8), and poor (0.7) entropy levels; each light blue dot represents an excluded simulated dataset, and each dark blue dot represents a simulated datsset used in the main analyses; red dashed line represents log risk-ratio for true value (0.122)

# **Supplement 5** - Bias in mediation effects by method, data generating mechanism, and latent class comparison before exclusions; *N* = 500 simulated datasets with good entropy (0.9), medium entropy (0.8), and poor entropy (0.7)

**Supplement 5 Figure 1 -** Bias (and 95% confidence intervals based on Monte Carlo standard errors) in the total effect (TE) by method, data generating mechanism, and latent class comparison (EOP, AO, and CL versus Low)


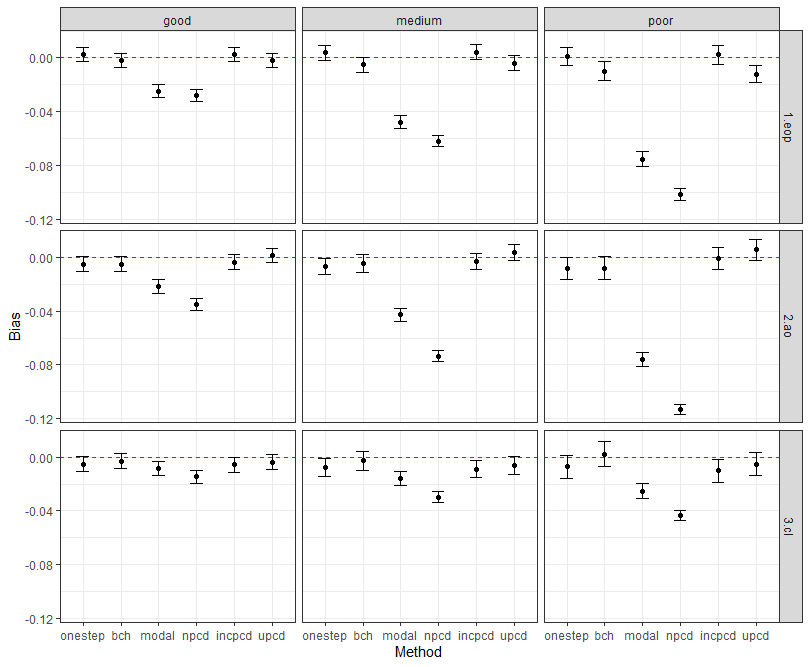


**Supplement 5 Figure 2 -** Bias (and 95% confidence intervals based on Monte Carlo standard errors) in the total natural indirect effect (TNIE) by method, data generating mechanism, and latent class comparison (EOP, AO, and CL versus Low)


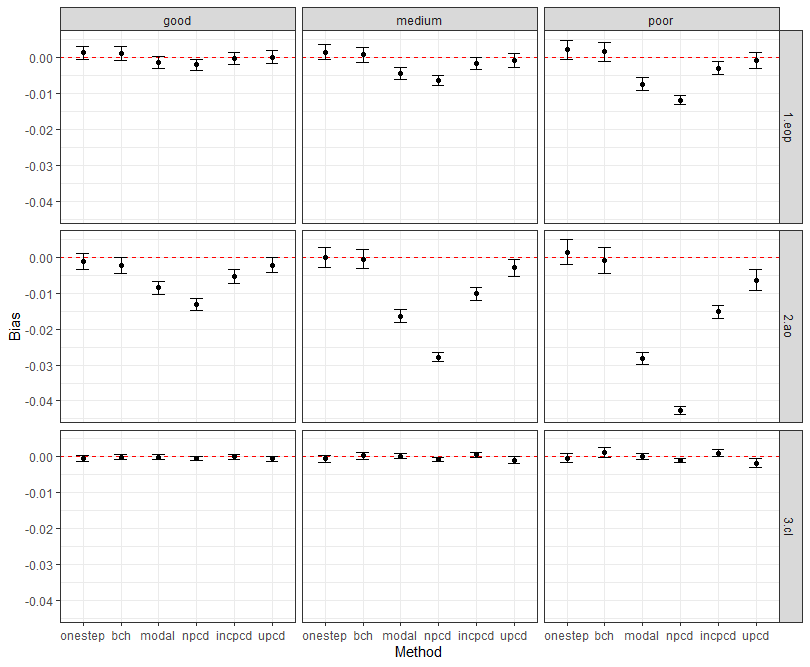


**Supplement 5 Figure 3 -** Bias (and 95% confidence intervals based on Monte Carlo standard errors) in the pure natural direct effect (PNDE) by method, data generating mechanism, and latent class comparison (EOP, AO, and CL versus Low)


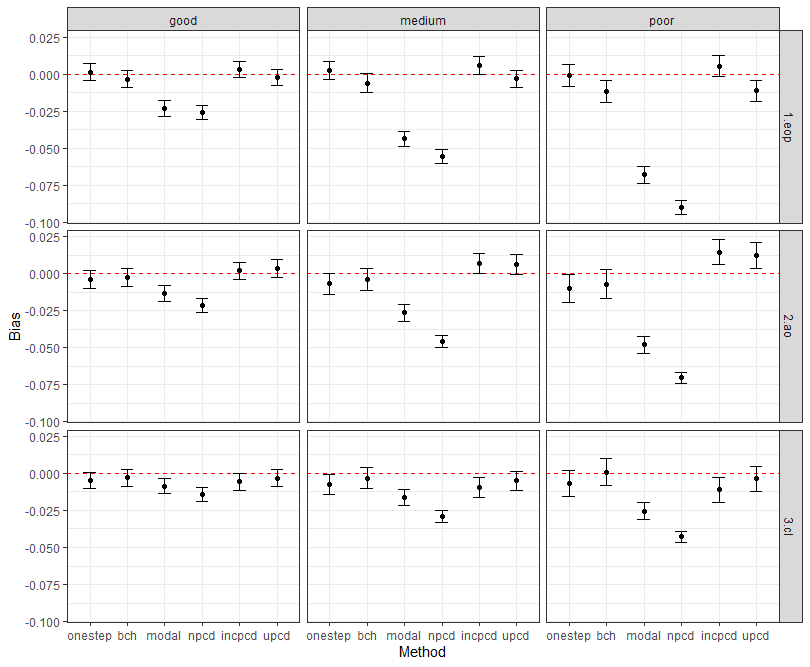


# **Supplementary Table 2** - Performance measures (Monte Carlo standard errors) for each method and data generating mechanism for the total effect (TE) of Adolescent Onset versus Low conduct problems (true value = 0.251); *N* = 457 simulated datasets with good entropy (0.9), *N* = 434 simulated datasets with medium entropy (0.8), *N* = 424 simulated datasets with poor entropy (0.7)

| Performance measure (for θ) | Data-generating mechanism | One-step | Bias-adjusted three-step (BCH) | Modal class assignment | Non-inclusive pseudo class draws | Inclusive pseudo class draws | Updated pseudo class draws |
| --- | --- | --- | --- | --- | --- | --- | --- |
| Bias;  % bias | Good entropy | -0.004 (0.003); 1.5% | -0.004 (0.003); 1.5% | -0.021 (0.003); 8.2% | -0.03 (0.002); 13.5% | -0.002 (0.003); 0.8% | 0.003 (0.003); 1.1% |
|  | Medium entropy | -0.007 (0.003); 2.8% | -0.005 (0.004); 1.9% | -0.043 (0.003); 17.1% | -0.074 (0.002); 29.3% | -0.003 (0.003); 1.4% | 0.003 (0.003); 1.3% |
|  | Poor entropy | -0.009 (0.005); 3.8% | -0.009 (0.005); 3.6% | -0.077 (0.003); 30.7% | -0.114 (0.002); 45.4% | -0.002 (0.005); 0.8% | 0.004 (0.004); 1.7% |
| Coverage | Good entropy | 93.2% (0.012) | 93.7% (0.011) | 93.7% (0.011) | 94.7% (0.010) | 92.6% (0.012) | 93.7% (0.011) |
|  | Medium entropy | 96.1% (0.009) | 95.6% (0.010) | 90.6% (0.014) | 91.7% (0.013) | 92.4% (0.013) | 96.1% (0.009) |
|  | Poor entropy | 95.3% (0.010) | 95.8% (0.010) | 76.2% (0.021) | 75.7% (0.021) | 86.3% (0.017) | 95.3% (0.010) |
| Bias-eliminated coverage | Good entropy | 94.1% (0.011) | 93.4% (0.012) | 93.7% (0.011) | 96.9% (0.008) | 91.9% (0.013) | 93.7% (0.011) |
|  | Medium entropy | 96.3% (0.009) | 95.4% (0.010) | 95.4% (0.010) | 99.3% (0.004) | 93.1% (0.012) | 96.3% (0.009) |
|  | Poor entropy | 94.8% (0.011) | 95.3% (0.010) | 95.0% (0.011) | 99.8% (0.002) | 86.3% (0.017) | 95.5% (0.010) |
| Empirical standard error | Good entropy | 0.061 (0.002) | 0.063 (0.002) | 0.057 (0.002) | 0.051 (0.002) | 0.062 (0.002) | 0.061 (0.002) |
|  | Medium entropy | 0.069 (0.002) | 0.073 (0.002) | 0.056 (0.002) | 0.044 (0.001) | 0.069 (0.002) | 0.067 (0.002) |
|  | Poor entropy | 0.095 (0.003) | 0.099 (0.003) | 0.062 (0.002) | 0.043 (0.001) | 0.095 (0.003) | 0.090 (0.003) |
| Average model standard error | Good entropy | 0.061 (0.0001) | 0.062 (0.0001) | 0.056 (0.0001) | 0.061 (0.0001) | 0.059 (0.0001) | 0.060 (0.0001) |
|  | Medium entropy | 0.074 (0.0003) | 0.076 (0.0003) | 0.058 (0.0002) | 0.067 (0.0002) | 0.065 (0.0002) | 0.072 (0.0003) |
|  | Poor entropy | 0.103 (0.002) | 0.099 (0.001) | 0.061 (0.0003) | 0.074 (0.0004) | 0.069 (0.0004) | 0.095 (0.001) |

# **Supplementary Table 3** - Performance measures (Monte Carlo standard errors) for each method and data generating mechanism for the total effect (TE) of Childhood Limited versus Low conduct problems (true value = 0.134); *N* = 457 simulated datasets with good entropy (0.9), *N* = 434 simulated datasets with medium entropy (0.8), *N* = 424 simulated datasets with poor entropy (0.7)

| Performance measure (for θ) | Data-generating mechanism | One-step | Bias-adjusted three-step (BCH) | Modal class assignment | Non-inclusive pseudo class draws | Inclusive pseudo class draws | Updated pseudo class draws |
| --- | --- | --- | --- | --- | --- | --- | --- |
| Bias; % bias | Good entropy | -0.006 (0.003); 4.8% | -0.004 (0.003); 3.3% | -0.010 (0.003); 7.5% | -0.016 (0.002); 11.6% | -0.007 (0.003); 5.1% | -0.005 (0.003); 3.6% |
|  | Medium entropy | -0.008 (0.004); 6.1% | -0.003 (0.004); 2.4% | -0.016 (0.003); 12.1% | -0.030 (0.002); 22.3% | -0.009 (0.004); 6.8% | -0.007 (0.004); 5.2% |
|  | Poor entropy | -0.009 (0.005); 6.5% | 0.002 (0.005); 1.6% | -0.025 (0.003); 18.8% | -0.044 (0.002); 32.9% | -0.012 (0.005); 8.8% | -0.007 (0.005); 5.2% |
| Coverage | Good entropy | 94.1% (0.011) | 92.8% (0.012) | 92.8% (0.012) | 96.5% (0.009) | 93.2% (0.012) | 94.3% (0.011) |
|  | Medium entropy | 94.9% (0.011) | 94.5% (0.011) | 92.2% (0.013) | 98.2% (0.006) | 90.6% (0.014) | 94.9% (0.011) |
|  | Poor entropy | 96.7% (0.009) | 92.7% (0.013) | 92.5% (0.013) | 99.3% (0.004) | 84.7% (0.017) | 96.2% (0.009) |
| Bias-eliminated coverage | Good entropy | 93.9% (0.011) | 93.4% (0.012) | 93.0% (0.012) | 97.4% (0.007) | 93.2% (0.012) | 94.5% (0.011) |
|  | Medium entropy | 95.9% (0.010) | 94.5% (0.011) | 94.5% (0.011) | 99.8% (0.002) | 90.4% (0.014) | 95.4% (0.010) |
|  | Poor entropy | 96.0% (0.010) | 92.7% (0.013) | 94.1% (0.011) | 100% (-) | 85.1% (0.017) | 95.8% (0.010) |
| Empirical standard error | Good entropy | 0.064 (0.002) | 0.065 (0.002) | 0.059 (0.002) | 0.052 (0.002) | 0.064 (0.002) | 0.064 (0.002) |
|  | Medium entropy | 0.076 (0.003) | 0.081 (0.003) | 0.061 (0.002) | 0.046 (0.002) | 0.076 (0.003) | 0.074 (0.003) |
|  | Poor entropy | 0.098 (0.003) | 0.106 (0.004) | 0.064 (0.002) | 0.041 (0.001) | 0.098 (0.003) | 0.095 (0.003) |
| Average model standard error | Good entropy | 0.062 (0.0001) | 0.063 (0.0001) | 0.056 (0.0001) | 0.060 (0.0001) | 0.060 (0.0001) | 0.061 (0.0001) |
|  | Medium entropy | 0.074 (0.0003) | 0.076 (0.0003) | 0.058 (0.0002) | 0.065 (0.0002) | 0.066 (0.0002) | 0.073 (0.0003) |
|  | Poor entropy | 0.102 (0.001) | 0.097 (0.001) | 0.059 (0.0003) | 0.071 (0.0004) | 0.071 (0.0004) | 0.096 (0.001) |


# **Supplement 6 -** Performance measures (Monte Carlo standard errors) for each method and data generating mechanism using a reduced sample size for the simulation of *n_ob_*_s_ = 2,000

We simulated data using the same data-generating mechanisms but reduced the sample size to *n_ob_*_s_ = 2,000 to evaluate how each method performed on a smaller sample size.

***Convergence***

We excluded some simulated datasets due to very large standard error (SE) for some within-class thresholds (representing within-class probability for a latent class indicator) in either the unconditional latent class model or the one-step model. When this was the case, the simulated dataset was excluded across all six methods. Eight percent of simulated datasets with good entropy (n = 16 for unconditional latent class model, n = 21 for one-step model, n = 3 for both), 9% with medium entropy (n = 25 for unconditional latent class model, n = 16 for one-step model, n = 3 for both), and 14% with poor entropy (n = 35 for unconditional latent class model, n = 29 for one-step model, n = 4 for both) were excluded. A further 17 simulated datasets with poor entropy (3%) were excluded because there was an imputed latent class with a prevalence of zero in at least one of the iterations when running uPCD. This was a consequence of multiple zero cells in the cross-tabulation between the exposure, mediator and outcome and resulted in non-convergence of the regression model for *Y* and for *M.* In total, 85 simulated datasets (17%) with poor entropy were excluded from the analysis of the estimates dataset.

***Performance measures***

Supplement 6 Figure 1 shows the bias and 95% confidence intervals (based on MCSE) by method, data generating mechanism, and latent class comparison, after excluding datasets based on convergence criteria above. Supplement 6 Figure 1a shows the bias in the TE, Supplement 6 Figure 1b plots the bias in the TNIE, and Supplement 6 Figure 1c plots the bias in the PNDE. As shown in the Figures, uPCD shows minimal bias which is comparable to one-step and bias-adjusted three-step models across all entropy levels, estimands, and class comparisons. Additionally, incPCD shows minimal bias with the exception of the TNIE of AO versus Low conduct problems where there is bias some towards the null (seen to a lesser extent with uPCD in the poor entropy model). The greatest bias is for nPCD, followed by modal class assignment, with bias towards the null, particularly when the entropy is poor. The only exception to this is when the effect size is close to zero (TNIE of CL versus Low conduct problems; true effect = 0.012), and all methods have minimal bias across all entropy levels.

**Supplement 6 Figure 1a** - Bias (and 95% confidence intervals based on Monte Carlo standard errors) in the total effect (TE) by method, data generating mechanism, and latent class comparison; *N* = 460 simulated datasets with good entropy (0.9), *N* = 456 simulated datasets with medium entropy (0.8), *N* = 415 simulated datasets with poor entropy (0.7); Methods = one-step, bias-adjusted three-step (bch), modal class assignment (modal), non-inclusive PCD (npcd), inclusive PCD (incpcd), and updated PCD (upcd); data-generating mechanisms = good, medium and poor entropy levels; latent class comparisons = Early-Onset Persistent (1.eop) versus Low: true value = 0.357, Adolescent Onset (2.ao) versus Low: true value = 0.251; Childhood Limited (3.cl) versus Low: true value = 0.134)


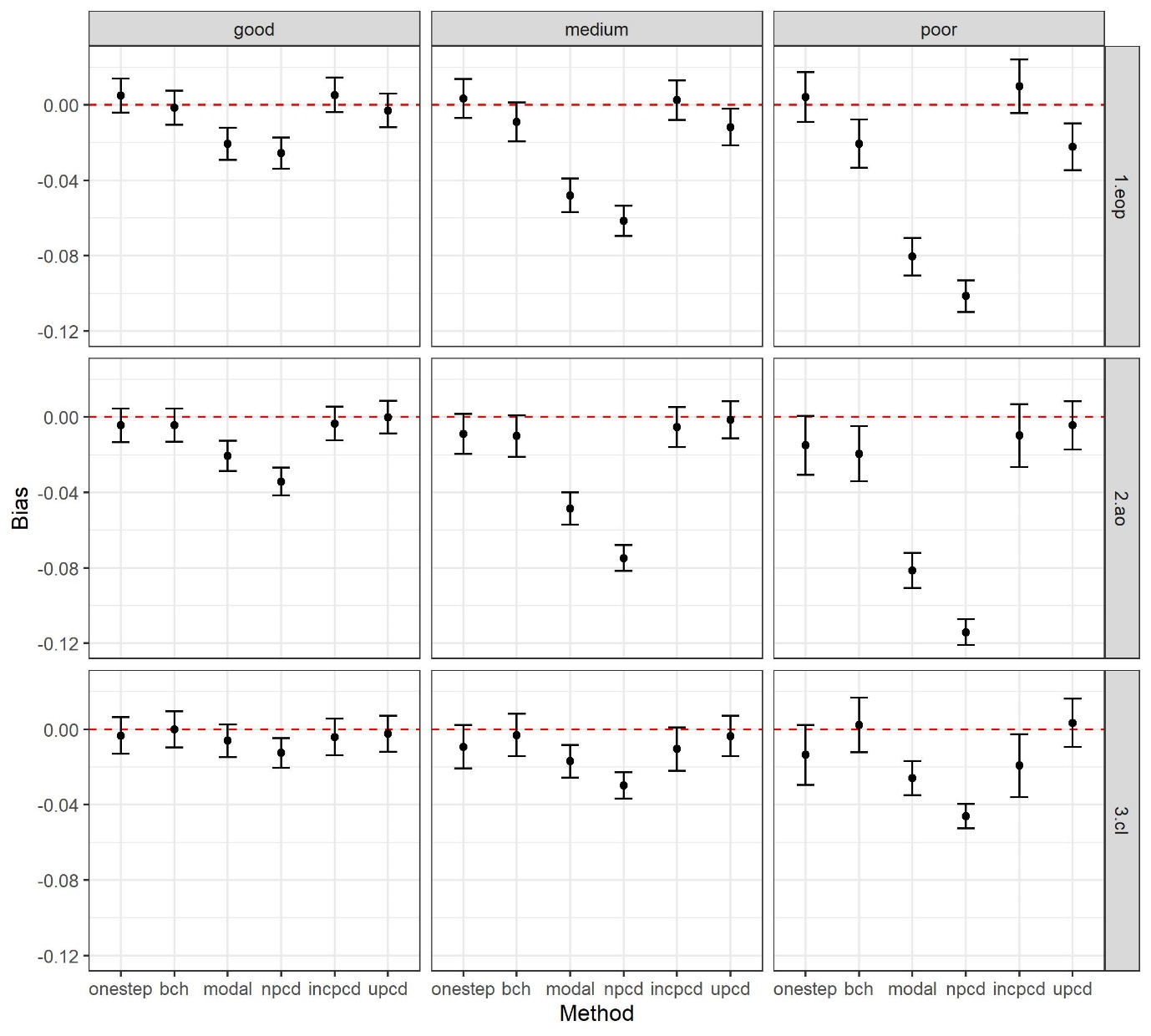


**Supplement 6 Figure 1b** - Bias (and 95% confidence intervals based on Monte Carlo standard errors) in the total natural indirect effect (TNIE) by method, data generating mechanism, and latent class comparison; *N* = 460 simulated datasets with good entropy (0.9), *N* = 456 simulated datasets with medium entropy (0.8), *N* = 415 simulated datasets with poor entropy (0.7); Methods = one-step, bias-adjusted three-step (bch), modal class assignment (modal), non-inclusive PCD (npcd), inclusive PCD (incpcd), and updated PCD (upcd); data-generating mechanisms = good, medium and poor entropy levels; latent class comparisons = Early-Onset Persistent (1.eop) versus Low: true value = 0.057, Adolescent Onset (2.ao) versus Low: true value = 0.081; Childhood Limited (3.cl) versus Low: true value = 0.012)


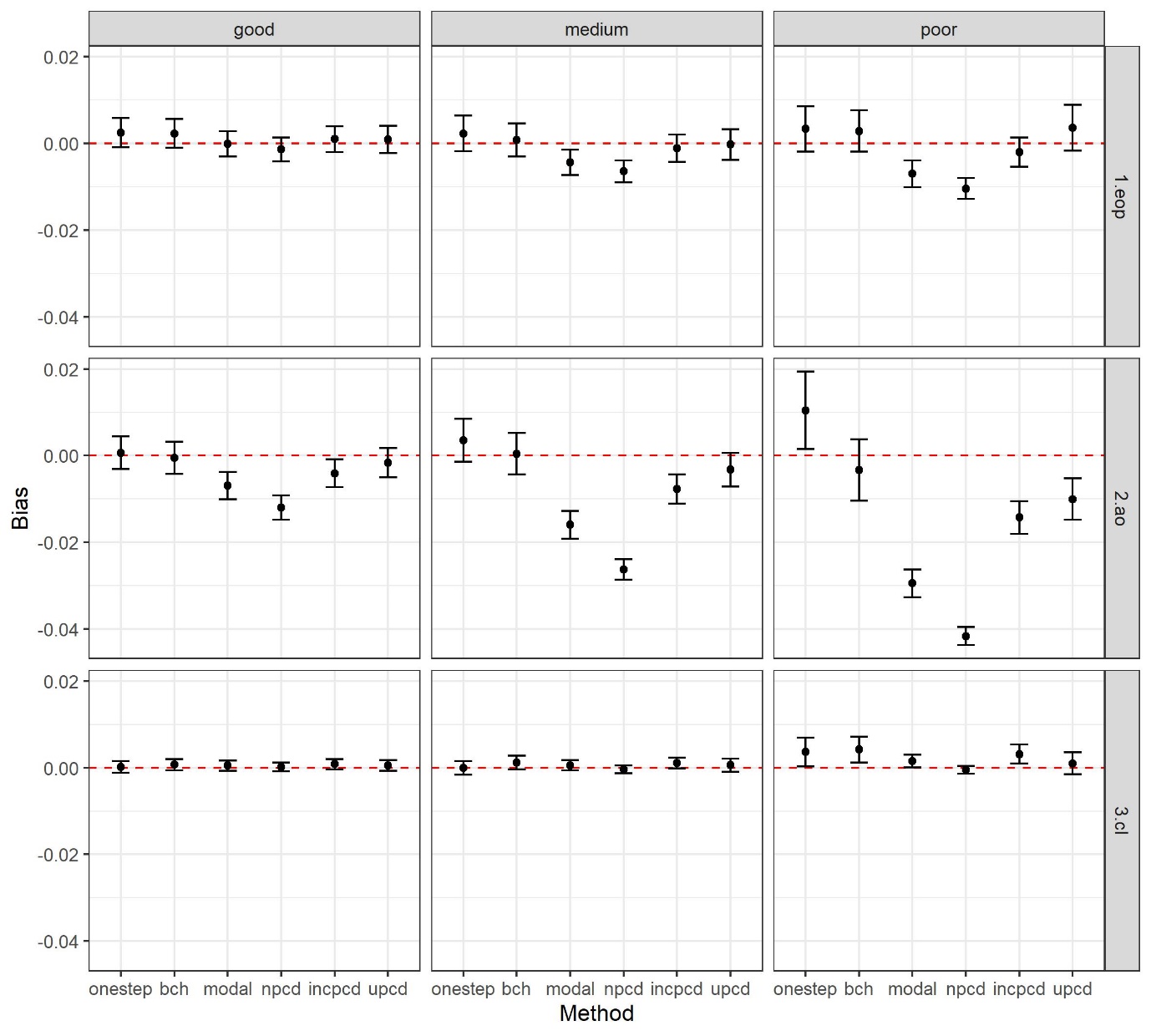


**Supplement 6 Figure 1c** - Bias (and 95% confidence intervals based on Monte Carlo standard errors) in the pure natural direct effect (PNDE) by method, data generating mechanism, and latent class comparison; *N* = 460 simulated datasets with good entropy (0.9), *N* = 456 simulated datasets with medium entropy (0.8), *N* = 415 simulated datasets with poor entropy (0.7); Methods = one-step, bias-adjusted three-step (bch), modal class assignment (modal), non-inclusive PCD (npcd), inclusive PCD (incpcd), and updated PCD (upcd); data-generating mechanisms = good, medium and poor entropy levels; latent class comparisons = Early-Onset Persistent (1.eop) versus Low: true value = 0.300, Adolescent Onset (2.ao) versus Low: true value = 0.170; Childhood Limited (3.cl) versus Low: true value = 0.122)


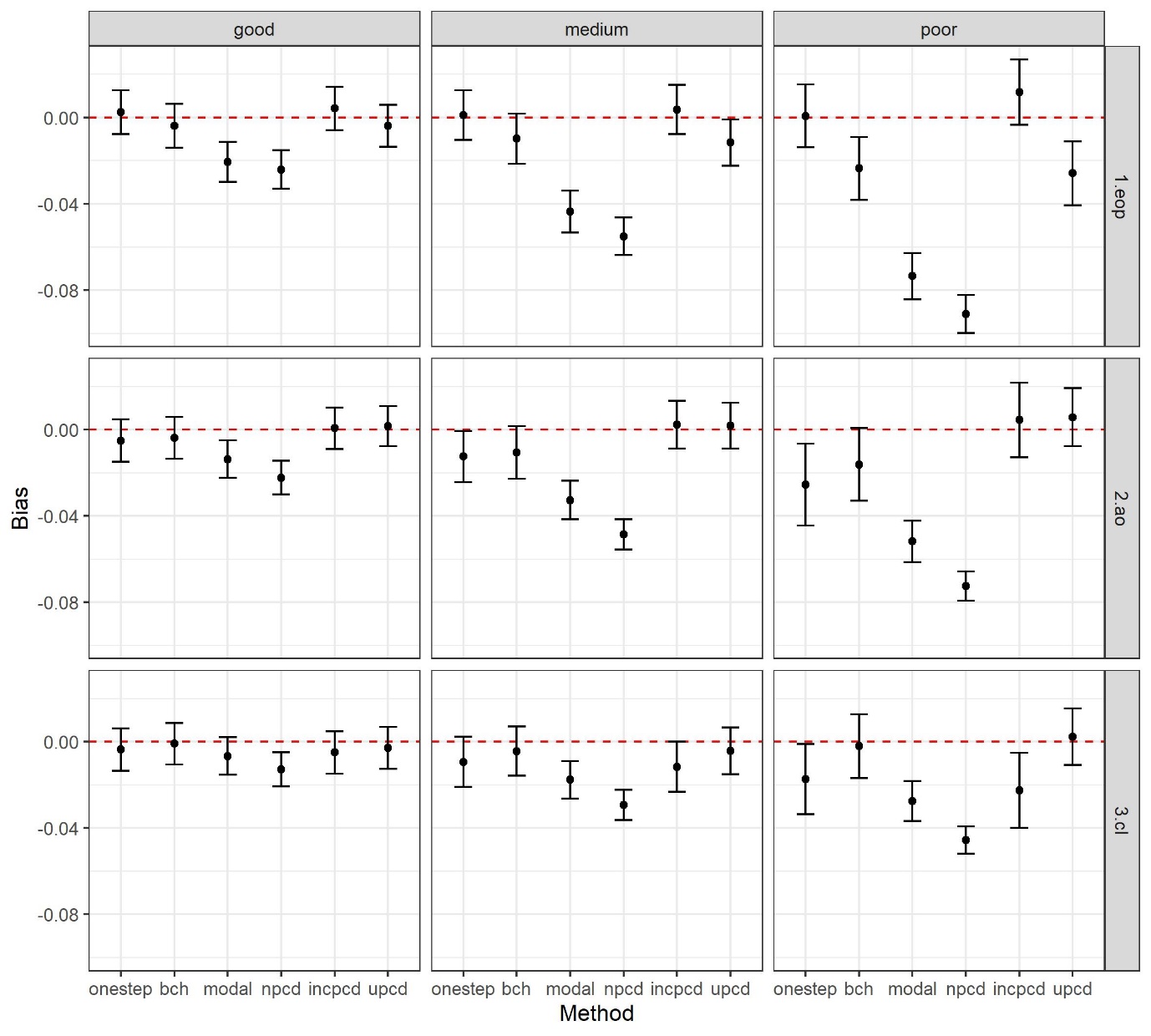


All performance measures (and MCSE) for each method, data generating mechanism, and estimand (after excluding datasets based on convergence criteria above) are shown in Supplement 6 Table 1 (a, b, and c) below for the effects of EOP versus Low conduct problems.

Supplement 6 Table 1a shows performance measures for the TE. As shown in Supplement 6 Table 1a, modal class assignment and nPCD have the largest percentage bias across all entropy levels (23% and 29% respectively, for the poor entropy model). Bias-adjusted three-step (<6%), incPCD (<3%) and uPCD (<7%) have small levels of percentage bias across all entropy levels, which are similar to the levels of percentage bias for the one-step model (<2%). Decreasing entropy increases uncertainty, and this is reflected in the empirical SE which show decreasing precision with decreasing entropy for all methods, except for modal class assignment and nPCD which are overly precise at all entropy levels and also fail to capture the increasing uncertainty. Model-based SE are close to empirical SE with exception of nPCD (where model-based SE are overestimated by 26% for the poor entropy model) and incPCD (where model-based SE are underestimated by 30% for the poor entropy model). Additionally, model-based SE in the poor entropy model are overestimated for one-step (28%) and uPCD (23%). The coverage of nominal 95% confidence intervals is close to 95% for one-step, bias-adjusted three-step, and uPCD across all entropy levels, but there is under-coverage for modal class assignment, nPCD, and incPCD, particularly with poor entropy (modal class assignment = 83%, nPCD = 89%, incPCD = 87%). This is driven by bias for modal class assignment and nPCD, with the bias-eliminated coverage showing over-coverage for nPCD (98% for poor entropy model) which is a result of a model SE greater than the empirical SE. For incPCD, there is still under coverage after accounting for bias in the poor entropy model (87%), which is a result of a model SE smaller than the empirical SE.

Supplement 6 Table 1b shows performance measures for the TNIE. The pattern of results is similar to the TE, with modal class assignment and nPCD having the largest bias particularly for medium and poor entropy models. Bias-adjusted three-step (<6%), incPCD (<4%) and uPCD (<7%) have small levels of percentage bias across all entropy levels, which is similar to the levels of percentage bias for the one-step model (<6%). The empirical SE show that modal class assignment, nPCD, and incPCD are overly precise. Again, model-based SE are close to empirical SE with exception of nPCD (where model-based SE are overestimated by 38% for the poor entropy model) and incPCD (where model-based SE are overestimated by 23% for the poor entropy model). This is in contrast to results for the TE (where model-based SE were overly precise for incPCD). Additionally, model-based SE in the poor entropy model are overestimated for the one-step model (24%). The coverage of nominal 95% confidence intervals is acceptable for all methods, across all entropy levels with the exception of modal class assignment where there is under-coverage, particularly with poor entropy (modal class assignment = 88%) which is partly driven by bias. In the poor entropy model, there is also slight under-coverage for onestep and bias-adjusted three-step (coverage and bias-eliminated coverage = 93% for both methods), and slight over-coverage for nPCD and uPCD (bias-eliminated coverage = 98% for nPCD and 97% for uPCD). The latter is a result of a model SE greater than the empirical SE in the poor entropy model.

Supplement 6 Table 1c shows performance measures for the PNDE. Again, the pattern of results is similar, with modal class assignment and nPCD having the largest bias, and bias-adjusted three-step (<8%), incPCD (<4%) and uPCD (<9%) showing small levels of percentage bias, which are similar to the one-step model (<1%). The empirical SE shows that modal class assignment and nPCD are overly precise. Again, model-based SE are close to empirical SE with exception of nPCD (where model-based SE are overestimated by 29% for the poor entropy model) and incPCD (where model-based SE are underestimated by 25% for the poor entropy model). Additionally, model-based SE in the poor entropy model are overestimated for one-step (22%) and uPCD (17%). After accounting for bias, the coverage of bias-eliminated nominal 95% confidence intervals is close to 95% for one-step and uPCD across all entropy levels. However, there is over-coverage for nPCD and under-coverage for incPCD, particularly with poor entropy (nPCD = 99%, incPCD = 87%). Additionally, there is slight under-coverage for bias-adjusted three step and modal class assignment across all entropy levels.

**Supplement 6 Table 1a -** Performance measures (Monte Carlo standard errors) for each method and data generating mechanism for the total effect (TE) of Early-Onset Persistent versus Low conduct problems (true value = 0.357); *N* = 460 simulated datasets with good entropy (0.9), *N* = 456 simulated datasets with medium entropy (0.8), *N* = 415 simulated datasets with poor entropy (0.7)

| Performance measure (for θ) | Data-generating mechanism | One-step | Bias-adjusted three-step (BCH) | Modal class assignment | Non-inclusive pseudo class draws | Inclusive pseudo class draws | Updated pseudo class draws |
| --- | --- | --- | --- | --- | --- | --- | --- |
| Bias; % bias | Good entropy | 0.005 (0.005); 1.4% | -0.002 (0.005); 0.4% | -0.021 (0.004); 5.8% | -0.026 (0.004); 7.2% | 0.005 (0.005); 1.5% | -0.003 (0.005); 0.8% |
|  | Medium entropy | 0.003 (0.005); 1.0% | -0.009 (0.005); 2.5% | -0.048 (0.005); 13.5% | -0.061 (0.004); 17.2% | 0.003 (0.005); 0.7% | -0.012 (0.005); 3.3% |
|  | Poor entropy | 0.004 (0.007); 1.2% | -0.021 (0.007); 5.8% | -0.080 (0.005); 22.6% | -0.101 (0.004); 28.4% | 0.010 (0.007); 2.7% | -0.022 (0.006); 6.2% |
| Coverage | Good entropy | 94.1% (0.011) | 93.9% (0.011) | 92.6% (0.012) | 94.8% (0.010) | 92.8% (0.012) | 94.8% (0.010) |
|  | Medium entropy | 94.7% (0.010) | 93.4% (0.012) | 90.4% (0.014) | 95.4% (0.010) | 90.8% (0.014) | 95.6% (0.010) |
|  | Poor entropy | 93.5% (0.012) | 94.2% (0.011) | 82.7% (0.019) | 89.4% (0.015) | 86.5% (0.017) | 96.4% (0.009) |
| Bias-eliminated coverage | Good entropy | 94.1% (0.011) | 93.9% (0.011) | 92.6% (0.012) | 95.4% (0.010) | 93.0% (0.012) | 94.8% (0.010) |
|  | Medium entropy | 94.7% (0.010) | 92.3% (0.012) | 93.0% (0.012) | 97.4% (0.007) | 91.4% (0.013) | 95.2% (0.010) |
|  | Poor entropy | 93.7% (0.012) | 94.2% (0.011) | 94.2% (0.011) | 98.3% (0.006) | 87.2% (0.016) | 96.9% (0.009) |
| Empirical standard error | Good entropy | 0.100 (0.003) | 0.100 (0.003) | 0.093 (0.003) | 0.090 (0.003) | 0.100 (0.003) | 0.097 (0.003) |
|  | Medium entropy | 0.112 (0.004) | 0.113 (0.004) | 0.097 (0.003) | 0.088 (0.003) | 0.114 (0.004) | 0.106 (0.004) |
|  | Poor entropy | 0.138 (0.005) | 0.133 (0.005) | 0.104 (0.004) | 0.088 (0.003) | 0.149 (0.005) | 0.130 (0.005) |
| Average model standard error | Good entropy | 0.098 (0.001) | 0.095 (0.0004) | 0.088 (0.0004) | 0.094 (0.0003) | 0.092 (0.0004) | 0.098 (0.001) |
|  | Medium entropy | 0.114 (0.001) | 0.105 (0.001) | 0.090 (0.0005) | 0.101 (0.0005) | 0.098 (0.001) | 0.113 (0.001) |
|  | Poor entropy | 0.176 (0.016) | 0.122 (0.001) | 0.094 (0.001) | 0.111 (0.001) | 0.105 (0.001) | 0.160 (0.020) |

**Supplement 6 Table 1b -** Performance measures (Monte Carlo standard errors) for each method and data generating mechanism for the total natural indirect effect (TNIE) of Early-Onset Persistent versus Low conduct problems (true value = 0.057); *N* = 460 simulated datasets with good entropy (0.9), *N* = 456 simulated datasets with medium entropy (0.8), *N* = 415 simulated datasets with poor entropy (0.7)

| Performance measure (for θ) | Data-generating mechanism | One-step | Bias-adjusted three-step (BCH) | Modal class assignment | Non-inclusive pseudo class draws | Inclusive pseudo class draws | Updated pseudo class draws |
| --- | --- | --- | --- | --- | --- | --- | --- |
| % Bias | Good entropy | 0.002 (0.002); 4.4% | 0.002 (0.002); 4.1% | -0.0001 (0.001); 0.2% | -0.001 (0.001); 2.4% | 0.001 (0.002); 1.7% | 0.001 (0.002); 1.5% |
|  | Medium entropy | 0.002 (0.002); 4.0% | 0.001 (0.002); 1.4% | -0.004 (0.001); 7.6% | -0.006 (0.001); 11.3% | -0.001 (0.002); 1.9% | -0.0002 (0.002); 0.4% |
|  | Poor entropy | 0.003 (0.003); 5.9% | 0.003 (0.002); 5.1% | -0.007 (0.002); 12.3% | -0.010 (0.001); 18.2% | -0.002 (0.002); 3.5% | 0.004 (0.003); 6.4% |
| Coverage | Good entropy | 94.3% (0.011) | 95.2% (0.010) | 94.3% (0.011) | 95.6% (0.010) | 95.4% (0.010) | 95.4% (0.010) |
|  | Medium entropy | 93.6% (0.011) | 93.4% (0.012) | 91.4% (0.013) | 95.4% (0.010) | 95.2% (0.010) | 95.8% (0.009) |
|  | Poor entropy | 93.0% (0.013) | 93.3% (0.012) | 88.4% (0.016) | 93.7% (0.012) | 93.7% (0.012) | 98.1% (0.007) |
| Bias-eliminated coverage | Good entropy | 93.3% (0.012) | 94.3% (0.011) | 94.3% (0.011) | 96.5% (0.009) | 94.8% (0.010) | 94.6% (0.010) |
|  | Medium entropy | 92.8% (0.012) | 92.8% (0.012) | 91.9% (0.013) | 97.8% (0.007) | 95.6% (0.010) | 95.8% (0.009) |
|  | Poor entropy | 92.8% (0.013) | 93.0% (0.013) | 92.0% (0.013) | 98.3% (0.006) | 94.2% (0.011) | 97.1% (0.008) |
| Empirical standard error | Good entropy | 0.037 (0.001) | 0.036 (0.002) | 0.032 (0.001) | 0.030 (0.001) | 0.033 (0.001) | 0.035 (0.001) |
|  | Medium entropy | 0.045 (0.001) | 0.041 (0.001) | 0.032 (0.001) | 0.028 (0.001) | 0.034 (0.001) | 0.038 (0.001) |
|  | Poor entropy | 0.054 (0.002) | 0.050 (0.002) | 0.032 (0.001) | 0.026 (0.001) | 0.035 (0.001) | 0.055 (0.002) |
| Average model standard error | Good entropy | 0.039 (0.001) | 0.037 (0.001) | 0.032 (0.0004) | 0.034 (0.0004) | 0.036 (0.0005) | 0.039 (0.001) |
|  | Medium entropy | 0.046 (0.001) | 0.041 (0.001) | 0.032 (0.001) | 0.034 (0.001) | 0.038 (0.001) | 0.045 (0.001) |
|  | Poor entropy | 0.066 (0.003) | 0.051 (0.001) | 0.032 (0.001) | 0.036 (0.001) | 0.043 (0.001) | 0.060 (0.002) |

**Supplement 6 Table 1c -** Performance measures (Monte Carlo standard errors) for each method and data generating mechanism for pure natural direct effect (PNDE) of Early-Onset Persistent versus Low conduct problems (true value = 0.300); *N* = 460 simulated datasets with good entropy (0.9), *N* = 456 simulated datasets with medium entropy (0.8), *N* = 415 simulated datasets with poor entropy (0.7)

| Performance measure (for θ) | Data-generating mechanism | One-step | Bias-adjusted three-step (BCH) | Modal class assignment | Non-inclusive pseudo class draws | Inclusive pseudo class draws | Updated pseudo class draws |
| --- | --- | --- | --- | --- | --- | --- | --- |
| % Bias | Good entropy | 0.002 (0.005); 0.8% | -0.004 (0.005); 1.3% | -0.021 (0.005); 6.9% | -0.024 (0.005); 8.1% | 0.004 (0.005); 1.4% | -0.004 (0.005); 1.3% |
|  | Medium entropy | 0.001 (0.006); 0.4% | -0.010 (0.006); 3.3% | -0.044 (0.005); 14.6% | -0.055 (0.004); 18.4% | 0.004 (0.006); 1.2% | -0.012 (0.006); 3.9% |
|  | Poor entropy | 0.001 (0.007); 0.3% | -0.024 (0.007); 7.9% | -0.074 (0.005); 24.5% | -0.091 (0.005); 30.4% | 0.012 (0.008); 3.9% | -0.026 (0.008); 8.6% |
| Coverage | Good entropy | 94.1% (0.011) | 93.3% (0.012) | 93.0% (0.012) | 95.0% (0.010) | 91.3% (0.013) | 94.6% (0.011) |
|  | Medium entropy | 93.6% (0.011) | 92.5% (0.012) | 90.4% (0.014) | 96.3% (0.009) | 90.8% (0.014) | 95.6% (0.010) |
|  | Poor entropy | 95.2% (0.011) | 92.5% (0.013) | 86.5% (0.017) | 93.0% (0.013) | 87.5% (0.016) | 96.9% (0.009) |
| Bias-eliminated coverage | Good entropy | 94.1% (0.011) | 93.0% (0.012) | 92.8% (0.012) | 97.0% (0.008) | 90.7% (0.014) | 94.8% (0.010) |
|  | Medium entropy | 93.9% (0.011) | 91.2% (0.013) | 91.2% (0.013) | 97.1% (0.008) | 90.6% (0.014) | 95.2% (0.010) |
|  | Poor entropy | 94.7% (0.011) | 94.2% (0.011) | 93.3% (0.012) | 98.6% (0.006) | 87.5% (0.016) | 95.9% (0.010) |
| Empirical standard error | Good entropy | 0.111 (0.004) | 0.111 (0.004) | 0.102 (0.003) | 0.097 (0.003) | 0.110 (0.004) | 0.107 (0.004) |
|  | Medium entropy | 0.126 (0.004) | 0.127 (0.004) | 0.106 (0.004) | 0.095 (0.003) | 0.124 (0.004) | 0.118 (0.004) |
|  | Poor entropy | 0.152 (0.005) | 0.151 (0.005) | 0.112 (0.004) | 0.092 (0.003) | 0.157 (0.005) | 0.155 (0.005) |
| Average model standard error | Good entropy | 0.108 (0.001) | 0.104 (0.001) | 0.096 (0.0004) | 0.102 (0.0004) | 0.101 (0.0005) | 0.108 (0.001) |
|  | Medium entropy | 0.126 (0.001) | 0.116 (0.001) | 0.097 (0.001) | 0.109 (0.001) | 0.108 (0.001) | 0.124 (0.001) |
|  | Poor entropy | 0.186 (0.012) | 0.137 (0.002) | 0.101 (0.001) | 0.119 (0.001) | 0.117 (0.001) | 0.173 (0.018) |

# **Supplementary References**

Bakk, Z., Tekle, F. B., & Vermunt, J. K. (2013). Estimating the Association between Latent Class Membership and External Variables Using Bias-adjusted Three-step Approaches. *Sociological Methodology*, *43*(1), 272–311.

Bray, B. C., Lanza, S. T., & Tan, X. (2015). Eliminating Bias in Classify-Analyze Approaches for Latent Class Analysis. *Structural Equation Modeling: A Multidisciplinary Journal*, *22*(1), 1–11.

Heinze, G., & Schemper, M. (2002). A solution to the problem of separation in logistic regression. *Statistics in Medicine*, *21*(16), 2409–2419.

Madley-Dowd, P., Hughes, R., Tilling, K., & Heron, J. (2019). The proportion of missing data should not be used to guide decisions on multiple imputation. *Journal of Clinical Epidemiology*, *110*, 63–73.

McLarnon, M. J. W., & O’Neill, T. A. (2018). Extensions of Auxiliary Variable Approaches for the Investigation of Mediation, Moderation, and Conditional Effects in Mixture Models. *Organizational Research Methods*, *21*(4), 955–982.

Muthén, B. (2011). *Applications of Causally Deﬁned Direct and Indirect Eﬀects in Mediation Analysis using SEM in Mplus*.

Muthén, B. O., Muthén, L. K., & Asparouhov, T. (2017). *Regression and mediation analysis using Mplus*. Muthén & Muthén.

Nguyen, T. Q., Schmid, I., & Stuart, E. A. (2021). Clarifying causal mediation analysis for the applied researcher: Defining effects based on what we want to learn. *Psychological Methods*, *26*(2), 255–271.

Nylund-Gibson, K., & Choi, A. Y. (2018). Ten frequently asked questions about latent class analysis. *Translational Issues in Psychological Science*, *4*(4), 440–461.

Pearl, J. (2012). The Causal Mediation Formula—A Guide to the Assessment of Pathways and Mechanisms. *Prevention Science*, *13*(4), 426–436.

VanderWeele, T. J. (2015). *Explanation in causal inference: Methods for mediation and interaction*. Oxford University Press.

Vermunt, J. K. (2010). Latent Class Modeling with Covariates: Two Improved Three-Step Approaches. *Political Analysis*, *18*(4), 450–469.

White, I. R., Royston, P., & Wood, A. M. (2011). Multiple imputation using chained equations: Issues and guidance for practice. *Statistics in Medicine*, *30*(4), 377–399.
