# Supplementary material for: Counterfactual Mediation Analysis with a Latent Class Exposure
Source: Multivariate Behav Res. 2024 May 31;59(4):818–40. doi: 10.1080/00273171.2024.2335394 (PMC11286213; doi:10.1080/00273171.2024.2335394)
Supplement: Supplemental Material [file HMBR_A_2335394_SM3655.zip › upcd_for_sim1_poor_entropy (1).html]

Updated Pseudo Class Draws (uPCD)


Code 

- Show All Code
- Hide All Code

# Updated Pseudo Class Draws (uPCD)

# 1 Introduction

R script for using uPCD to relate a latent class exposure to distal outcomes within a counterfactual mediation model.

Additional files needed to run this script (available here: https://anonymous.4open.science/r/latentclass-mediation-66B8):  
- Mplus data file: sim1.dat (first .dat file generated using Mplus input file: “1a poor entropy sim data.inp”)  
- Mplus input files: “2b uncond latent class.inp”, “2i pcd mediation.inp”

Using *Mplus v8.8* (display order of results in Mplus output files can depend on version).

## 1.1 Formulas

**FORMULA 1:**

\[P(Y=1 | X=0, M) = expit(\beta\_{0} + \beta\_{1}M)\]

**FORMULA 2:** \[P(M=1 | X=0) = expit(\alpha\_{0})\] **FORMULA 3:**

\[P(Y=1 | X, M) = expit(\beta\_{0} + \beta\_{1}M + \beta\_{2}X\_{1} + \beta\_{3}X\_{2} + \beta\_{4}X\_{3} + \beta\_{5}X\_{1}M + \beta\_{6}X\_{2}M + \beta\_{7}X\_{3}M )\] **FORMULA 4:**

\[P(Y=1 | X, M) = expit(\beta\_{0} + \beta\_{1}M + \beta\_{2}X\_{1} + \beta\_{3}X\_{2} + \beta\_{4}X\_{3} )\]

**FORMULA 5:**

\[P(M=1 | X) = expit(\alpha\_{0} + \alpha\_{1}X\_{1} + \alpha\_{2}X\_{2} + \alpha\_{3}X\_{3} )\]

## 1.2 Install and load packages

```
#install the required packages (if not already installed)
list.of.packages <- c("MplusAutomation", "logistf", "mnormt", "gdata")
new.packages <- list.of.packages[!(list.of.packages %in% installed.packages()[,"Package"])] 
    if(length(new.packages)) install.packages(new.packages)

#call all required packages
lapply(list.of.packages, require, character.only = TRUE)
```

```
## [[1]]
## [1] TRUE
## 
## [[2]]
## [1] TRUE
## 
## [[3]]
## [1] TRUE
## 
## [[4]]
## [1] TRUE
```

```
rm(list.of.packages, new.packages)

#check versions of required packages
getNamespaceVersion("MplusAutomation") #1.1.0
```

```
## version 
## "1.1.0"
```

```
getNamespaceVersion("logistf") #1.24.1
```

```
##  version 
## "1.24.1"
```

```
getNamespaceVersion("mnormt") #2.0.2
```

```
## version 
## "2.0.2"
```

```
getNamespaceVersion("gdata") #2.18.0.1
```

```
##    version 
## "2.18.0.1"
```

---

# 2 Script

Script to run uPCD using first simulated dataset with poor entropy.

## 2.1 Define variables, sample size, and output

```
#X = 4 class latent nominal exposure (conduct trajectories: early onset persistent, adolescent onset, childhood limited and low)
latent.classes <- 4
#M = binary mediator (peer deviance)
#Y = binary outcome (problematic alcohol use)
#U1-U5 = 5 binary latent class indicators (conduct problems from age 4 to 13 years)
indicators <- 5

#here we are just running 1 simulated dataset
sims <- 1

#simulated data has a sample size of 5000
sample <- 5000

#we wish to compare each latent class with all the others 
class.comp <- 4*3

#we wish to report 3 mediation effects (total effect - tot, total natural indirect effect - tie, and pure natural direct effect - pde) and their standard errors
mediation.effects <- 3*2
```

---

## 2.2 Prepare matrices

```
#create a matrix to store the mediation results for uPCD
#number of rows = number of simulated datasets (here we only use 1)
#number of columns is based on number of class comparisons (12), number of mediation effects (total, tie and pde) and SEs (6) and 
#number of extra parameters (e.g. flags for potential issues (6) and class probabilities (4))

pcd.all <- matrix(NA,sims,(class.comp*mediation.effects+6+latent.classes))

#labels represent the mediation effects (total, tie and pde) for each class comparison, flags for potential issues and class probabilities 
colnames(pcd.all) <-  
  c("tot.1v4","tot.1v3","tot.1v2","tot.2v4","tot.2v3","tot.2v1","tot.3v4","tot.3v2","tot.3v1","tot.4v3","tot.4v2","tot.4v1",
    "tie.1v4","tie.1v3","tie.1v2","tie.2v4","tie.2v3","tie.2v1","tie.3v4","tie.3v2","tie.3v1","tie.4v3","tie.4v2","tie.4v1",
    "pde.1v4","pde.1v3","pde.1v2","pde.2v4","pde.2v3","pde.2v1","pde.3v4","pde.3v2","pde.3v1","pde.4v3","pde.4v2","pde.4v1",
    "tot.1v4.se","tot.1v3.se","tot.1v2.se","tot.2v4.se","tot.2v3.se","tot.2v1.se","tot.3v4.se","tot.3v2.se","tot.3v1.se","tot.4v3.se","tot.4v2.se","tot.4v1.se","tie.1v4.se","tie.1v3.se","tie.1v2.se","tie.2v4.se","tie.2v3.se","tie.2v1.se","tie.3v4.se","tie.3v2.se","tie.3v1.se","tie.4v3.se","tie.4v2.se","tie.4v1.se","pde.1v4.se","pde.1v3.se","pde.1v2.se","pde.2v4.se","pde.2v3.se","pde.2v1.se","pde.3v4.se","pde.3v2.se","pde.3v1.se","pde.4v3.se","pde.4v2.se","pde.4v1.se",
    "fixed.th","large.th","largest.th","largest.th.se","zero.cell","zero.cell.imp", #flags for potential issues
    "p1","p2","p3","p4") #class probabilities

#create matrix to store the entropy of the unconditional model
entropy <- matrix(NA,sims,1)

#create matrix to store the area under the trajectory for each class in the unconditional model (this allows us to know the order of the classes)
auc <- matrix(NA,sims,latent.classes)
colnames(auc) <- c("auc1","auc2","auc3","auc4")
```

---

## 2.3 Read in the data

```
#instead of running loop over 500 simulated datasets, we will just run this code once for first simulated dataset (sim1.dat)
i <-1

#read in Mplus .dat file with simulated data (sim1.dat)
data.original <- read.table(file=paste0("sim", i, ".dat"), sep="", header=FALSE, col.names = 
                 c("y", #outcome
                   "u1","u2","u3","u4","u5", #latent class indicators
                   "m", #mediator
                   "c" #modal class assignment for the exposure
                   ))

head(data.original)
```

```
#replicate the original data and add empty columns to store imputed latent class membership for 4 classes (once generated) 
#and interactions between each latent class and the mediator
all.data <- data.original
all.data$x1 <- NA #empty column to add dummy code for membership in latent class 1
all.data$x2 <- NA #empty column to add dummy code for membership in latent class 2
all.data$x3 <- NA #empty column to add dummy code for membership in latent class 3
all.data$x4 <- NA #empty column to add dummy code for membership in latent class 4
all.data$int1 <- NA  #empty column to add interaction between latent class 1 and mediator
all.data$int2 <- NA  #empty column to add interaction between latent class 2 and mediator
all.data$int3 <- NA  #empty column to add interaction between latent class 3 and mediator
all.data$int4 <- NA  #empty column to add interaction between latent class 4 and mediator

head(all.data)
```

---

## 2.4 Unconditional latent class model

### 2.4.1 Run the model in Mplus and save the parameters

```
#first, we will simply rename the mplus .dat file and save it in the folder with the mplus input files
#this step is important when running many simulations, but not necessary otherwise
#sim.dat will be written over each time a new simulated dataset is analysed
prepareMplusData(data.original,"sim.dat")
```

```
## TITLE: Your title goes here
## DATA: FILE = "sim.dat";
## VARIABLE: 
## NAMES = y u1 u2 u3 u4 u5 m c; 
## MISSING=.;
```

```
#run the unconditional latent class model
runModels("2b uncond latent class.inp")

#read in the mplus output file and name it "model_output" to use later
model_output <- readModels("2b uncond latent class.out")

#save parameters from unconditional latent class model (within-class thresholds for latent class indicators and class intercepts) and their SEs
coef.se.original <- model_output$parameters$`unstandardized`[1:(indicators*latent.classes+(latent.classes-1)),3:4] 

#labels represent five within-class thresholds across 4 classes and 3 class intercepts)
rownames(coef.se.original) <- 
  c("th1.c1","th2.c1","th3.c1","th4.c1","th5.c1", #within-class thresholds for class 1
    "th1.c2","th2.c2","th3.c2","th4.c2","th5.c2", #within-class thresholds for class 2
    "th1.c3","th2.c3","th3.c3","th4.c3","th5.c3", #within-class thresholds for class 3
    "th1.c4","th2.c4","th3.c4","th4.c4","th5.c4", #within-class thresholds for class 4
    "int.c1","int.c2","int.c3") #latent class intercepts

coef.se.original
```

```
#save model entropy into the matrix we created at the start of the script
entropy[i,1] <- model_output$summaries$`Entropy`

entropy
```

```
##      [,1]
## [1,] 0.69
```

### 2.4.2 Calculate the class probabilities

```
#create a matrix of class probabilities
p.original <- matrix(NA,latent.classes,1)

#these can be calculated using the 3 class intercepts we saved in "coef.se.original"
p.original[1,1] <- exp(coef.se.original["int.c1","est"])/(1 + exp(coef.se.original["int.c1","est"]) + exp(coef.se.original["int.c2","est"]) + exp(coef.se.original["int.c3","est"]))
p.original[2,1] <- exp(coef.se.original["int.c2","est"])/(1 + exp(coef.se.original["int.c1","est"]) + exp(coef.se.original["int.c2","est"]) + exp(coef.se.original["int.c3","est"]))
p.original[3,1] <- exp(coef.se.original["int.c3","est"])/(1 + exp(coef.se.original["int.c1","est"]) + exp(coef.se.original["int.c2","est"]) + exp(coef.se.original["int.c3","est"]))  
p.original[4,1] <- 1/(1 + exp(coef.se.original["int.c1","est"]) + exp(coef.se.original["int.c2","est"]) + exp(coef.se.original["int.c3","est"]))

p.original
```

```
##            [,1]
## [1,] 0.09408812
## [2,] 0.09112496
## [3,] 0.11747573
## [4,] 0.69731119
```

### 2.4.3 Calculate the area under the trajectory

```
#derive area under the trajectory parameters so we know the order of the latent classes in the unconditional model
#these can be calculated using the within-class thresholds we saved in "coef.se.original"
auc[i,"auc1"] <- exp(-1*coef.se.original["th1.c1","est"])/(1+exp(-1*coef.se.original["th1.c1","est"]))+2*exp(-1*coef.se.original["th2.c1","est"])/(1+exp(-1*coef.se.original["th2.c1","est"]))+3*exp(-1*coef.se.original["th3.c1","est"])/(1+exp(-1*coef.se.original["th3.c1","est"]))+4*exp(-1*coef.se.original["th4.c1","est"])/(1+exp(-1*coef.se.original["th4.c1","est"]))+5*exp(-1*coef.se.original["th5.c1","est"])/(1+exp(-1*coef.se.original["th5.c1","est"]))
auc[i,"auc2"] <- exp(-1*coef.se.original["th1.c2","est"])/(1+exp(-1*coef.se.original["th1.c2","est"]))+2*exp(-1*coef.se.original["th2.c2","est"])/(1+exp(-1*coef.se.original["th2.c2","est"]))+3*exp(-1*coef.se.original["th3.c2","est"])/(1+exp(-1*coef.se.original["th3.c2","est"]))+4*exp(-1*coef.se.original["th4.c2","est"])/(1+exp(-1*coef.se.original["th4.c2","est"]))+5*exp(-1*coef.se.original["th5.c2","est"])/(1+exp(-1*coef.se.original["th5.c2","est"]))
auc[i,"auc3"] <- exp(-1*coef.se.original["th1.c3","est"])/(1+exp(-1*coef.se.original["th1.c3","est"]))+2*exp(-1*coef.se.original["th2.c3","est"])/(1+exp(-1*coef.se.original["th2.c3","est"]))+3*exp(-1*coef.se.original["th3.c3","est"])/(1+exp(-1*coef.se.original["th3.c3","est"]))+4*exp(-1*coef.se.original["th4.c3","est"])/(1+exp(-1*coef.se.original["th4.c3","est"]))+5*exp(-1*coef.se.original["th5.c3","est"])/(1+exp(-1*coef.se.original["th5.c3","est"]))
auc[i,"auc4"] <- exp(-1*coef.se.original["th1.c4","est"])/(1+exp(-1*coef.se.original["th1.c4","est"]))+2*exp(-1*coef.se.original["th2.c4","est"])/(1+exp(-1*coef.se.original["th2.c4","est"]))+3*exp(-1*coef.se.original["th3.c4","est"])/(1+exp(-1*coef.se.original["th3.c4","est"]))+4*exp(-1*coef.se.original["th4.c4","est"])/(1+exp(-1*coef.se.original["th4.c4","est"]))+5*exp(-1*coef.se.original["th5.c4","est"])/(1+exp(-1*coef.se.original["th5.c4","est"]))

#from the area under the trajectory parameters (auc) we can see that the order of the classes is: EOP, AO, CL and Low
auc
```

```
##         auc1     auc2     auc3     auc4
## [1,] 12.6152 8.829332 5.665677 2.646156
```

### 2.4.4 Save the (co)variance matrix for the parameters

We need the (co)variance matrix of the parameters in the unconditional latent class model in order to perturb these parameters and account for their uncertainty in the subsequent analyses. This covariance matrix is saved in the Mplus technical 3 output. Unfortunately, the order of the parameters in this covariance matrix is different to the order of the parameters in the Mplus main output (and therefore in “coef.se.original” saved above). This will be addressed later in the script when the parameters are perturbed. This will only be the case when there is no missing data in the latent class indicators (as is the case here given data are simulated). When there is missing data, the order of parameters in the covariance matrix (technical 3 output) is the same as the order in the main output.

```
#import tech3 as a covariance matrix to allow us to use the (co)variance between parameters for perturbing parameters
#this outputs paramCov as a matrix object in R 
tech3 <- model_output$tech3$paramCov

#this creates a full, symmetrical covariance matrix
upperTriangle(tech3) <- lowerTriangle(tech3, byrow=TRUE)
cov <- tech3

#address any fixed parameters: within class thresholds that have been fixed at 15 or -15 (representing a probability of 0 or 100%) do not have (co)variances
#the code below means that fixed parameters will still get perturbed but only a very small amount
#replace missing (999) in covariance matrix with 0 to represent no covariance for the fixed parameters
cov[cov==999] <- 0

#change the variance for fixed parameters to be very small (0.000000001)
for(j in 1:(indicators*latent.classes+(latent.classes-1))) {
  if (cov[j,j]==0) cov[j,j]<- 0.000000001}
```

---

## 2.5 Updated PCD

```
#set seed for uPCD
set.seed(82)
```

### 2.5.1 Step 1

In Step 1 we will initialise beta by fitting the logistic regression model for Y with beta for classes set to zero and initialise alpha by fitting the logistic regression model for M with alpha for classes set to zero. We will then perturb the estimates from these models with Gaussian noise of mean zero and values from the variance-covariance matrix of these parameter estimates.

```
#firth logistic regression is used to address problems with perfect prediction when bringing in XM interactions later in the script
#regress outcome Y on mediator M (see FORMULA 1)
model.y <- logistf(y~m, data=all.data)

summary(model.y)
```

```
## logistf(formula = y ~ m, data = all.data)
## 
## Model fitted by Penalized ML
## Coefficients:
##                   coef   se(coef) lower 0.95 upper 0.95    Chisq            p
## (Intercept) -0.6197009 0.03321114 -0.6850116 -0.5548145      Inf 0.000000e+00
## m            0.5665388 0.07101246  0.4273233  0.7057422 63.17913 1.887379e-15
##             method
## (Intercept)      2
## m                2
## 
## Method: 1-Wald, 2-Profile penalized log-likelihood, 3-None
## 
## Likelihood ratio test=63.20707 on 1 df, p=1.887379e-15, n=5000
## Wald test = 348.8917 on 1 df, p = 0
```

```
#regression model for mediator M (see FORMULA 2)
model.m <- logistf(m~1, data=all.data)

summary(model.m)
```

```
## logistf(formula = m ~ 1, data = all.data)
## 
## Model fitted by Penalized ML
## Coefficients:
##                  coef  se(coef) lower 0.95 upper 0.95 Chisq p method
## (Intercept) -1.367282 0.0351517          0          0   Inf 0      3
## 
## Method: 1-Wald, 2-Profile penalized log-likelihood, 3-None
## 
## Likelihood ratio test=0 on 0 df, p=1, n=5000
## Wald test = 1512.946 on 0 df, p = 0
```

```
#perturb the coefficients once around coefficients in model using variance-covariance matrix 
#initially use zero for exposure coefficients (and exposure-mediator interactions in model for Y)
#once latent class exposure has been imputed in subsequent runs, these will become coefficients from models
beta.y<-c(rmnorm(1,coef(model.y),vcov(model.y)),0,0,0,0,0,0)

beta.y
```

```
## (Intercept)           m                                                 
##  -0.6602031   0.6260792   0.0000000   0.0000000   0.0000000   0.0000000 
##                         
##   0.0000000   0.0000000
```

```
beta.m<-c(rmnorm(1,coef(model.m),vcov(model.m)),0,0,0)

beta.m
```

```
## (Intercept)                                     
##   -1.378899    0.000000    0.000000    0.000000
```

```
#we will create 80 imputed datasets for class membership 
#chosen to keep Monte Carlo error at less than 10% of standard error for parameters from regression model for Y
imp.n <- 80
#we will allow 20 iterations between saving out imputed class membership
cycles <- 20
#we will allow a burn in of 100 iterations before starting to save out imputed class membership
burnin <- 100

#create a matrix to save the results from every iteration (all results are recorded to assess convergence later)
#rows=iterations=(cycles*imp.n+burnin)
#columns=iteration number, coefficients from regression models, cell sizes and 2 flags for issues=(6+(latent.classes-1)*3+latent.classes*4)=31
results <- matrix(NA,cycles*imp.n+burnin,6+(latent.classes-1)*3+latent.classes*4) 

colnames(results) <- 
  c("iteration", #iteration number
    "b0.y","b1.y","b2.y","b3.y","b4.y","b5.y","b6.y","b7.y", #coefficients from regression model for Y
    "b0.m","b1.m","b2.m","b3.m", #coefficients from regression model for M
    "x1.m0.y0","x1.m1.y0","x2.m0.y0","x2.m1.y0","x3.m0.y0","x3.m1.y0","x4.m0.y0","x4.m1.y0",
    "x1.m0.y1","x1.m1.y1","x2.m0.y1","x2.m1.y1","x3.m0.y1","x3.m1.y1","x4.m0.y1","x4.m1.y1", #cell sizes from crosstabs for classes by mediator by outcome
    "zero.cell", #flag for presence of zero cells in crosstabs
    "large.th" #flag for a within-class threshold in unconditional latent class model that was out of bounds after perturbing
    ) 

#the first column is simply an indicator of iteration number (range from 1 to 1700)
results[,1]<-1:(cycles*imp.n+burnin)

#create a matrix to store cell sizes from crosstabs for classes by mediator by outcome 
xmy<-matrix(NA,1,latent.classes*4)
colnames(xmy) <-  c("x1m0y0","x1m1y0","x2m0y0","x2m1y0","x3m0y0","x3m1y0","x4m0y0","x4m1y0","x1m0y1","x1m1y1","x2m0y1","x2m1y1","x3m0y1","x3m1y1","x4m0y1","x4m1y1")

#create a matrix to store presence of zero cells in this crosstabs
zero.cell<-matrix(NA,1,1)

#create a matrix to store presence of a within-class threshold in unconditional latent class model that was out of bounds after perturbing (e.g., not corresponding to 0 to 100% probability) 
large.th<-matrix(NA,1,1)

#create a matrix to store imputed class membership for each person (sample*imp.n=5000*80)
imp <- matrix(NA,sample*imp.n,latent.classes+1)
colnames(imp) <- c("imp","x1","x2","x3","x4") 

#first column is simply an indicator for imputation number (range from 1 to 80)
for(h in 0:(imp.n-1)) {
  imp [c(sample*h+1:sample),1]<-h+1}
```

### 2.5.2 Steps 2 & 3

In Step 2, we will perturb the estimates from the latent class model (within-class thresholds and class intercepts) as previously explained and combine these along with the previous values of beta and alpha from the regression models for Y and M to calculate probabilities P(X=x|Y,M,U) for each class (x = 1,..k) for each subject. In Step 3, we will use these probabilities to randomly assign each subject to a class, and then fit the logistic regression model for Y and for M to obtain updated parameter estimates, which will again be perturbed. We will repeat steps 2 and 3 until convergence of beta and alpha. Here, we create a loop to repeat steps 2 and 3 below 1700 (imp.n\*cycles+burnin) times and save the imputed values of the latent class variable after every 20 iterations (after a 100 iteration burn in).

```
for(j in 1:(imp.n*cycles+burnin)) {
  results[j,2:9]<-beta.y #save perturbed beta coefficients from regression model for Y
  results[j,10:13]<-beta.m #save perturbed alpha coefficients from regression model for M    
  results[j,14:29]<-xmy #save cell sizes from crosstabs for classes by mediator by outcome
  results[j,30]<-zero.cell #save flag for presence of zero cells in this crosstabs
  results[j,31]<-large.th #save flag for a within-class threshold in unconditional latent class model that was out of bounds after perturbing
  
  #this saves each individual's imputed class membership 80 times (generated later in the script) into the matrix "imp" we created earlier     
  for(l in 1:imp.n) {
    if (j==(cycles*l+burnin)) imp[c(sample*(l-1)+1:sample),2:(latent.classes+1)]<-x
    } 

  #STEP 2a:
  #we will now perturb the parameters (within-class thresholds and class intercepts) that we saved earlier "coef.se.original" from the unconditional latent class model 
  #in order to perturb the parameters using the covariance matrix in tech3 we need to reorder parameters so they match with numbering in tech1
  #because we have no missing data, they are in a different order to what would be expected
  #when using a dataset with missing data on class indicators (as would usually be the case outside of simulated data), this reordering step is not needed
  
  #keep only the parameters (within-class thresholds for latent class indicators and class intercepts) from unconditional latent class model (i.e. drop the SEs)
  coef.mplus.orig<-coef.se.original[,"est"]
  #reorder the parameters so that they are in the same order as is used in the covariance matrix of parameters from tech3
  index<-c(1,5,9,13,17,2,6,10,14,18,3,7,11,15,19,4,8,12,16,20,21,22,23)
  coef.mplus.orig<-coef.mplus.orig[order(index)]
  #perturb these parameters based on their variance-covariance matrix (saved in "cov")
  coef.mplus<-rmnorm(1,coef.mplus.orig,cov)
  #reorder again to preserve original ordering
  index<-c(1,6,11,16,2,7,12,17,3,8,13,18,4,9,14,19,5,10,15,20,21,22,23)
  coef<-coef.mplus[order(index)]
  
  #when perturbing within-class thresholds, those with a large standard error can go out of bounds
  #e.g., corresponding to a probability that is not between 0 and 100%
  #we will create a flag so we know when this is the case
  large.th[1,1]<-0    
  for(k in 1:(latent.classes*indicators)) {
    if (coef[k]>15||coef[k]<(-15)) large.th[1,1] <- 1} 
  #we will also constrain within-class thresholds to be between -15 and 15 (corresponding to a probability that is between 0 and 100%)
  coef[coef>15] <- 15
  coef[coef<(-15)] <- (-15)
  
  #turning 'coef' into a matrix so can label rows and columns to use in script below (instead of referring to numbers)
  coef<-as.matrix(coef)
  rownames(coef) <- c("th1.c1","th2.c1","th3.c1","th4.c1","th5.c1",
                      "th1.c2","th2.c2","th3.c2","th4.c2","th5.c2",
                      "th1.c3","th2.c3","th3.c3","th4.c3","th5.c3",
                      "th1.c4","th2.c4","th3.c4","th4.c4","th5.c4",
                      "int.c1","int.c2","int.c3")
  colnames(coef) <- c("est")
  
  #now calculate P(X=x|U) - without perturbing this give us the probability of class membership (cprobs) from unconditional latent class model
  
  #for each class, multiply the individual data (responses to 5 binary indicators: U1 to U5) with within-class thresholds
  #class 1
  #this creates a matrix with within-class thresholds for class 1 repeated for every individual in the dataset (e.g. repeated 5000 times)
  theta <- matrix(rep(coef[(match("th1.c1",rownames(coef.se.original))):(match("th5.c1",rownames(coef.se.original))),"est"],each=nrow(all.data)),ncol=indicators,nrow=nrow(all.data))
  #if latent class indicator is present (-1)*threshold is used, if latent class indicator is absent (1)*threshold is used
  P1<-exp((-1)^all.data[,(match("u1",colnames(all.data))):(match("u5",colnames(all.data)))]*theta)/(1+exp((-1)^all.data[,(match("u1",colnames(all.data))):(match("u5",colnames(all.data)))]*theta))
  #class 2
  theta <- matrix(rep(coef[(match("th1.c2",rownames(coef.se.original))):(match("th5.c2",rownames(coef.se.original))),"est"],each=nrow(all.data)),ncol=indicators,nrow=nrow(all.data))
  P2<-exp((-1)^all.data[,(match("u1",colnames(all.data))):(match("u5",colnames(all.data)))]*theta)/(1+exp((-1)^all.data[,(match("u1",colnames(all.data))):(match("u5",colnames(all.data)))]*theta))
  #class 3
  theta <- matrix(rep(coef[(match("th1.c3",rownames(coef.se.original))):(match("th5.c3",rownames(coef.se.original))),"est"],each=nrow(all.data)),ncol=indicators,nrow=nrow(all.data))
  P3<-exp((-1)^all.data[,(match("u1",colnames(all.data))):(match("u5",colnames(all.data)))]*theta)/(1+exp((-1)^all.data[,(match("u1",colnames(all.data))):(match("u5",colnames(all.data)))]*theta))
  #class 4
  theta <- matrix(rep(coef[(match("th1.c4",rownames(coef.se.original))):(match("th5.c4",rownames(coef.se.original))),"est"],each=nrow(all.data)),ncol=indicators,nrow=nrow(all.data))
  P4<-exp((-1)^all.data[,(match("u1",colnames(all.data))):(match("u5",colnames(all.data)))]*theta)/(1+exp((-1)^all.data[,(match("u1",colnames(all.data))):(match("u5",colnames(all.data)))]*theta))
  
  #we need to create a new matrix of class probabilities using the perturbed class intercepts
  p <- matrix(NA,latent.classes,1)
  p[1,1] <- exp(coef["int.c1","est"])/(1 + exp(coef["int.c1","est"]) + exp(coef["int.c2","est"]) + exp(coef["int.c3","est"]))
  p[2,1] <- exp(coef["int.c2","est"])/(1 + exp(coef["int.c1","est"]) + exp(coef["int.c2","est"]) + exp(coef["int.c3","est"]))
  p[3,1] <- exp(coef["int.c3","est"])/(1 + exp(coef["int.c1","est"]) + exp(coef["int.c2","est"]) + exp(coef["int.c3","est"]))  
  p[4,1] <- 1/(1 + exp(coef["int.c1","est"]) + exp(coef["int.c2","est"]) + exp(coef["int.c3","est"]))
  
  #then multiply "P1" to "P4" by the perturbed class probabilities that we have created above "p" 
  #this gives us the probability of class membership for each person in the dataset
  #these will differ slightly to "cprobs" that can be exported from the unconditional latent class model due to perturbing
  N1<-apply(P1,1,prod)*p[1,1]
  N2<-apply(P2,1,prod)*p[2,1]
  N3<-apply(P3,1,prod)*p[3,1]
  N4<-apply(P4,1,prod)*p[4,1]    
  
  #STEP 2b - Bayes rule:
  
  #combine P(X=x|U) along with the coefficients from regression models for Y and M
  #this will calculate probabilities P(X=x|Y,M,U) for each class (x = 1,..k) for each individual
  #we will do this via a number a steps below
  
  #P(Y=1|M,X)
  #calculate probability that the outcome (Y) = 1 given mediator (M) and exposure (X, latent classes) 
  #this uses the coefficients from the regression model for Y 
  #intercept (b0), coef for M (b1), coefs for X (b2-b4) and coefs for XM interaction (b5-b7)
  #class 1
  num1 <- exp(results[j,"b0.y"]+results[j,"b1.y"]*all.data$m+results[j,"b2.y"]+results[j,"b5.y"]*all.data$m)
  Y11 <- num1/(1+num1)  
  #class 2
  num2 <- exp(results[j,"b0.y"]+results[j,"b1.y"]*all.data$m+results[j,"b3.y"]+results[j,"b6.y"]*all.data$m)
  Y21 <- num2/(1+num2)  
  #class 3
  num3 <- exp(results[j,"b0.y"]+results[j,"b1.y"]*all.data$m+results[j,"b4.y"]+results[j,"b7.y"]*all.data$m)
  Y31 <- num3/(1+num3)
  #class 4
  num4 <- exp(results[j,"b0.y"]+results[j,"b1.y"]*all.data$m)
  Y41 <- num4/(1+num4)
  
  #P(Y=0|M,X)
  #calculate probability that the outcome (Y) = 0 given mediator (M) and exposure (X, latent classes) 
  Y10 <- 1/(1+num1)
  Y20 <- 1/(1+num2)
  Y30 <- 1/(1+num3)
  Y40 <- 1/(1+num4)
  
  #P(M=1|X)
  #calculate probability that the mediator (M) = 1 given exposure (X, latent classes) 
  #this uses the coefficients from the regression model for M
  #intercept (b0) and coefs for X (b1-b3)
  #class 1 
  num1 <- exp(results[j,"b0.m"]+results[j,"b1.m"])
  M11 <- num1/(1+num1)
  #class 2
  num2 <- exp(results[j,"b0.m"]+results[j,"b2.m"])
  M21 <- num2/(1+num2) 
  #class 3
  num3 <- exp(results[j,"b0.m"]+results[j,"b3.m"])
  M31 <- num3/(1+num3)   
  #class 4
  num4 <- exp(results[j,"b0.m"])
  M41 <- num4/(1+num4) 
  
  #P(M=0|X)
  #calculate probability that the mediator (M) = 0 given exposure (X, latent classes)    
  M10 <- 1/(1+num1)
  M20 <- 1/(1+num2)
  M30 <- 1/(1+num3)      
  M40 <- 1/(1+num4)
  
  #P(X=x|Y=1,M=1,U)
  #calculate probability that exposure (X, latent classes) = 1 given outcome (Y) = 1, mediator (M) = 1, and latent class indicators (U)
  N111<-N1*Y11*M11
  #calculate probability that exposure (X, latent classes) = 2 given outcome (Y) = 1, mediator (M) = 1, and latent class indicators (U)
  N211<-N2*Y21*M21
  #calculate probability that exposure (X, latent classes) = 3 given outcome (Y) = 1, mediator (M) = 1, and latent class indicators (U)
  N311<-N3*Y31*M31
  #calculate probability that exposure (X, latent classes) = 4 given outcome (Y) = 1, mediator (M) = 1, and latent class indicators (U)
  N411<-N4*Y41*M41
  
  denom <- N111+N211+N311+N411
  Q111<-N111/denom
  Q211<-N211/denom
  Q311<-N311/denom
  Q411<-N411/denom 
  Q11<-cbind(Q111,Q211,Q311,Q411)
  head(Q11)
  
  #P(X=x|Y=0,M=0,U) 
  #calculate probability that exposure (X, latent classes) = 1 given outcome (Y) = 0, mediator (M) = 0, and latent class indicators (U)
  N100<-N1*Y10*M10
  #calculate probability that exposure (X, latent classes) = 2 given outcome (Y) = 0, mediator (M) = 0, and latent class indicators (U)
  N200<-N2*Y20*M30
  #calculate probability that exposure (X, latent classes) = 3 given outcome (Y) = 0, mediator (M) = 0, and latent class indicators (U)
  N300<-N3*Y30*M30
  #calculate probability that exposure (X, latent classes) = 4 given outcome (Y) = 0, mediator (M) = 0, and latent class indicators (U)
  N400<-N4*Y40*M40
  
  denom <- N100+N200+N300+N400   
  Q100<-N100/denom
  Q200<-N200/denom
  Q300<-N300/denom
  Q400<-N400/denom    
  Q00<-cbind(Q100,Q200,Q300,Q400)
  head(Q00)
  
  #P(X=x|Y=1,M=0,U)
  #calculate probability that exposure (X, latent classes) = 1 given outcome (Y) = 1, mediator (M) = 0, and latent class indicators (U)
  N101<-N1*Y11*M10
  #calculate probability that exposure (X, latent classes) = 2 given outcome (Y) = 1, mediator (M) = 0, and latent class indicators (U)
  N201<-N2*Y21*M30
  #calculate probability that exposure (X, latent classes) = 3 given outcome (Y) = 1, mediator (M) = 0, and latent class indicators (U)
  N301<-N3*Y31*M30
  #calculate probability that exposure (X, latent classes) = 4 given outcome (Y) = 1, mediator (M) = 0, and latent class indicators (U)
  N401<-N4*Y41*M40
  
  denom <- N101+N201+N301+N401     
  Q101<-N101/denom
  Q201<-N201/denom
  Q301<-N301/denom
  Q401<-N401/denom    
  Q01<-cbind(Q101,Q201,Q301,Q401)
  head(Q01)    
  
  #P(X=x|Y=0,M=1,U)
  #calculate probability that exposure (X, latent classes) = 1 given outcome (Y) = 0, mediator (M) = 1, and latent class indicators (U)
  N110<-N1*Y10*M11
  #calculate probability that exposure (X, latent classes) = 2 given outcome (Y) = 0, mediator (M) = 1, and latent class indicators (U)
  N210<-N2*Y20*M31
  #calculate probability that exposure (X, latent classes) = 3 given outcome (Y) = 0, mediator (M) = 1, and latent class indicators (U)
  N310<-N3*Y30*M31
  #calculate probability that exposure (X, latent classes) = 4 given outcome (Y) = 0, mediator (M) = 1, and latent class indicators (U)
  N410<-N4*Y40*M41
  
  denom <- N110+N210+N310+N410    
  Q110<-N110/denom
  Q210<-N210/denom
  Q310<-N310/denom
  Q410<-N410/denom   
  Q10<-cbind(Q110,Q210,Q310,Q410)
  head(Q10)   
  
  #derive the probability of class membership for each individual which takes into account the relationship between the classes, M and Y
  #use each individuals observed data on M and Y
  Q<-Q11
  #probabilities for those with M and Y absent
  Q[all.data$y==0 & all.data$m==0,]<-Q00[all.data$y==0 & all.data$m==0,]
  #probabilities for those with M absent and Y present
  Q[all.data$y==1 & all.data$m==0,]<-Q01[all.data$y==1 & all.data$m==0,]  
  #probabilities for those with M present and Y absent
  Q[all.data$y==0 & all.data$m==1,]<-Q10[all.data$y==0 & all.data$m==1,]
  #these probabilities will be used to impute class membership for each individual 
  head(Q)
  
  colnames(Q) <-  c("cprob1","cprob2","cprob3","cprob4")
  
  #STEP 3:
  
  #create a matrix to store class membership 
  x <- matrix(NA,sample,latent.classes)
  
  #now we will use the probabilities of class membership "Q" to randomly assign each individual to a class (X = 1,..k).
  #n=1 (number of random vectors to draw); size=1 per person
  for(k in 1:sample) {x[k,1:latent.classes] <- rmultinom(1,1,Q[k,])}
  
  #add imputed class membership to the data
  all.data[,"x1"] <- x[,1]
  all.data[,"x2"] <- x[,2]
  all.data[,"x3"] <- x[,3]
  all.data[,"x4"] <- x[,4]
  #add in XM interactions
  all.data[,"int1"] <- all.data[,"x1"]*all.data[,"m"]
  all.data[,"int2"] <- all.data[,"x2"]*all.data[,"m"]
  all.data[,"int3"] <- all.data[,"x3"]*all.data[,"m"]
  all.data[,"int4"] <- all.data[,"x4"]*all.data[,"m"]
  
  ###################################################
  #we will now perform some checks on the cell sizes from crosstabs for classes by mediator by outcome 
  #we will add these cell sizes into "results" to make traceplots to assess convergence
  
  #create a subset of the data for Y=0 
  no.y <- subset(all.data, all.data$y==0)
  #create a subset of the data for Y=1
  yes.y <- subset(all.data, all.data$y==1)
  
  #crosstabs for X1 and M for those with Y=0
  x1m.no.y<-table(no.y$x1,no.y$m)

  #this is to make sure matrix is 2 by 2 even when there are zero cells
  if (nrow(x1m.no.y)==1) x1m.no.y <- rbind(x1m.no.y,matrix(0,1,2))
  #crosstabs for X2 and M for those with Y=0 
  x2m.no.y<-table(no.y$x2,no.y$m)
  if (nrow(x2m.no.y)==1) x2m.no.y <- rbind(x2m.no.y,matrix(0,1,2))
  #crosstabs for X3 and M for those with Y=0 
  x3m.no.y<-table(no.y$x3,no.y$m)
  if (nrow(x3m.no.y)==1) x3m.no.y <- rbind(x3m.no.y,matrix(0,1,2))
  #crosstabs for X4 and M for those with Y=0 
  x4m.no.y<-table(no.y$x4,no.y$m)
  if (nrow(x4m.no.y)==1) x4m.no.y <- rbind(x4m.no.y,matrix(0,1,2))
  #crosstabs for X1 and M for those with Y=1 
  x1m.yes.y<-table(yes.y$x1,yes.y$m)
  if (nrow(x1m.yes.y)==1) x1m.yes.y <- rbind(x1m.yes.y,matrix(0,1,2))    
  x2m.yes.y<-table(yes.y$x2,yes.y$m)
  if (nrow(x2m.yes.y)==1) x2m.yes.y <- rbind(x2m.yes.y,matrix(0,1,2))    
  x3m.yes.y<-table(yes.y$x3,yes.y$m)
  if (nrow(x3m.yes.y)==1) x3m.yes.y <- rbind(x3m.yes.y,matrix(0,1,2))
  x4m.yes.y<-table(yes.y$x4,yes.y$m)
  if (nrow(x4m.yes.y)==1) x4m.yes.y <- rbind(x4m.yes.y,matrix(0,1,2))
  
  #use empty xmy matrix created earlier and fill in with cell sizes
  #cell size for X=1, M=0, Y=0
  xmy[1,"x1m0y0"] <- x1m.no.y[2,1]
  #cell size for X=1, M=1, Y=0  
  xmy[1,"x1m1y0"] <- x1m.no.y[2,2]
  #cell size for X=2, M=0, Y=0  
  xmy[1,"x2m0y0"] <- x2m.no.y[2,1]
  #cell size for X=2, M=1, Y=0  
  xmy[1,"x2m1y0"] <- x2m.no.y[2,2]
  #cell size for X=3, M=0, Y=0  
  xmy[1,"x3m0y0"] <- x3m.no.y[2,1]
  #cell size for X=3, M=1, Y=0  
  xmy[1,"x3m1y0"] <- x3m.no.y[2,2]
  #cell size for X=4, M=0, Y=0  
  xmy[1,"x4m0y0"] <- x4m.no.y[2,1]
  #cell size for X=4, M=1, Y=0  
  xmy[1,"x4m1y0"] <- x4m.no.y[2,2]
  #cell size for X=1, M=0, Y=1  
  xmy[1,"x1m0y1"] <- x1m.yes.y[2,1]
  #cell size for X=1, M=1, Y=1  
  xmy[1,"x1m1y1"] <- x1m.yes.y[2,2]
  #cell size for X=2, M=0, Y=1  
  xmy[1,"x2m0y1"] <- x2m.yes.y[2,1]
  #cell size for X=2, M=1, Y=1  
  xmy[1,"x2m1y1"] <- x2m.yes.y[2,2]
  #cell size for X=3, M=0, Y=1  
  xmy[1,"x3m0y1"] <- x3m.yes.y[2,1]
  #cell size for X=3, M=1, Y=1  
  xmy[1,"x3m1y1"] <- x3m.yes.y[2,2]
  #cell size for X=4, M=0, Y=1  
  xmy[1,"x4m0y1"] <- x4m.yes.y[2,1]
  #cell size for X=4, M=1, Y=1  
  xmy[1,"x4m1y1"] <- x4m.yes.y[2,2]
  
  #flag for number of zero cells in crosstabs for classes by mediator by outcome
  zero.cell[1,1] <- length(which(xmy == 0))
  ###################################################
  
  #fit the firth logistic regression model for P(Y|X,M) to obtain an updated parameter estimates now classes have been imputed (see FORMULA 3)
  if (zero.cell<2) model.y <- logistf(y~m+x1+x2+x3+int1+int2+int3, data=all.data)
  #if there is more than 1 zero cell, even firth logistic regression does not converge, therefore it is necessary to remove XM interactions from regression model (see FORMULA 4) 
  if (zero.cell>1) model.y <- logistf(y~m+x1+x2+x3, data=all.data)

  #fit the logistic regression model for P(M|X) to obtain updated parameter estimates (see FORMULA 5)
  model.m <- logistf(m~x1+x2+x3, data=all.data)

  #perturb the coefficients once around coefficients in model using variance-covariance matrix 
  if (zero.cell>1) beta.y<-c(rmnorm(1,coef(model.y),vcov(model.y)),0,0,0)
  if (zero.cell<2) beta.y<-c(rmnorm(1,coef(model.y),vcov(model.y))) 
  beta.m<-c(rmnorm(1,coef(model.m),vcov(model.m)))
  
} #end of iteration loop
```

---

# 3 Assessing convergence

Now we will create a pdf with trace plots of the parameters from the regression model for Y, the regression model for M, and the cell sizes in the cross-tabs between the exposure, mediator and outcome across the iterations. This is to detect issues with non-convergence. We will also create autocorrelation function (ACF) plots to check that 20 cycles of iterations is sufficient between saving the imputed latent class variable.

```
#create plot of parameters and cell sizes across each iteration
results.df <- as.data.frame(results)

#remove the first iteration when no data for X (latent classes) 
results.df <- subset(results.df, iteration>1)

#check autocorrelation for each parameter after removing burn in of 100 iterations
b2.y <- results.df[(burnin-1):((cycles*imp.n+burnin)-1),"b2.y"]
b3.y <- results.df[(burnin-1):((cycles*imp.n+burnin)-1),"b3.y"]
b4.y <- results.df[(burnin-1):((cycles*imp.n+burnin)-1),"b4.y"]
b5.y <- results.df[(burnin-1):((cycles*imp.n+burnin)-1),"b5.y"]
b6.y <- results.df[(burnin-1):((cycles*imp.n+burnin)-1),"b6.y"]
b7.y <- results.df[(burnin-1):((cycles*imp.n+burnin)-1),"b7.y"]
b1.m <- results.df[(burnin-1):((cycles*imp.n+burnin)-1),"b1.m"]
b2.m <- results.df[(burnin-1):((cycles*imp.n+burnin)-1),"b2.m"]
b3.m <- results.df[(burnin-1):((cycles*imp.n+burnin)-1),"b3.m"]

#create a pdf showing trace plots, histograms and autocorrelation plots for each parameter from the regression models 
#include trace plots and histograms for cell sizes

pdf(file=paste0("trace_plots", i, ".pdf")) 
plot(results.df$iteration, results.df$b2.y,
     xlab = "iteration",
     ylab = "b2.y (y on class 1 vs class 4)")
lines(results.df$iteration, results.df$b2.y)
hist (results.df$b2.y, xlab = "b2.y (y on class 1 vs class 4)")
acf(b2.y, lag.max=50)
plot(results.df$iteration, results.df$b3.y,
     xlab = "iteration",
     ylab = "b3.y (y on class 2 vs class 4)")
lines(results.df$iteration, results.df$b3.y)
hist (results.df$b3.y, xlab = "b3.y (y on class 2 vs class 4)")
acf(b3.y, lag.max=50)
plot(results.df$iteration, results.df$b4.y,
     xlab = "iteration",
     ylab = "b4.y (y on class 3 vs class 4)")
lines(results.df$iteration, results.df$b4.y)
hist (results.df$b4.y, xlab = "b4.y (y on class 3 vs class 4)")
acf(b4.y, lag.max=50)
plot(results.df$iteration, results.df$b5.y,
     xlab = "iteration",
     ylab = "b5.y (y on class 1 x m interaction)")
lines(results.df$iteration, results.df$b5.y)
hist (results.df$b5.y, xlab = "b5.y (y on class 1 x m interaction)")
acf(b5.y, lag.max=50)
plot(results.df$iteration, results.df$b6.y,
     xlab = "iteration",
     ylab = "b6.y (y on class 2 x m interaction)")
lines(results.df$iteration, results.df$b6.y)
hist (results.df$b6.y, xlab = "b6.y (y on class 2 x m interaction)")
acf(b6.y, lag.max=50)
plot(results.df$iteration, results.df$b7.y,
     xlab = "iteration",
     ylab = "b7.y (y on class 3 x m interaction)")
lines(results.df$iteration, results.df$b7.y)
hist (results.df$b7.y, xlab = "b7.y (y on class 3 x m interaction)")
acf(b7.y, lag.max=50)
plot(results.df$iteration, results.df$b1.m,
     xlab = "iteration",
     ylab = "b1.m (m on class 1 vs class 4)")
lines(results.df$iteration, results.df$b1.m)
hist (results.df$b1.m, xlab = "b1.m (m on class 1 vs class 4)")
acf(b1.m, lag.max=50)
plot(results.df$iteration, results.df$b2.m,
     xlab = "iteration",
     ylab = "b2.m (m on class 2 vs class 4)")
lines(results.df$iteration, results.df$b2.m)
hist (results.df$b2.m, xlab = "b2.m (m on class 2 vs class 4)")
acf(b2.m, lag.max=50)
plot(results.df$iteration, results.df$b3.m,
     xlab = "iteration",
     ylab = "b3.m (m on class 3 vs class 4")
lines(results.df$iteration, results.df$b3.m)
hist (results.df$b3.m, xlab = "b3.m (m on class 3 vs class 4)")
acf(b3.m, lag.max=50)
plot(results.df$iteration, results.df$x1.m0.y0,
     xlab = "iteration",
     ylab = "cell size x=1 m=0 y=0")
lines(results.df$iteration, results.df$x1.m0.y0)
hist (results.df$x1.m0.y0, xlab = "cell size x=1 m=0 y=0")
plot(results.df$iteration, results.df$x1.m1.y0,
     xlab = "iteration",
     ylab = "cell size x=1 m=1 y=0")
lines(results.df$iteration, results.df$x1.m1.y0)
hist (results.df$x1.m1.y0, xlab = "cell size x=1 m=1 y=0")
plot(results.df$iteration, results.df$x2.m0.y0,
     xlab = "iteration",
     ylab = "cell size x=2 m=0 y=0")
lines(results.df$iteration, results.df$x2.m0.y0)
hist (results.df$x2.m0.y0, xlab = "cell size x=2 m=0 y=0")
plot(results.df$iteration, results.df$x2.m1.y0,
     xlab = "iteration",
     ylab = "cell size x=2 m=1 y=0")
lines(results.df$iteration, results.df$x2.m1.y0)
hist (results.df$x2.m1.y0, xlab = "cell size x=2 m=1 y=0")
plot(results.df$iteration, results.df$x3.m0.y0,
     xlab = "iteration",
     ylab = "cell size x=3 m=0 y=0")
lines(results.df$iteration, results.df$x3.m0.y0)
hist (results.df$x3.m0.y0, xlab = "cell size x=3 m=0 y=0")
plot(results.df$iteration, results.df$x3.m1.y0,
     xlab = "iteration",
     ylab = "cell size x=3 m=1 y=0")
lines(results.df$iteration, results.df$x3.m1.y0)
hist (results.df$x3.m1.y0, xlab = "cell size x=3 m=1 y=0")
plot(results.df$iteration, results.df$x4.m0.y0,
     xlab = "iteration",
     ylab = "cell size x=4 m=0 y=0")
lines(results.df$iteration, results.df$x4.m0.y0)
hist (results.df$x4.m0.y0, xlab = "cell size x=4 m=0 y=0")
plot(results.df$iteration, results.df$x4.m1.y0,
     xlab = "iteration",
     ylab = "cell size x=4 m=1 y=0")
lines(results.df$iteration, results.df$x4.m1.y0)
hist (results.df$x4.m1.y0, xlab = "cell size x=4 m=1 y=0")
plot(results.df$iteration, results.df$x1.m0.y1,
     xlab = "iteration",
     ylab = "cell size x=1 m=0 y=1")
lines(results.df$iteration, results.df$x1.m0.y1)
hist (results.df$x1.m0.y1, xlab = "cell size x=1 m=0 y=1")
plot(results.df$iteration, results.df$x1.m1.y1,
     xlab = "iteration",
     ylab = "cell size x=1 m=1 y=1")
lines(results.df$iteration, results.df$x1.m1.y1)
hist (results.df$x1.m1.y1, xlab = "cell size x=1 m=1 y=1")
plot(results.df$iteration, results.df$x2.m0.y1,
     xlab = "iteration",
     ylab = "cell size x=2 m=0 y=1")
lines(results.df$iteration, results.df$x2.m0.y1)
hist (results.df$x2.m0.y1, xlab = "cell size x=2 m=0 y=1")
plot(results.df$iteration, results.df$x2.m1.y1,
     xlab = "iteration",
     ylab = "cell size x=2 m=1 y=1")
lines(results.df$iteration, results.df$x2.m1.y1)
hist (results.df$x2.m1.y1, xlab = "cell size x=2 m=1 y=1")
plot(results.df$iteration, results.df$x3.m0.y1,
     xlab = "iteration",
     ylab = "cell size x=3 m=0 y=1")
lines(results.df$iteration, results.df$x3.m0.y1)
hist (results.df$x3.m0.y1, xlab = "cell size x=3 m=0 y=1")
plot(results.df$iteration, results.df$x3.m1.y1,
     xlab = "iteration",
     ylab = "cell size x=3 m=1 y=1")
lines(results.df$iteration, results.df$x3.m1.y1)
hist (results.df$x3.m1.y1, xlab = "cell size x=3 m=1 y=1")
plot(results.df$iteration, results.df$x4.m0.y1,
     xlab = "iteration",
     ylab = "cell size x=4 m=0 y=1")
lines(results.df$iteration, results.df$x4.m0.y1)
hist (results.df$x4.m0.y1, xlab = "cell size x=4 m=0 y=1")
plot(results.df$iteration, results.df$x4.m1.y1,
     xlab = "iteration",
     ylab = "cell size x=4 m=1 y=1")
lines(results.df$iteration, results.df$x4.m1.y1)
hist (results.df$x4.m1.y1, xlab = "cell size x=4 m=1 y=1")
dev.off()
```

```
## png 
##   2
```

```
#showing the trace plot, histogram and acf plot for the beta coefficient for EOP versus Low in the regression model for Y estimated across 1,700 iterations
plot(results.df$iteration, results.df$b2.y,
     xlab = "iteration",
     ylab = "b2.y (y on class 1 vs class 4)")
lines(results.df$iteration, results.df$b2.y)
```

```
hist (results.df$b2.y, xlab = "b2.y (y on class 1 vs class 4)")
```

```
acf(b2.y, lag.max=50)
```

# 4 Analysis

Once we have examined the trace plots to check the parameters have converged, and examined the acf plots to check that 20 cycles of iterations is sufficient, we can proceed with the mediation analysis using the 80 imputed datasets. First, we will run the mediation model in Mplus and save the mediation effects for every possible class comparison.

```
#combine imputed class membership stored in "imp.n" with original data
#prepare 1 mplus .dat file for each imputed dataset to run mediation model (80 .dat files should be created)
for(l in 1:imp.n) {
  imp.subset <- cbind(data.original,subset(imp, imp[,1]==l))
  prepareMplusData(imp.subset, file=paste0("imp_", l, ".dat"))}
```

```
## TITLE: Your title goes here
## DATA: FILE = "imp_1.dat";
## VARIABLE: 
## NAMES = y u1 u2 u3 u4 u5 m c imp x1 x2 x3 x4; 
## MISSING=.;
## TITLE: Your title goes here
## DATA: FILE = "imp_2.dat";
## VARIABLE: 
## NAMES = y u1 u2 u3 u4 u5 m c imp x1 x2 x3 x4; 
## MISSING=.;
## TITLE: Your title goes here
## DATA: FILE = "imp_3.dat";
## VARIABLE: 
## NAMES = y u1 u2 u3 u4 u5 m c imp x1 x2 x3 x4; 
## MISSING=.;
## TITLE: Your title goes here
## DATA: FILE = "imp_4.dat";
## VARIABLE: 
## NAMES = y u1 u2 u3 u4 u5 m c imp x1 x2 x3 x4; 
## MISSING=.;
## TITLE: Your title goes here
## DATA: FILE = "imp_5.dat";
## VARIABLE: 
## NAMES = y u1 u2 u3 u4 u5 m c imp x1 x2 x3 x4; 
## MISSING=.;
## TITLE: Your title goes here
## DATA: FILE = "imp_6.dat";
## VARIABLE: 
## NAMES = y u1 u2 u3 u4 u5 m c imp x1 x2 x3 x4; 
## MISSING=.;
## TITLE: Your title goes here
## DATA: FILE = "imp_7.dat";
## VARIABLE: 
## NAMES = y u1 u2 u3 u4 u5 m c imp x1 x2 x3 x4; 
## MISSING=.;
## TITLE: Your title goes here
## DATA: FILE = "imp_8.dat";
## VARIABLE: 
## NAMES = y u1 u2 u3 u4 u5 m c imp x1 x2 x3 x4; 
## MISSING=.;
## TITLE: Your title goes here
## DATA: FILE = "imp_9.dat";
## VARIABLE: 
## NAMES = y u1 u2 u3 u4 u5 m c imp x1 x2 x3 x4; 
## MISSING=.;
## TITLE: Your title goes here
## DATA: FILE = "imp_10.dat";
## VARIABLE: 
## NAMES = y u1 u2 u3 u4 u5 m c imp x1 x2 x3 x4; 
## MISSING=.;
## TITLE: Your title goes here
## DATA: FILE = "imp_11.dat";
## VARIABLE: 
## NAMES = y u1 u2 u3 u4 u5 m c imp x1 x2 x3 x4; 
## MISSING=.;
## TITLE: Your title goes here
## DATA: FILE = "imp_12.dat";
## VARIABLE: 
## NAMES = y u1 u2 u3 u4 u5 m c imp x1 x2 x3 x4; 
## MISSING=.;
## TITLE: Your title goes here
## DATA: FILE = "imp_13.dat";
## VARIABLE: 
## NAMES = y u1 u2 u3 u4 u5 m c imp x1 x2 x3 x4; 
## MISSING=.;
## TITLE: Your title goes here
## DATA: FILE = "imp_14.dat";
## VARIABLE: 
## NAMES = y u1 u2 u3 u4 u5 m c imp x1 x2 x3 x4; 
## MISSING=.;
## TITLE: Your title goes here
## DATA: FILE = "imp_15.dat";
## VARIABLE: 
## NAMES = y u1 u2 u3 u4 u5 m c imp x1 x2 x3 x4; 
## MISSING=.;
## TITLE: Your title goes here
## DATA: FILE = "imp_16.dat";
## VARIABLE: 
## NAMES = y u1 u2 u3 u4 u5 m c imp x1 x2 x3 x4; 
## MISSING=.;
## TITLE: Your title goes here
## DATA: FILE = "imp_17.dat";
## VARIABLE: 
## NAMES = y u1 u2 u3 u4 u5 m c imp x1 x2 x3 x4; 
## MISSING=.;
## TITLE: Your title goes here
## DATA: FILE = "imp_18.dat";
## VARIABLE: 
## NAMES = y u1 u2 u3 u4 u5 m c imp x1 x2 x3 x4; 
## MISSING=.;
## TITLE: Your title goes here
## DATA: FILE = "imp_19.dat";
## VARIABLE: 
## NAMES = y u1 u2 u3 u4 u5 m c imp x1 x2 x3 x4; 
## MISSING=.;
## TITLE: Your title goes here
## DATA: FILE = "imp_20.dat";
## VARIABLE: 
## NAMES = y u1 u2 u3 u4 u5 m c imp x1 x2 x3 x4; 
## MISSING=.;
## TITLE: Your title goes here
## DATA: FILE = "imp_21.dat";
## VARIABLE: 
## NAMES = y u1 u2 u3 u4 u5 m c imp x1 x2 x3 x4; 
## MISSING=.;
## TITLE: Your title goes here
## DATA: FILE = "imp_22.dat";
## VARIABLE: 
## NAMES = y u1 u2 u3 u4 u5 m c imp x1 x2 x3 x4; 
## MISSING=.;
## TITLE: Your title goes here
## DATA: FILE = "imp_23.dat";
## VARIABLE: 
## NAMES = y u1 u2 u3 u4 u5 m c imp x1 x2 x3 x4; 
## MISSING=.;
## TITLE: Your title goes here
## DATA: FILE = "imp_24.dat";
## VARIABLE: 
## NAMES = y u1 u2 u3 u4 u5 m c imp x1 x2 x3 x4; 
## MISSING=.;
## TITLE: Your title goes here
## DATA: FILE = "imp_25.dat";
## VARIABLE: 
## NAMES = y u1 u2 u3 u4 u5 m c imp x1 x2 x3 x4; 
## MISSING=.;
## TITLE: Your title goes here
## DATA: FILE = "imp_26.dat";
## VARIABLE: 
## NAMES = y u1 u2 u3 u4 u5 m c imp x1 x2 x3 x4; 
## MISSING=.;
## TITLE: Your title goes here
## DATA: FILE = "imp_27.dat";
## VARIABLE: 
## NAMES = y u1 u2 u3 u4 u5 m c imp x1 x2 x3 x4; 
## MISSING=.;
## TITLE: Your title goes here
## DATA: FILE = "imp_28.dat";
## VARIABLE: 
## NAMES = y u1 u2 u3 u4 u5 m c imp x1 x2 x3 x4; 
## MISSING=.;
## TITLE: Your title goes here
## DATA: FILE = "imp_29.dat";
## VARIABLE: 
## NAMES = y u1 u2 u3 u4 u5 m c imp x1 x2 x3 x4; 
## MISSING=.;
## TITLE: Your title goes here
## DATA: FILE = "imp_30.dat";
## VARIABLE: 
## NAMES = y u1 u2 u3 u4 u5 m c imp x1 x2 x3 x4; 
## MISSING=.;
## TITLE: Your title goes here
## DATA: FILE = "imp_31.dat";
## VARIABLE: 
## NAMES = y u1 u2 u3 u4 u5 m c imp x1 x2 x3 x4; 
## MISSING=.;
## TITLE: Your title goes here
## DATA: FILE = "imp_32.dat";
## VARIABLE: 
## NAMES = y u1 u2 u3 u4 u5 m c imp x1 x2 x3 x4; 
## MISSING=.;
## TITLE: Your title goes here
## DATA: FILE = "imp_33.dat";
## VARIABLE: 
## NAMES = y u1 u2 u3 u4 u5 m c imp x1 x2 x3 x4; 
## MISSING=.;
## TITLE: Your title goes here
## DATA: FILE = "imp_34.dat";
## VARIABLE: 
## NAMES = y u1 u2 u3 u4 u5 m c imp x1 x2 x3 x4; 
## MISSING=.;
## TITLE: Your title goes here
## DATA: FILE = "imp_35.dat";
## VARIABLE: 
## NAMES = y u1 u2 u3 u4 u5 m c imp x1 x2 x3 x4; 
## MISSING=.;
## TITLE: Your title goes here
## DATA: FILE = "imp_36.dat";
## VARIABLE: 
## NAMES = y u1 u2 u3 u4 u5 m c imp x1 x2 x3 x4; 
## MISSING=.;
## TITLE: Your title goes here
## DATA: FILE = "imp_37.dat";
## VARIABLE: 
## NAMES = y u1 u2 u3 u4 u5 m c imp x1 x2 x3 x4; 
## MISSING=.;
## TITLE: Your title goes here
## DATA: FILE = "imp_38.dat";
## VARIABLE: 
## NAMES = y u1 u2 u3 u4 u5 m c imp x1 x2 x3 x4; 
## MISSING=.;
## TITLE: Your title goes here
## DATA: FILE = "imp_39.dat";
## VARIABLE: 
## NAMES = y u1 u2 u3 u4 u5 m c imp x1 x2 x3 x4; 
## MISSING=.;
## TITLE: Your title goes here
## DATA: FILE = "imp_40.dat";
## VARIABLE: 
## NAMES = y u1 u2 u3 u4 u5 m c imp x1 x2 x3 x4; 
## MISSING=.;
## TITLE: Your title goes here
## DATA: FILE = "imp_41.dat";
## VARIABLE: 
## NAMES = y u1 u2 u3 u4 u5 m c imp x1 x2 x3 x4; 
## MISSING=.;
## TITLE: Your title goes here
## DATA: FILE = "imp_42.dat";
## VARIABLE: 
## NAMES = y u1 u2 u3 u4 u5 m c imp x1 x2 x3 x4; 
## MISSING=.;
## TITLE: Your title goes here
## DATA: FILE = "imp_43.dat";
## VARIABLE: 
## NAMES = y u1 u2 u3 u4 u5 m c imp x1 x2 x3 x4; 
## MISSING=.;
## TITLE: Your title goes here
## DATA: FILE = "imp_44.dat";
## VARIABLE: 
## NAMES = y u1 u2 u3 u4 u5 m c imp x1 x2 x3 x4; 
## MISSING=.;
## TITLE: Your title goes here
## DATA: FILE = "imp_45.dat";
## VARIABLE: 
## NAMES = y u1 u2 u3 u4 u5 m c imp x1 x2 x3 x4; 
## MISSING=.;
## TITLE: Your title goes here
## DATA: FILE = "imp_46.dat";
## VARIABLE: 
## NAMES = y u1 u2 u3 u4 u5 m c imp x1 x2 x3 x4; 
## MISSING=.;
## TITLE: Your title goes here
## DATA: FILE = "imp_47.dat";
## VARIABLE: 
## NAMES = y u1 u2 u3 u4 u5 m c imp x1 x2 x3 x4; 
## MISSING=.;
## TITLE: Your title goes here
## DATA: FILE = "imp_48.dat";
## VARIABLE: 
## NAMES = y u1 u2 u3 u4 u5 m c imp x1 x2 x3 x4; 
## MISSING=.;
## TITLE: Your title goes here
## DATA: FILE = "imp_49.dat";
## VARIABLE: 
## NAMES = y u1 u2 u3 u4 u5 m c imp x1 x2 x3 x4; 
## MISSING=.;
## TITLE: Your title goes here
## DATA: FILE = "imp_50.dat";
## VARIABLE: 
## NAMES = y u1 u2 u3 u4 u5 m c imp x1 x2 x3 x4; 
## MISSING=.;
## TITLE: Your title goes here
## DATA: FILE = "imp_51.dat";
## VARIABLE: 
## NAMES = y u1 u2 u3 u4 u5 m c imp x1 x2 x3 x4; 
## MISSING=.;
## TITLE: Your title goes here
## DATA: FILE = "imp_52.dat";
## VARIABLE: 
## NAMES = y u1 u2 u3 u4 u5 m c imp x1 x2 x3 x4; 
## MISSING=.;
## TITLE: Your title goes here
## DATA: FILE = "imp_53.dat";
## VARIABLE: 
## NAMES = y u1 u2 u3 u4 u5 m c imp x1 x2 x3 x4; 
## MISSING=.;
## TITLE: Your title goes here
## DATA: FILE = "imp_54.dat";
## VARIABLE: 
## NAMES = y u1 u2 u3 u4 u5 m c imp x1 x2 x3 x4; 
## MISSING=.;
## TITLE: Your title goes here
## DATA: FILE = "imp_55.dat";
## VARIABLE: 
## NAMES = y u1 u2 u3 u4 u5 m c imp x1 x2 x3 x4; 
## MISSING=.;
## TITLE: Your title goes here
## DATA: FILE = "imp_56.dat";
## VARIABLE: 
## NAMES = y u1 u2 u3 u4 u5 m c imp x1 x2 x3 x4; 
## MISSING=.;
## TITLE: Your title goes here
## DATA: FILE = "imp_57.dat";
## VARIABLE: 
## NAMES = y u1 u2 u3 u4 u5 m c imp x1 x2 x3 x4; 
## MISSING=.;
## TITLE: Your title goes here
## DATA: FILE = "imp_58.dat";
## VARIABLE: 
## NAMES = y u1 u2 u3 u4 u5 m c imp x1 x2 x3 x4; 
## MISSING=.;
## TITLE: Your title goes here
## DATA: FILE = "imp_59.dat";
## VARIABLE: 
## NAMES = y u1 u2 u3 u4 u5 m c imp x1 x2 x3 x4; 
## MISSING=.;
## TITLE: Your title goes here
## DATA: FILE = "imp_60.dat";
## VARIABLE: 
## NAMES = y u1 u2 u3 u4 u5 m c imp x1 x2 x3 x4; 
## MISSING=.;
## TITLE: Your title goes here
## DATA: FILE = "imp_61.dat";
## VARIABLE: 
## NAMES = y u1 u2 u3 u4 u5 m c imp x1 x2 x3 x4; 
## MISSING=.;
## TITLE: Your title goes here
## DATA: FILE = "imp_62.dat";
## VARIABLE: 
## NAMES = y u1 u2 u3 u4 u5 m c imp x1 x2 x3 x4; 
## MISSING=.;
## TITLE: Your title goes here
## DATA: FILE = "imp_63.dat";
## VARIABLE: 
## NAMES = y u1 u2 u3 u4 u5 m c imp x1 x2 x3 x4; 
## MISSING=.;
## TITLE: Your title goes here
## DATA: FILE = "imp_64.dat";
## VARIABLE: 
## NAMES = y u1 u2 u3 u4 u5 m c imp x1 x2 x3 x4; 
## MISSING=.;
## TITLE: Your title goes here
## DATA: FILE = "imp_65.dat";
## VARIABLE: 
## NAMES = y u1 u2 u3 u4 u5 m c imp x1 x2 x3 x4; 
## MISSING=.;
## TITLE: Your title goes here
## DATA: FILE = "imp_66.dat";
## VARIABLE: 
## NAMES = y u1 u2 u3 u4 u5 m c imp x1 x2 x3 x4; 
## MISSING=.;
## TITLE: Your title goes here
## DATA: FILE = "imp_67.dat";
## VARIABLE: 
## NAMES = y u1 u2 u3 u4 u5 m c imp x1 x2 x3 x4; 
## MISSING=.;
## TITLE: Your title goes here
## DATA: FILE = "imp_68.dat";
## VARIABLE: 
## NAMES = y u1 u2 u3 u4 u5 m c imp x1 x2 x3 x4; 
## MISSING=.;
## TITLE: Your title goes here
## DATA: FILE = "imp_69.dat";
## VARIABLE: 
## NAMES = y u1 u2 u3 u4 u5 m c imp x1 x2 x3 x4; 
## MISSING=.;
## TITLE: Your title goes here
## DATA: FILE = "imp_70.dat";
## VARIABLE: 
## NAMES = y u1 u2 u3 u4 u5 m c imp x1 x2 x3 x4; 
## MISSING=.;
## TITLE: Your title goes here
## DATA: FILE = "imp_71.dat";
## VARIABLE: 
## NAMES = y u1 u2 u3 u4 u5 m c imp x1 x2 x3 x4; 
## MISSING=.;
## TITLE: Your title goes here
## DATA: FILE = "imp_72.dat";
## VARIABLE: 
## NAMES = y u1 u2 u3 u4 u5 m c imp x1 x2 x3 x4; 
## MISSING=.;
## TITLE: Your title goes here
## DATA: FILE = "imp_73.dat";
## VARIABLE: 
## NAMES = y u1 u2 u3 u4 u5 m c imp x1 x2 x3 x4; 
## MISSING=.;
## TITLE: Your title goes here
## DATA: FILE = "imp_74.dat";
## VARIABLE: 
## NAMES = y u1 u2 u3 u4 u5 m c imp x1 x2 x3 x4; 
## MISSING=.;
## TITLE: Your title goes here
## DATA: FILE = "imp_75.dat";
## VARIABLE: 
## NAMES = y u1 u2 u3 u4 u5 m c imp x1 x2 x3 x4; 
## MISSING=.;
## TITLE: Your title goes here
## DATA: FILE = "imp_76.dat";
## VARIABLE: 
## NAMES = y u1 u2 u3 u4 u5 m c imp x1 x2 x3 x4; 
## MISSING=.;
## TITLE: Your title goes here
## DATA: FILE = "imp_77.dat";
## VARIABLE: 
## NAMES = y u1 u2 u3 u4 u5 m c imp x1 x2 x3 x4; 
## MISSING=.;
## TITLE: Your title goes here
## DATA: FILE = "imp_78.dat";
## VARIABLE: 
## NAMES = y u1 u2 u3 u4 u5 m c imp x1 x2 x3 x4; 
## MISSING=.;
## TITLE: Your title goes here
## DATA: FILE = "imp_79.dat";
## VARIABLE: 
## NAMES = y u1 u2 u3 u4 u5 m c imp x1 x2 x3 x4; 
## MISSING=.;
## TITLE: Your title goes here
## DATA: FILE = "imp_80.dat";
## VARIABLE: 
## NAMES = y u1 u2 u3 u4 u5 m c imp x1 x2 x3 x4; 
## MISSING=.;
```

```
#create "imp.txt" file for Mplus to call imputed datasets
imp.txt <- matrix(NA,imp.n,1)
for(l in 1:imp.n) {
  imp.txt[l,1] <- paste0("imp_", l, ".dat")}
write.table(imp.txt, file="imp.txt", quote = FALSE, row.names = FALSE, col.names = FALSE)

#run mediation model using updated PCD
runModels("2i pcd mediation.inp")

#read in parameters from PCD mediation model 
est.pcd <- readModels("2i pcd mediation.out", what="parameters")$parameters$`unstandardized`

#store only the parameters (mediation effects and class probabilities) and their SEs in the matrix created at the start "upcd.all"
pcd.all[i,(match("tot.1v4",colnames(pcd.all))):(match("pde.4v1",colnames(pcd.all)))] <- est.pcd[100:135,"est"] # TOT_1V4 to PDE_4V1
pcd.all[i,(match("tot.1v4.se",colnames(pcd.all))):(match("pde.4v1.se",colnames(pcd.all)))] <- est.pcd[100:135,"se"] # TOT_1V4 to PDE_4V1
pcd.all[i,(match("p1",colnames(pcd.all))):(match("p4",colnames(pcd.all)))] <- est.pcd[72:75,"est"]  # P_X1 to P_X4

#flags for potential issues: "fixed.th","large.th","largest.th","largest.th.se","zero.cell","zero.cell.imp"

#flag if fixed threshold in unconditional model which results in missing value in tech3 covariance matrix of parameters
pcd.all[i,"fixed.th"] <- 0 #set flag to 0 to start
for(k in 1:(indicators*latent.classes+(latent.classes-1))*(indicators*latent.classes+(latent.classes-1))) { #size of tech3 matrix is 23x23 (529) 
  if (tech3[k]==999) pcd.all[i,"fixed.th"] <- 1}

#when perturbing within-class thresholds, those with a large standard error can go out of bounds (e.g., corresponding to a probability that is not between 0 and 100%)
#we created a flag for this in "results" so here we will move this into "pcd.all" and record whether this was the case in any iteration
pcd.all[i,"large.th"] <- 0 #set flag to 0 to start
results[1,"large.th"]<-0 #change row 1 in "results" to 0
for(k in 1:(cycles*imp.n+burnin)) {
  if (results[k,"large.th"]==1) pcd.all[i,"large.th"] <- 1}

#we also want a flag so that we know the largest standard error for a threshold in the unconditional model (and the threshold this SE corresponds to)
#this flag captures the largest SE
pcd.all[i,"largest.th.se"] <- max(coef.se.original[,"se"])
#this flag records the threshold that corresponds to largest SE
pcd.all[i,"largest.th"] <- coef.se.original[which.max(coef.se.original[,"se"]),"est"]

#we created a flag for number of zero cells in the crosstabs for classes by mediator by outcome in "results"
#we will move this flag to "pcd.all" and record the largest number of zero cells in any iteration
results[1,"zero.cell"]<-0 #change row 1 in "results" to 0 as latent classes not imputed in first iteration
pcd.all[i,"zero.cell"] <- max(results[,"zero.cell"])

#we will also flag if there was a zero cell (in the crosstabs for classes by mediator by outcome) in any of the iterations that we saved as one of the 80 imputed datasets
zero.cell.imp <- matrix(NA,1,imp.n)
for(l in 1:imp.n) {
  zero.cell.imp[1,l] <- results[cycles*l+burnin,"zero.cell"]}

pcd.all[i,"zero.cell.imp"] <- 0 #set flag to 0 to start
for(k in 1:imp.n) {
  if (zero.cell.imp[1,k]>0) pcd.all[1,"zero.cell.imp"] <- 1}

pcd.all
```

```
##      tot.1v4 tot.1v3 tot.1v2 tot.2v4 tot.2v3 tot.2v1 tot.3v4 tot.3v2 tot.3v1
## [1,]   0.417   0.307   0.015   0.401   0.292  -0.015    0.11  -0.292  -0.307
##      tot.4v3 tot.4v2 tot.4v1 tie.1v4 tie.1v3 tie.1v2 tie.2v4 tie.2v3 tie.2v1
## [1,]   -0.11  -0.401  -0.417   0.073   0.086   0.043   0.044   0.065  -0.068
##      tie.3v4 tie.3v2 tie.3v1 tie.4v3 tie.4v2 tie.4v1 pde.1v4 pde.1v3 pde.1v2
## [1,]  -0.011  -0.039  -0.079   0.004  -0.009  -0.022   0.343   0.221  -0.028
##      pde.2v4 pde.2v3 pde.2v1 pde.3v4 pde.3v2 pde.3v1 pde.4v3 pde.4v2 pde.4v1
## [1,]   0.357   0.227   0.052   0.121  -0.253  -0.228  -0.114  -0.392  -0.395
##      tot.1v4.se tot.1v3.se tot.1v2.se tot.2v4.se tot.2v3.se tot.2v1.se
## [1,]      0.077      0.113      0.119      0.104      0.132      0.119
##      tot.3v4.se tot.3v2.se tot.3v1.se tot.4v3.se tot.4v2.se tot.4v1.se
## [1,]      0.107      0.132      0.113      0.107      0.104      0.077
##      tie.1v4.se tie.1v3.se tie.1v2.se tie.2v4.se tie.2v3.se tie.2v1.se
## [1,]      0.028      0.036      0.031      0.029      0.037      0.048
##      tie.3v4.se tie.3v2.se tie.3v1.se tie.4v3.se tie.4v2.se tie.4v1.se
## [1,]      0.017       0.03      0.045      0.006      0.009      0.015
##      pde.1v4.se pde.1v3.se pde.1v2.se pde.2v4.se pde.2v3.se pde.2v1.se
## [1,]      0.082      0.121       0.12      0.111      0.135      0.118
##      pde.3v4.se pde.3v2.se pde.3v1.se pde.4v3.se pde.4v2.se pde.4v1.se fixed.th
## [1,]      0.105      0.131      0.121      0.105      0.105      0.079        0
##      large.th largest.th largest.th.se zero.cell zero.cell.imp    p1    p2
## [1,]        0     -2.603         2.181         1             0 0.096 0.101
##         p3    p4
## [1,] 0.116 0.687
```

Now we will save the mediation effects only for our class comparisons of interest (e.g. using Low class as the reference group).

```
#create a matrix to store the parameters of interest (mediation effects and class probabilities) and their SE
pcd <- matrix(NA,sims,(latent.classes-1)*mediation.effects+latent.classes)
colnames(pcd) <- 
  c("tot.eop","tot.ao","tot.cl","tie.eop","tie.ao","tie.cl","pde.eop","pde.ao","pde.cl", #mediation effects for EOP, AO and CL versus Low
    "tot.eop.se","tot.ao.se","tot.cl.se","tie.eop.se","tie.ao.se","tie.cl.se","pde.eop.se","pde.ao.se","pde.cl.se", # corresponding SE
    "p1","p2","p3","p4") #class probabilities

#move over class probabilities
pcd[,"p1"]<-pcd.all[,"p1"]
pcd[,"p2"]<-pcd.all[,"p2"]
pcd[,"p3"]<-pcd.all[,"p3"]
pcd[,"p4"]<-pcd.all[,"p4"]

#move over desired class comparisons
#this will differ depending on order of the classes in mplus output - determined using the area under the trajectory (auc)
#here we know that the class order is EOP, AO, CL and Low but we have included script for all possible orders below
for(i in 1:sims) {
  #1234 (eop,ao,cl,low) 
  if(auc[i,"auc1"]>auc[i,"auc2"] & auc[i,"auc1"]>auc[i,"auc3"] & auc[i,"auc1"]>auc[i,"auc4"]
     & auc[i,"auc2"]>auc[i,"auc3"] & auc[i,"auc2"]>auc[i,"auc4"] & auc[i,"auc3"]>auc[i,"auc4"])
    pcd[i,1:18] <- pcd.all[i,c(1,4,7,13,16,19,25,28,31,37,40,43,49,52,55,61,64,67)]
  #1243 (eop,ao,low,cl)
  if(auc[i,"auc1"]>auc[i,"auc2"] & auc[i,"auc1"]>auc[i,"auc3"] & auc[i,"auc1"]>auc[i,"auc4"]
     & auc[i,"auc2"]>auc[i,"auc3"] & auc[i,"auc2"]>auc[i,"auc4"] & auc[i,"auc4"]>auc[i,"auc3"])
    pcd[i,1:18] <- pcd.all[i,c(2,5,10,14,17,22,26,29,34,38,41,46,50,53,58,62,65,70)]
  #1324 (eop,cl,ao,low) 
  if(auc[i,"auc1"]>auc[i,"auc2"] & auc[i,"auc1"]>auc[i,"auc3"] & auc[i,"auc1"]>auc[i,"auc4"]
     & auc[i,"auc3"]>auc[i,"auc2"] & auc[i,"auc3"]>auc[i,"auc4"] & auc[i,"auc2"]>auc[i,"auc4"])
    pcd[i,1:18] <- pcd.all[i,c(1,7,4,13,19,16,25,31,28,37,43,40,49,55,52,61,67,64)]
  #1342 (eop,cl,low,ao)
  if(auc[i,"auc1"]>auc[i,"auc2"] & auc[i,"auc1"]>auc[i,"auc3"] & auc[i,"auc1"]>auc[i,"auc4"]
     & auc[i,"auc4"]>auc[i,"auc3"] & auc[i,"auc4"]>auc[i,"auc2"] & auc[i,"auc2"]>auc[i,"auc3"])
    pcd[i,1:18] <- pcd.all[i,c(2,10,5,14,22,17,26,34,29,38,46,41,50,58,53,62,70,65)]
  #1423 (eop,low,ao,cl) 
  if(auc[i,"auc1"]>auc[i,"auc2"] & auc[i,"auc1"]>auc[i,"auc3"] & auc[i,"auc1"]>auc[i,"auc4"]
     & auc[i,"auc3"]>auc[i,"auc4"] & auc[i,"auc3"]>auc[i,"auc2"] & auc[i,"auc4"]>auc[i,"auc2"])
    pcd[i,1:18] <- pcd.all[i,c(3,8,11,15,20,23,27,32,35,39,44,47,51,56,59,63,68,71)] 
  #1432 (eop,low,cl,ao)
  if(auc[i,"auc1"]>auc[i,"auc2"] & auc[i,"auc1"]>auc[i,"auc3"] & auc[i,"auc1"]>auc[i,"auc4"]
     & auc[i,"auc4"]>auc[i,"auc3"] & auc[i,"auc4"]>auc[i,"auc2"] & auc[i,"auc3"]>auc[i,"auc2"])
    pcd[i,1:18] <- pcd.all[i,c(3,11,8,15,23,20,27,35,32,39,47,44,51,59,56,63,71,68)] 
  #2134 (ao,eop,cl,low)
  if(auc[i,"auc2"]>auc[i,"auc1"] & auc[i,"auc2"]>auc[i,"auc3"] & auc[i,"auc2"]>auc[i,"auc4"]
     & auc[i,"auc1"]>auc[i,"auc3"] & auc[i,"auc1"]>auc[i,"auc4"] & auc[i,"auc3"]>auc[i,"auc4"])
    pcd[i,1:18] <- pcd.all[i,c(4,1,7,16,13,19,28,25,31,40,37,43,52,49,55,64,61,67)]
  #2143 (ao,eop,low,cl)
  if(auc[i,"auc2"]>auc[i,"auc1"] & auc[i,"auc2"]>auc[i,"auc3"] & auc[i,"auc2"]>auc[i,"auc4"]
     & auc[i,"auc1"]>auc[i,"auc3"] & auc[i,"auc1"]>auc[i,"auc4"] & auc[i,"auc4"]>auc[i,"auc3"])
    pcd[i,1:18] <- pcd.all[i,c(5,2,10,17,14,22,29,26,34,41,38,46,53,50,58,65,62,70)]
  #2314 (ao,cl,eop,low)
  if(auc[i,"auc3"]>auc[i,"auc1"] & auc[i,"auc3"]>auc[i,"auc2"] & auc[i,"auc3"]>auc[i,"auc4"]
     & auc[i,"auc1"]>auc[i,"auc2"] & auc[i,"auc1"]>auc[i,"auc4"] & auc[i,"auc2"]>auc[i,"auc4"])
    pcd[i,1:18] <- pcd.all[i,c(7,1,4,19,13,16,31,25,28,43,37,40,55,49,52,67,61,64)]
  #2341 (ao,cl,low,eop)
  if(auc[i,"auc4"]>auc[i,"auc1"] & auc[i,"auc4"]>auc[i,"auc2"] & auc[i,"auc4"]>auc[i,"auc3"]
     & auc[i,"auc1"]>auc[i,"auc2"] & auc[i,"auc1"]>auc[i,"auc3"] & auc[i,"auc2"]>auc[i,"auc3"])
    pcd[i,1:18] <- pcd.all[i,c(10,2,5,22,14,17,34,26,29,46,38,41,58,50,53,70,62,65)]
  #2413 (ao,low,eop,cl)
  if(auc[i,"auc3"]>auc[i,"auc1"] & auc[i,"auc3"]>auc[i,"auc2"] & auc[i,"auc3"]>auc[i,"auc4"]
     & auc[i,"auc1"]>auc[i,"auc2"] & auc[i,"auc1"]>auc[i,"auc4"] & auc[i,"auc4"]>auc[i,"auc2"])
    pcd[i,1:18] <- pcd.all[i,c(8,3,11,20,15,23,32,27,35,44,39,47,56,51,59,68,63,71)]
  #2431 (ao,low,cl,eop)
  if(auc[i,"auc4"]>auc[i,"auc1"] & auc[i,"auc4"]>auc[i,"auc2"] & auc[i,"auc4"]>auc[i,"auc3"]
     & auc[i,"auc1"]>auc[i,"auc2"] & auc[i,"auc1"]>auc[i,"auc3"] & auc[i,"auc3"]>auc[i,"auc2"])
    pcd[i,1:18] <- pcd.all[i,c(11,3,8,23,15,20,35,27,32,47,39,44,59,51,56,71,63,68)]
  #3124 (cl,eop,ao,low)
  if(auc[i,"auc2"]>auc[i,"auc1"] & auc[i,"auc2"]>auc[i,"auc3"] & auc[i,"auc2"]>auc[i,"auc4"]
     & auc[i,"auc3"]>auc[i,"auc1"] & auc[i,"auc3"]>auc[i,"auc4"] & auc[i,"auc1"]>auc[i,"auc4"])
    pcd[i,1:18] <- pcd.all[i,c(4,7,1,16,19,13,28,31,25,40,43,37,52,55,49,64,67,61)]
  #3142 (cl,eop,low,ao)
  if(auc[i,"auc2"]>auc[i,"auc1"] & auc[i,"auc2"]>auc[i,"auc3"] & auc[i,"auc2"]>auc[i,"auc4"]
     & auc[i,"auc4"]>auc[i,"auc1"] & auc[i,"auc4"]>auc[i,"auc3"] & auc[i,"auc1"]>auc[i,"auc3"])
    pcd[i,1:18] <- pcd.all[i,c(5,10,2,17,22,14,29,34,26,41,46,38,53,58,50,65,70,62)]
  #3214 (cl,ao,eop,low)
  if(auc[i,"auc3"]>auc[i,"auc1"] & auc[i,"auc3"]>auc[i,"auc2"] & auc[i,"auc3"]>auc[i,"auc4"]
     & auc[i,"auc2"]>auc[i,"auc1"] & auc[i,"auc2"]>auc[i,"auc4"] & auc[i,"auc1"]>auc[i,"auc4"])
    pcd[i,1:18] <- pcd.all[i,c(7,4,1,19,16,13,31,28,25,43,40,37,55,52,49,67,64,61)]
  #3241 (cl,ao,low,eop)
  if(auc[i,"auc4"]>auc[i,"auc1"] & auc[i,"auc4"]>auc[i,"auc2"] & auc[i,"auc4"]>auc[i,"auc3"]
     & auc[i,"auc2"]>auc[i,"auc1"] & auc[i,"auc2"]>auc[i,"auc3"] & auc[i,"auc1"]>auc[i,"auc3"])
    pcd[i,1:18] <- pcd.all[i,c(10,5,2,22,17,14,34,29,26,46,41,38,58,53,50,70,65,62)]
  #3412 (cl,low,eop,ao)
  if(auc[i,"auc3"]>auc[i,"auc1"] & auc[i,"auc3"]>auc[i,"auc2"] & auc[i,"auc3"]>auc[i,"auc4"]
     & auc[i,"auc4"]>auc[i,"auc1"] & auc[i,"auc4"]>auc[i,"auc2"] & auc[i,"auc1"]>auc[i,"auc2"])
    pcd[i,1:18] <- pcd.all[i,c(8,11,3,20,23,15,32,35,27,44,47,39,56,59,51,68,71,63)]
  #3421 (cl,low,ao,eop)
  if(auc[i,"auc4"]>auc[i,"auc1"] & auc[i,"auc4"]>auc[i,"auc2"] & auc[i,"auc4"]>auc[i,"auc3"]
     & auc[i,"auc3"]>auc[i,"auc1"] & auc[i,"auc3"]>auc[i,"auc2"] & auc[i,"auc1"]>auc[i,"auc2"])
    pcd[i,1:18] <- pcd.all[i,c(11,8,3,23,20,15,35,32,27,47,44,39,59,56,51,71,68,63)]
  #4123 (low,eop,ao,cl)
  if(auc[i,"auc2"]>auc[i,"auc1"] & auc[i,"auc2"]>auc[i,"auc3"] & auc[i,"auc2"]>auc[i,"auc4"]
     & auc[i,"auc3"]>auc[i,"auc1"] & auc[i,"auc3"]>auc[i,"auc4"] & auc[i,"auc4"]>auc[i,"auc1"])
    pcd[i,1:18] <- pcd.all[i,c(6,9,12,18,21,24,30,33,36,42,45,48,54,57,60,66,69,72)]
  #4132 (low,eop,cl,ao)
  if(auc[i,"auc2"]>auc[i,"auc1"] & auc[i,"auc2"]>auc[i,"auc3"] & auc[i,"auc2"]>auc[i,"auc4"]
     & auc[i,"auc4"]>auc[i,"auc1"] & auc[i,"auc4"]>auc[i,"auc3"] & auc[i,"auc3"]>auc[i,"auc1"])
    pcd[i,1:18] <- pcd.all[i,c(6,12,9,18,24,21,30,36,33,42,48,45,54,60,57,66,72,69)]
  #4213 (low,ao,eop,cl)
  if(auc[i,"auc3"]>auc[i,"auc1"] & auc[i,"auc3"]>auc[i,"auc2"] & auc[i,"auc3"]>auc[i,"auc4"]
     & auc[i,"auc2"]>auc[i,"auc1"] & auc[i,"auc2"]>auc[i,"auc4"] & auc[i,"auc4"]>auc[i,"auc1"])
    pcd[i,1:18] <- pcd.all[i,c(9,6,12,21,18,24,33,30,36,45,42,48,57,54,60,69,66,72)]
  #4231 (low,ao,cl,eop)
  if(auc[i,"auc4"]>auc[i,"auc1"] & auc[i,"auc4"]>auc[i,"auc2"] & auc[i,"auc4"]>auc[i,"auc3"]
     & auc[i,"auc2"]>auc[i,"auc1"] & auc[i,"auc2"]>auc[i,"auc3"] & auc[i,"auc3"]>auc[i,"auc1"])
    pcd[i,1:18] <- pcd.all[i,c(12,6,9,24,18,21,36,30,33,48,42,45,60,54,57,72,66,69)]
  #4312 (low,cl,eop,ao)
  if(auc[i,"auc3"]>auc[i,"auc1"] & auc[i,"auc3"]>auc[i,"auc2"] & auc[i,"auc3"]>auc[i,"auc4"]
     & auc[i,"auc4"]>auc[i,"auc1"] & auc[i,"auc4"]>auc[i,"auc2"] & auc[i,"auc2"]>auc[i,"auc1"])
    pcd[i,1:18] <- pcd.all[i,c(9,12,6,21,24,18,33,36,30,45,48,42,57,60,54,69,72,66)]
  #4321 (low,cl,ao,eop)
  if(auc[i,"auc4"]>auc[i,"auc1"] & auc[i,"auc4"]>auc[i,"auc2"] & auc[i,"auc4"]>auc[i,"auc3"]
     & auc[i,"auc3"]>auc[i,"auc1"] & auc[i,"auc3"]>auc[i,"auc2"] & auc[i,"auc2"]>auc[i,"auc1"])
    pcd[i,1:18] <- pcd.all[i,c(12,9,6,24,21,18,36,33,30,48,45,42,60,57,54,72,69,66)]}
```

If we take a look at the results below, we can see the log risk ratio (SE) for the total effect of EOP versus Low = 0.42 (0.08), the indirect effect of EOP versus Low = 0.07 (0.03), and the direct effect of EOP versus Low = 0.34 (0.08). The class probabilities are: EOP = 10%, AO = 10%, CL = 12%, Low = 69%.

```
(as.data.frame(pcd))
```

```
#now we can put results in a table and export
write.table(pcd, file="upcd.txt", sep = ",")
```
